# Deciphering the Role of Different Ceramide Synthases in the Human Cardiomyocyte Hypertrophic Response

**DOI:** 10.3390/metabo15090635

**Published:** 2025-09-22

**Authors:** Alexandra M. Wiley, Melissa A. Krueger, Nona Sotoodehnia, Jason G. Umans, Andrew N. Hoofnagle, Rozenn N. Lemaitre, Rheem A. Totah, Sina A. Gharib

**Affiliations:** 1 Department of Medicinal Chemistry, University of Washington, Seattle, WA 98195, USA; alex.wiley@merck.com; 2 Computational Medicinal Core, Center for Lung Biology, Division of Pulmonary, Critical Care and Sleep Medicine, Department of Medicine, University of Washington, Seattle, WA 98109, USA; krueger@uw.edu (M.A.K.); sagharib@uw.edu (S.A.G.); 3 Cardiovascular Health Research Unit, Department of Medicine, Division of Cardiology, University of Washington, Seattle, WA 98195, USA; nsotoo@uw.edu; 4 MedStar Health Research Institute, Hyattsville, MD 21044, USA; jgu@georgetown.edu; 5 Department of Laboratory Medicine, University of Washington, Seattle, WA 98195, USA; ahoof@uw.edu; 6 Cardiovascular Health Research Unit, Department of Medicine, University of Washington, Seattle, WA 98195, USA; rozenl@uw.edu

**Keywords:** cell signaling, ceramides, lipids, sphingolipids, cardiovascular disease, cardiac hypertrophy, transcriptomics

## Abstract

**Background/Objectives**: Recent studies suggest that plasma ceramide levels may be better predictors of CVD risk than LDL cholesterol. Ceramides are part of the sphingolipid class of lipids and are the central intermediates in complex sphingolipid biosynthesis. Sphingolipids are crucial for cellular structure and have important biological roles as complex signaling lipids, structurally and functionally differentiated by their acylated fatty acid. Higher plasma concentrations of 16:0 ceramide are associated with increased risk of heart failure. In contrast, higher concentrations of 22:0 plus 24:0 ceramide are associated with lower risk. We aim to address how alterations in these lipids can affect the human cardiac hypertrophic response. **Methods**: We silenced the ceramide synthase genes (*CERS*) responsible for the production of 16:0 ceramide (*CERS5*/*6*) or 22:0 and 24:0 ceramide (*CERS2*) in immortalized human ventricular cardiomyocytes and examined the altered cardiac hypertrophic response to phorbol 12-myristate 13-acetate treatment by examining changes in the transcriptome. **Results**: We discovered that silencing *CERS2* or *CERS5*/*6* drastically altered the cardiac cell hypertrophic response. We demonstrated that human cardiomyocytes with silenced *CERS2* appeared to have an exacerbated hypertrophy response, while cardiomyocytes with silenced *CERS5*/*6* had a more favorable response, suggesting that *CERS2* and *CERS5*/*CERS6* and their gene product metabolites may have opposing roles in the development and progression of CVD. **Conclusions**: The exact mechanisms through which various ceramides contribute to CVD progression are still unknown. This study will help elucidate the role of specific ceramides during cardiac hypertrophy and suggests that drugs targeting specific sphingolipids can potentially be a viable treatment option for the prevention of CVD.

## 1. Introduction

Cardiovascular disease (CVD) is the leading cause of global morbidity and mortality [1]. The proportion of deaths and disabilities due to CVD has been steadily rising across the globe [1,2], driving a growing urgency to discover and study novel therapeutic targets to combat CVD and its devastating effect on the quality of life. Today, LDL (low-density lipoprotein) cholesterol levels represent one of the best predictors for CVD risk, but there is evidence to suggest that plasma ceramides can be more accurate predictors than LDL cholesterol [3,4,5,6]. Although there is an extensive body of literature to support the role of cholesterol in heart failure (HF), the role of ceramides in HF disease progression is still poorly understood.

Ceramides are members of the sphingolipid class and constitute the central metabolites needed for the synthesis of more complex sphingolipids, such as sphingomyelin (SM). Ceramides contain a sphingosine backbone with an acylated fatty acid of varying chain lengths, and different ceramides are linked to different biological functions [7]. Additionally, ceramides play a role in several processes of relevance to HF pathophysiology, including promoting apoptosis, oxidative stress, endothelial dysfunction, inflammation, lipotoxicity, and insulin resistance [8,9,10]. While early studies on ceramides considered these biological activities to apply to all ceramides, over 200 species of ceramide are reported [11], and investigating the specific roles that each ceramide plays in health is needed. Recent research focused on defining the roles of ceramides with different fatty acid lengths may play in CVD. Since ceramide synthesis and metabolism are complex and involve several compensatory pathways [12,13,14], elucidating the distinct functions of different acyl-chain length ceramides in experimental systems is essential.

In the Cardiovascular Health Study (CHS), a large prospective study among older adults, we reported that long-chain (LC) 16:0 plasma ceramide and SM are associated with an increased risk of incident HF, while very long-chain (VLC) 22:0 ceramide, and 20:0, 22:0, and 24:0 SM were associated with a decreased risk of HF [3]. We hypothesize that modulating the levels of certain sphingolipid species can influence CVD progression and alter biological processes related to HF. In this report, we explore the effect silencing the ceramide synthases, *CERS2* or *CERS5*/*6*, has on human cardiomyocytes’ hypertrophic response. We hypothesize that the lipid products generated by CerS2—VLC ceramides—are protective during cardiac hypertrophy, while the products of CerS5/6—LC ceramides—promote the progression and development of hypertrophy. We introduce and validate a working hypertrophy model in cardiomyocytes, and using this model, we perform global transcriptomics to investigate the pathways altered due to silencing different *CERS* and inducing hypertrophy. This work is an important first step to uncovering mechanisms and pathways underlying the association of different ceramide species with the risk of heart failure.

## 2. Materials and Methods

### 2.1. Materials

The immortalized human ventricular cardiomyocytes and all consumables needed to maintain the myocytes, including PriGrow I media, penicillin/strep, and extracellular matrix, were purchased from Applied Biological Materials (Bellingham, WA, USA). CERS2 siRNA and the universal scramble control (#SR323951) were purchased from Origene (Rockville, MD, USA). Silencer siRNA for CERS5 (#AM16708, siRNA ID #131807), CERS6 (#AM16708, siRNA ID #149485), and Silencer Negative Control #5 siRNA (#AM4642) were obtained through Invitrogen (Waltham, MA, USA). Tissue culture treated plates, Lipofectamine 3000 Reagent, DMSO, Opti-MEM media, PBS, formaldehyde, Tween 20, Hoechst 33342 (#62249), Alexa Fluor 488 Phalloidin (#A12379), cell scrapers, RIPA lysis buffer, Halt protein and phosphatase inhibitor cocktail, BCA reagents and standards, dithiothreitol, electrophoresis system, PageRuler Plus Prestained Protein Ladder, NuPAGE 4–12% Bis-Tris gels, MOPS SDS Running Buffer, iBlot Transfer System, nitrocellulose transfer stacks, and RNA isolation kits were all obtained from Thermo Fisher Scientific (Waltham, MA, USA). The anti-B-type natriuretic peptide (BNP) antibody was purchased from Bioss Antibodies (Woburn, MA, USA), while the anti-β-actin antibody was purchased from Cell Signaling (Danvers, MA, USA). The secondary antibodies were obtained from LI-COR (Lincoln, NE, USA). Phorbol 12-myristate 13-acetate was purchased from Cayman Chemicals (Ann Arbor, MI, USA), and Triton X-100 was purchased from Sigma Aldrich (Burlington, MA, USA).

### 2.2. Cell Culture

Immortalized human ventricular cardiomyocytes (HCMs) (#T0519) were maintained according to the manufacturer’s recommendations; in Prigrow I medium (#TM001) supplemented with 10% fetal bovine serum (FBS) and 0.1% Penicillin/Streptomycin Solution (#G255). We investigated several different ventricular cell lines, including AC-16, but chose these ventricular immortalized cells since RNA sequencing suggested a healthier ventricular cardiomyocyte phenotype as indicated by a more physiologically relevant expression of cardiac markers: *GATA4*, *ACTB*, *SMAD2*, *VEGFB*, *NPPB*, *CAMK2D*, and *CLCN3*. Additionally, the baseline expression of *NPPB* allowed us to use this clinical biomarker to repor on hypertrophic cells, thus giving us the ability to compare results between sample types prior to sending samples for RNA sequencing [15]. We did not choose to work with iPSC-derived cardiomyocytes due to their immature nature, which will complicate data analysis. Cells were kept in a humidified incubator at 37 °C with 5% CO_2_.

### 2.3. Hypertrophy Induction with CERS2 and CERS5/6 Knockdown (KD)

Gene silencing was accomplished following the same protocol and using materials described in our previous manuscript [15]. After the 24 h siRNA treatment, cells were treated with 4 µM phorbol 12-myristate 13-acetate (PMA) or a comparative vehicle control (0.025% DMSO) in serum-free media. With the *CERS5*/*6* combo KD and its respective control, HCMs were retreated with 2 µM PMA 24 h after the start of the initial treatment, leading to a 48 h hypertrophy treatment, while the initial PMA treatment was similar to *CERS2* KD to minimize cell death. After completing 48 h of PMA treatment, the cells were harvested for further analyses described below.

### 2.4. Cell Imaging

Following 48 h of PMA treatment, cells were washed and fixed with 4% methanol-free formaldehyde for 20 min (#FB002, Thermo Fisher Scientific, Waltham, MA, USA). Cells were then permeabilized with 0.1% Triton X-100 (#T8787) in PBS prior to staining for 45 min in the dark. The staining solution consisted of Hoechst 33342 to stain for the nucleus, and Alexa Fluor 488 Phalloidin (Thermo Fisher Scientific) to stain for F-actin, using the manufacturer’s recommended dilutions. Images were obtained at random using an EVOS M7000 Microscope (Thermo Fisher Scientific) at 40x Following image procurement, images were analyzed using Celleste Image Analysis Software Version 6.0. An automated method for obtaining cell area was created, where the sum of the membrane area was divided by the number of nuclei present, generating an average cell area for each respective image.

### 2.5. Immunoblotting

Following induction of hypertrophy, samples were lysed with RIPA lysis buffer (#89901, Thermo Fisher Scientific) and Halt Protease and Phosphatase Inhibitor Cocktail (#78440, Thermo Fisher Scientific). To normalize the protein content of each sample, BCA protein reagents (Reagent A, #23228, Thermo Fisher Scientific; Reagent B, #23224, Thermo Fisher Scientific) and the Bovine Serum Albumin Pre-Diluted Standard Set (#23208, Thermo Fisher Scientific) were used. Samples were then reduced with 50 mM dithiothreitol (#A39255, Thermo Fisher Scientific) and left to incubate at room temperature for 15 min. NuPAGE 4–12% Bis-Tris gels (#NP0336, Thermo Fisher Scientific) were used with the XCell SureLock Mini-Cell Electrophoresis system (Thermo Fisher Scientific), PageRuler Plus Prestained Protein Ladder (#26619, Thermo Fisher Scientific), and MOPS SDS Running Buffer (#NP0001, Thermo Fisher Scientific). The gel was transferred to a nitrocellulose transfer stack (#IB301001, Thermo Fisher Scientific) using an iBlot Transfer System (Thermo Fisher Scientific). For 90 min, the blot was blocked in blocking buffer consisting of: 5% *w*/*v* milk powder, 5% *w*/*v* BSA, 0.1% *v*/*v* Tween 20, and 0.1% *w*/*v* sodium azide in PBS. A primary antibody incubation with a 1:1000 dilution of rabbit anti-β-actin antibody (#4970, Cell Signaling Technology, Danvers, MA, USA) and a 1:500 dilution of mouse anti-BNP antibody (#bsm-4579M-A647, Bioss Antibodies, Woburn, MA, USA) was conducted overnight. This was followed by an hour-long room temperature incubation with secondary IRDye 680RD goat anti-rabbit (#926-68071, LI-COR Biosciences, Lincoln, NE, USA) and IRDye 680RD goat anti-mouse (#926-68070, LI-COR Biosciences) antibodies the next morning. Western blots were scanned using an Odyssey CLx gel scanner (LI-COR Biosciences), and images were visualized using Image Studio Version 4.0 software.

### 2.6. Total RNA Isolation

Following 24 h siRNA incubation and 48 h hypertrophic treatment, cells were rinsed once with ice-cold PBS and lysed with TRI Reagent. Total RNA isolation was achieved by utilizing the MagMax-96 for Microarrays Total RNA Isolation Kit (#AM1839, Thermo Fisher Scientific) following the spin procedure.

### 2.7. Library Preparation and mRNA-Sequencing

Total RNA was shipped to Novogene Corporation Inc. (Sacramento, CA, USA), where samples underwent library preparation and mRNA-sequencing. Samples underwent polyA capture to enrich mRNA, then were converted to cDNA, and sequenced with Illumina PE150 technology. Samples were subjected to three quality control checks prior to analysis. After sequencing, samples were aligned and validated to map to over 90% of the human reference genome GRCh38. One of the scramble controls for the *CERS2* KD did not meet these standards and was therefore excluded from further analysis.

### 2.8. Bioinformatics and Pathway Analyses

#### 2.8.1. Gene Expression Analysis

Low-abundance genes were filtered by removing those with raw counts below 10 in all samples. The DESeq2 (v1.48.0) package in R was then utilized to identify differentially expressed genes between the various treatment groups and controls using a Benjamini-Hochberg adjusted *p*-value < 0.05 threshold [16].

#### 2.8.2. Gene Set Enrichment Analysis (GSEA)

GSEA [17,18] utilizing two Molecular Signature Database categories, Hallmark and Canonical Pathways [19,20], was applied to identify biological pathways altered by PMA treatment using the entire transcriptome rank-ordered based on DESeq2’s test statistic [16]. Significant gene sets were determined at a false discovery rate (FDR) < 0.05.

#### 2.8.3. LRT/Cluster Analysis

To investigate the genes associated with the interaction between PMA treatment and genotype, we employed a likelihood ratio test (LRT) within DESeq2 [16]. Specifically, we compared the full model, including the interaction term, to a reduced model that excluded the interaction term. Genes that showed significant evidence of an interaction between PMA treatment and genotype (adjusted *p*-value < 0.05) were then clustered based on their expression patterns across all experimental conditions using the k-means algorithm. The number of clusters was chosen to minimize intra-group variance while maximizing the silhouette score, which measures how similar an object is to its own cluster compared to other clusters.

#### 2.8.4. Pathway Over-Representation Analysis

In order to identify pathway changes due to hypertrophic conditions in the context of *CERS* KD, we utilized WebGestalt’s over-representation analysis software [21]. Using a threshold of FDR < 0.05 and canonical pathway databases (KEGG, Panther, and Reactome), pathway enrichment results were generated for the entirety of the differentially expressed LRT gene lists for both *CERS2* and *CERS5*/*6* KD. Additionally, pathway enrichment analyses were performed for differentially expressed gene lists mapping to distinct k-means clusters.

## 3. Results

### 3.1. Establishing a Cell Culture Model of Hypertrophy

Cardiac hypertrophy is a condition characterized by enlarged cardiomyocytes and ventricular wall thickening, resulting in impaired heart function and ultimately HF [22]. Since HF cannot be reproduced in a cell model, we developed a cardiac hypertrophy model in culture to serve as a tool to investigate molecular perturbations leading to HF. We believe this represents a valuable, testable approach to investigate the role of 16:0, 22:0, and 24:0 ceramide species in the heart’s response to CVD progression.

Since imaging experiments are not conducive to complex comparisons between experimental groups, we used B-type natriuretic peptide (BNP) as our biochemical marker for hypertrophy, which is currently the clinical gold standard plasma biomarker for HF. We used phorbol 12-myristate 13-acetate (PMA) as our hypertrophy-inducing agent because 48 h PMA treatment resulted in dose-dependent and consistent increases in NPPB (gene encoding for BNP) expression.

**Figure 1:**
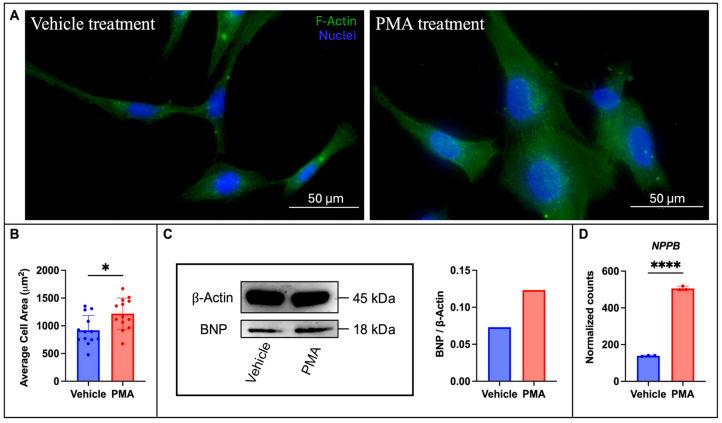
PMA treatment for 48 h induces a hypertrophic response in HCMs. (**A**) An increase in HCM cell size is observed with 40x images and F-actin staining, and the (**B**) average cell area from 13 individual images depicts a roughly 33% increase in HCM size. (**C**) Western blot and the respective band intensity quantification depicting increased BNP protein expression (*n* = 6, technical replicates) and (**D**) *NPPB* (gene encoding for BNP) normalized gene expression (from RNA-sequencing results) increases following PMA treatment, *n* = 3. **** *p* < 0.0001, * *p* < 0.05.

Following PMA or vehicle control treatment, HCMs were fixed, stained, and imaged to analyze changes in cell size (Figure 1A). PMA treatment increased the average cell size by approximately 33% (Figure 1B). Additionally, PMA treatment increased both BNP protein expression (Figure 1C) and mRNA (Figure 1D). Taken together, this experimental evidence supports that PMA treatment induces a consistent cardiac hypertrophy response in our HCM model.

### 3.2. Leveraging Transcriptomics to Delineate the Role of Ceramides in HF

Previously, we demonstrated that an 80% KD of either *CERS2* or *CERS5*/*6* in HCMs resulted in a significant reduction in VLC and LC ceramides, respectively, as well as their corresponding downstream complex sphingolipids [15]. Furthermore, we observed that with *CERS* KD alone, many pathways are altered, and interestingly, many of these differentially enriched pathway changes were discordant when comparing the *CERS2* KD to the *CERS5*/*6* KD. This previous work suggests that ceramides play contrasting roles in HCM homeostasis, with LC ceramides possibly contributing to cardiac dysfunction, and VLC ceramides presenting potential protective benefits.

**Figure 2:**
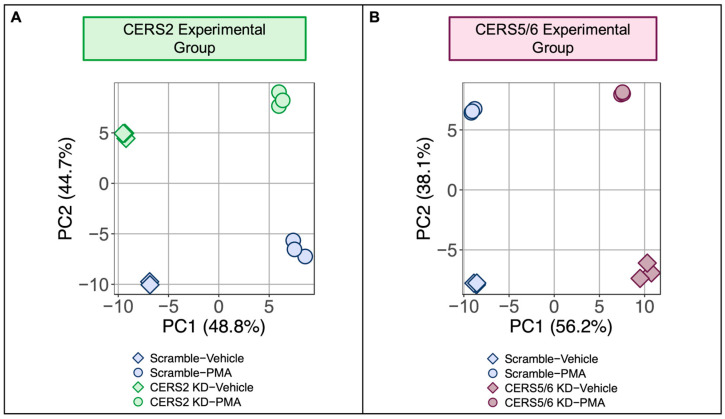
Global transcriptomic variation between the different *CERS* experimental groups treated with PMA vs controls. PCA plot across the cardiomyocyte transcriptome with the samples from the (**A**) *CERS2* KD and (**B**) *CERS5*/*6* KD demonstrates clear separation between all the different sample conditions in each experimental group.

In this report, we investigate how altering LC and VLC sphingolipid species in HCMs influences CVD progression by silencing their respective *CERS* enzymes and inducing cellular hypertrophy with PMA. Our two experimental groups comprise silencing of the ceramide synthases responsible for the generation of the proposed protective VLC ceramides (*CERS2*), and detrimental LC 16:0 ceramide (*CERS5*/*6*). This resulted in four different conditions (*n* = 3) for each *CERS* KD that were subjected to bulk mRNA sequencing: (1) scramble control treated with vehicle, (2) scramble control treated with PMA, (3) KD treated with vehicle, (4) KD treated with PMA. Principal component analysis (PCA) plots display separation between the four sample conditions with *CERS2* KD (Figure 2A) and *CERS5*/*6* KD (Figure 2B), indicating that both KD and PMA treatments alone lead to significant alterations in the transcriptome.

### 3.3. Hypertrophy-Specific Responses in Human Cardiomyocytes

**Figure 3:**
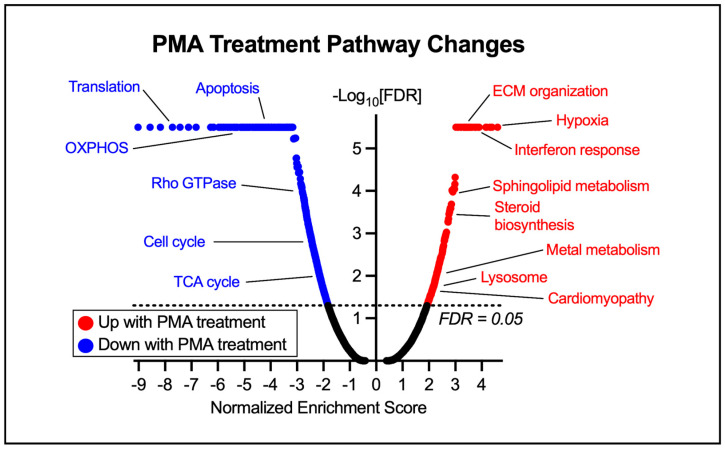
Volcano plot displaying pathway changes due to 48 h PMA treatment. The highlighted pathways are representative of the major changes in HCMs observed in scramble controls treated with PMA compared to those treated with vehicle controls. OXPHOS—oxidative phosphorylation, TCA—tricarboxylic acid. A full list of altered pathways is included in Supplemental Table S1.

We initially compared the transcriptional response of HCMs treated with PMA for 48 h to vehicle-treated controls. We applied DESeq2 to identify 3743 differentially expressed genes (DEGs) (adjusted *p*-value < 0.05) (2031 up and 1712 down) following exposure to hypertrophy-inducing PMA. With PMA treatment, we observed an upregulation of gene sets involved in extracellular matrix (ECM) organization, interferon response, hypoxia, sphingolipid and metal metabolism, and steroid biosynthesis (Figure 3). Additionally, we observed an upregulation of cardiomyopathy pathways, suggesting that the changes observed due to PMA treatment render, at least in part, similar changes to those observed in HF. Furthermore, we saw downregulations of gene sets involved in translation, apoptosis, energy metabolism, rho GTPase, and the cell cycle.

### 3.4. Hypertrophic Response Alterations with Ceramide Synthase 2 (CERS2) Knockdown

Reducing the expression of *CERS2* altered the PMA-induced hypertrophic response. We applied LRT analysis to identify 560 genes (adjusted *p*-value < 0.05) that were significantly altered due to an interaction between PMA treatment and *CERS2* KD. Importantly, none of the other *CERS* genes were identified, validating that with the KD and PMA treatment, *CERS* expression stayed constant.

**Figure 4:**
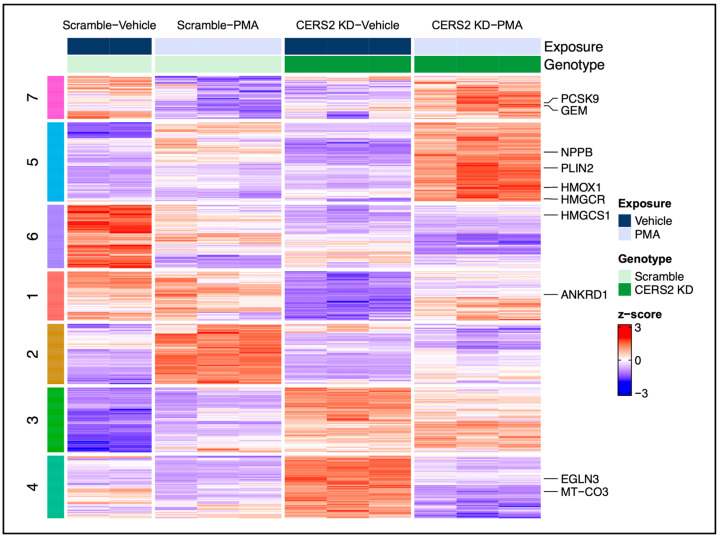
Heat map depiction of differential gene expression cluster patterns in the hypertrophic response due to *CERS2* KD (PMA × *CERS2* KD interaction). The color coding and numbers on the left map onto the specific gene clusters presented in Figure 5 below. A comprehensive list of the identified genes and their respective clusters can be found in Supplemental Table S2.

**Figure 5:**
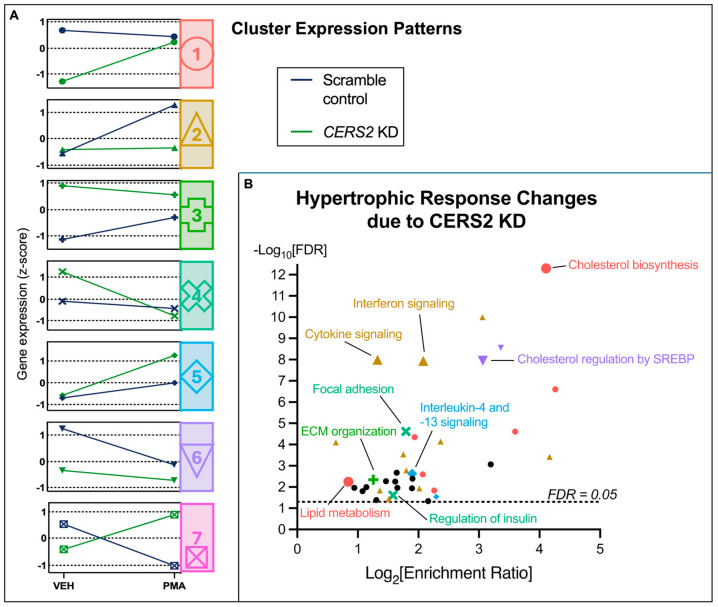
Biological functions assigned to each gene cluster identified as being significantly different in the *CERS2* KD hypertrophy response. (**A**) Identified cluster expression patterns for all the gene changes due to PMA treatment and *CERS2* KD. (**B**) WebGestalt analysis using all 560 genes identified as different in the hypertrophic response due to *CERS2* KD. Representative biological functions were assigned to each cluster, with the symbol and color coordinating to the respective cluster (FDR < 0.05). Black circles indicate enriched gene sets that did not map to any of the respective clusters shown. ECM—extracellular matrix, SREBP—sterol regulatory element-binding proteins. A full list of pathway changes and their respective clusters can be found in Supplemental Table S3.

To delineate the expression patterns of these DEGs across the four experimental conditions, we performed k-means clustering and found that the transcriptional profiles were well-captured by 7 distinct clusters (Figure 4). To add biological interpretation to these analyses, we initially performed pathway over-representation analysis on all 560 DEGs. Then, the gene lists comprising each cluster were subjected to this same analysis, and any identified overlapping functions were labeled according to their respective cluster (Figure 5). Interestingly, we observed hypertrophic response changes in cholesterol and lipid metabolism, the immune response, and ECM organization due to *CERS2* KD. Collectively, alterations in these pathways suggest that the change in the hypertrophic response due to silencing *CERS2* involves important processes in CVD pathophysiology.

### 3.5. Hypertrophic Response Alterations with Ceramide Synthase 5 and 6 (CERS5/6) Knockdown

**Figure 6:**
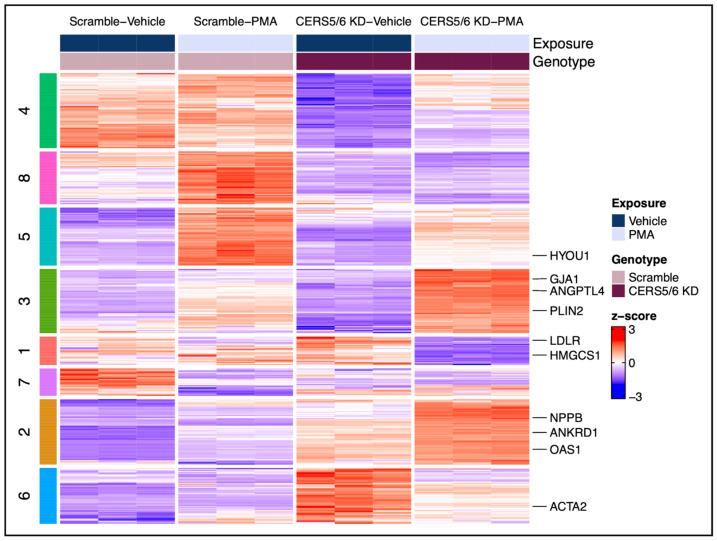
Heat map depiction of differential gene expression cluster patterns the hypertrophic response due to *CERS5*/*6* KD (*CERS5*/*6* × PMA interaction). The breakup of the genes on the left-hand side corresponds to each respective gene cluster presented in Figure 7. A complete gene list and respective clusters can be found in Supplemental Table S4.

Similar to the *CERS2* KD interaction analysis, we conducted a *CERS5*/*6* KD interaction analysis to examine how genetic manipulation of *CERS5*/*6* influenced the hypertrophic response to PMA. Controlling for the response to both *CERS5*/*6* KD and PMA treatments alone identified 405 genes (adjusted *p*-value < 0.05) as being different in the hypertrophic response due to *CERS5*/*6* KD. Notably, none of the *CERS* were identified within these 405 genes of interest, again verifying that with KD and PMA treatment, other *CERS* expression stayed consistent. *LDLR* (low-density lipoprotein receptor), *ACTA2* (actin alpha 2), *PLIN2* (perilipin 2), and *ANKRD1* (ankyrin repeat domain 1) constitute a few of these observed gene changes, annotated in Figure 6.

**Figure 7:**
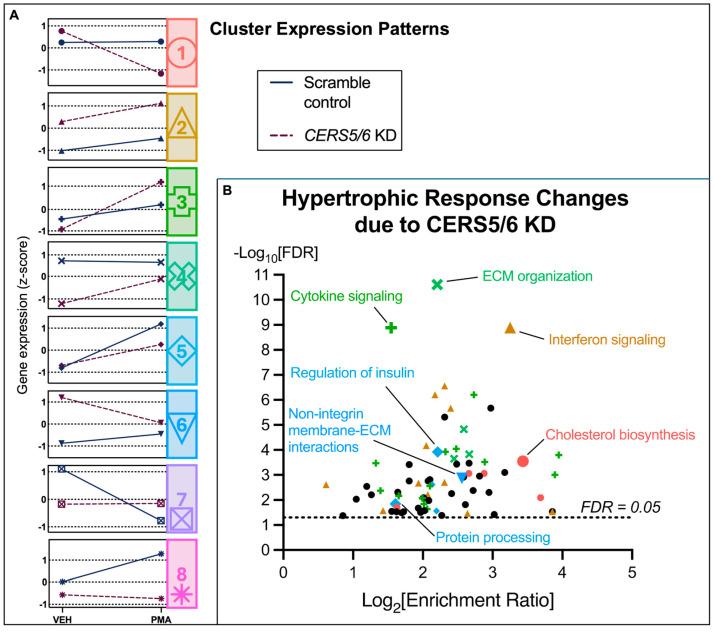
Biological functions assigned to each gene cluster identified as being significantly different in the *CERS5*/*6* KD hypertrophy response. (**A**) Identified cluster expression patterns for all the gene changes due to both *CERS5*/*6* KD and PMA treatment. (**B**) WebGestalt analysis using all 405 genes identified as different (FDR < 0.05) in the hypertrophic response due to *CERS5*/*6* KD. Unlabeled black circles indicate enriched gene sets that did not correspond to a respective cluster. ECM—extracellular matrix. A comprehensive list of pathway changes and clusters can be found in Supplemental Table S5.

K-means clustering was performed on these 405 DEGs (Figure 6), and pathway enrichment analysis was undertaken for each identified cluster (Figure 7). Similar to what was found with *CERS2* KD, we observed hypertrophic response changes in cholesterol biosynthesis, the immune system, and ECM organization due to *CERS5*/*6* KD. These results suggest that the changes in the hypertrophic response due to *CERS5*/*6* KD also involve important pathways that could further impact CVD progression.

## 4. Discussion

In this study, we investigated the molecular consequences of silencing ceramide synthases responsible for the production of VLC 22:0 and 24:0 ceramides (*CERS2*) and LC 16:0 ceramide (*CERS5*/*6*) on the hypertrophic response in human cardiomyocytes. It is generally accepted that increased LC ceramide 16:0 is detrimental to health as many studies presented evidence demonstrating its role in metabolic dysfunction, insulin resistance, and decreased mitochondrial function, while the exact role for VLC ceramides, 22:0 and 24:0, has been debated as to whether they are beneficial or benign, depending on cell state [13,23,24,25,26]. We previously demonstrated that knocking down *CERS2* and *CERS5*/*6* in HCMs reduced the cellular level of their respective ceramides and downstream complex sphingolipids as expected [15]. Additionally, we found that *CERS2* and *CERS5*/*6* KD had contrasting effects on various transcriptomic pathways, which is further evidence that VLC and LC sphingolipids play different roles in cardiovascular homeostasis, and that VLC ceramides may play a protective role in CVD health, while LC ceramides may influence disease progression [15]. In this study, we expanded on our previous work by exploring how altering these ceramide synthases could impact pathways crucial for cardiovascular disease progression by utilizing a cardiac hypertrophy model. The key finding in this study is that *CERS2* KD leads to pathway changes that support a more severe HF progression, while *CERS5*/*6* KD leads to less aggressive changes in pathways crucial to cardiac hypertrophy progression. These findings support our hypothesis that products of *CERS2* may be protective against cardiac hypertrophy, while products of *CERS5*/*6* may worsen hypertrophy.

### 4.1. PMA Treatment Leads to a Cardiac Hypertrophy Phenotype

In order to test how the different ceramide synthases impact the progression of heart disease, we initially had to develop a CVD model in HCMs. We chose to target cardiac hypertrophy since hypertrophy typically precedes more advanced stages of HF, and it can be modeled in cell culture. We used the Protein Kinase C (PKC) activator, PMA [27], as our hypertrophy inducer. PKC plays a multifaceted role in many different CVDs, including cardiac hypertrophy, where it regulates apoptosis, endothelial function, cardiac ion channels, mitochondrial function, and the inflammatory response [28]. Furthermore, in 2012, Russo et al. demonstrated that *CERS5* KD was sufficient to prevent myristate-induced hypertrophy in feline cardiomyocytes [29], supporting that PMA-induced hypertrophy is a relevant model for studying the contribution of different ceramide species to the hypertrophic response. We were able to confirm the characteristic increase in HCM size with PMA treatment (Figure 1A), as well as demonstrate an increase in both mRNA (Figure 1D) and protein expression (Figure 1C) of BNP, the clinical HF biomarker.

PMA treatment alters crucial pathways consistent with cardiac hypertrophy (Figure 3). We observed marked increases in pathways involved in ECM organization and hypoxia, suggesting the PMA-treated HCMs are undergoing cardiac remodeling, a hallmark of cardiac hypertrophy [30,31]. Additionally, we noted increases in sphingolipid and metal metabolism as well as steroid biosynthesis. These changes are characteristic of CVD pathophysiology, which is further validated by the observation of enriched cardiomyopathy pathways, suggesting that our in vitro HCM model indeed captures some of the same molecular alterations as those observed in human heart disease. Observed declines in pathways involved in energy metabolism, like oxidative phosphorylation (OXPHOS) and the tricarboxylic acid (TCA) cycle, suggest the PMA-treated HCMs may have deficient energetic production that is essential for healthy HCMs, and further supports a hypertrophic phenotype. Additionally, adult HCMs typically do not reenter the cell cycle when exposed to growth signals, and instead respond to these signals by increasing cardiac mass through hypertrophy [32]. The observed decline in pathways involved in the cell cycle and translation may suggest that the HCMs are struggling to maintain homeostasis compared to their untreated controls. Collectively, the observed dysregulation in the PMA-treated HCMs is indicative of stressed and unhealthy cardiomyocytes and suggests that exposure to PMA changes the transcriptome in cultured HCMs in a manner similar to human cardiac hypertrophy, further validating the use of this model to study HF disease progression.

### 4.2. Hypertrophy Response Changes Due to CERS KD

**Figure 8:**
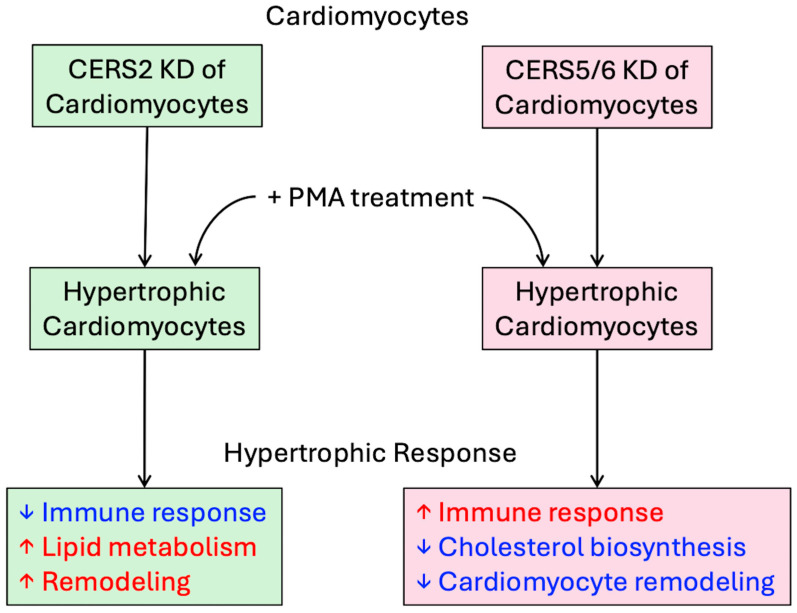
Schematic showing the transcriptomic changes due to both *CERS* KD and induction of hypertrophy.

To tease out hypertrophy response changes due to *CERS* KD, we ran an interaction analysis to control for changes specifically due to both *CERS* KD and PMA treatment. Notably, although this analysis was limited to a small number of genes within each pathway, it directed us to the most important changes in the hypertrophic response due to *CERS* KD. With the silencing of *CERS2* and *CERS5*/*6*, we identified 560 and 405 genes altered, respectively, that represent the hypertrophy response changes due to reduced *CERS* expression. We conducted cluster analyses on the identified genes and pathway analyses on the resulting clusters to assign biologically relevant functional categories to each cluster expression pattern. The results are summarized in Figure 8, and further described below.

#### 4.2.1. The Effect of *CERS2* KD on the Hypertrophic Response

Recently, we reported that with *CERS2* KD alone, we observed changes in many pathways crucial for worsening cardiac hypertrophy, suggesting *CERS2* KD and the corresponding reduction of cellular levels of VLC sphingolipids, by itself, influence a HF phenotype within these cells [15]. With the combination of *CERS2* KD and PMA treatment, the cells are being stressed twice, both with treatments that increase hypertrophy. In the interaction analysis, we identified 560 genes that were changed due to the hypertrophic response to *CERS2* silencing.

The hypertrophy response with PMA and *CERS2* KD differs from the hypertrophic response with PMA alone, as indicated by increases in pathways involved in lipid metabolism and cardiomyocyte remodeling and decreases in the immune response pathways (Figure 5B). Cluster 1 represents cholesterol and lipid metabolism. As discussed in our previous publication, *CERS2* KD alone results in decreases in pathways involved in cholesterol biosynthesis and lipid metabolism compared to control cells [15]. The marked increase in gene expression related to cholesterol biosynthesis and lipid metabolism with *CERS2* KD and PMA treatment was not observed in the scramble control treated with PMA, and was increased compared to KD alone, suggesting that with *CERS2* KD and PMA treatment, the cells are undergoing further lipid dysregulation. Additionally, we observed an increase in ECM organization, denoted by a higher gene expression profile with PMA treatment and *CERS2* KD in cluster 3, indicating increased cardiac remodeling and hypertrophy [31]. The immune response is also greatly modulated by *CERS2* KD and PMA treatment. By examining cluster 2, we observe lower gene expression changes with PMA treatment in the *CERS2* KD samples, compared to the PMA-treated scrambled controls, leading us to conclude that there is less of an immune response due to *CERS2* KD following PMA treatment. Overall, the hypertrophy response changes due to *CERS2* KD are very suggestive of increased cardiac dysfunction, but these findings need to be further explored experimentally.

#### 4.2.2. The Effect of *CERS5*/*6* KD on the Hypertrophic Response

Contrasting the *CERS2* KD, in our previous paper, we discussed how *CERS5*/*6* KD appeared to have less of an HF phenotype compared to controls, suggesting that *CERS5*/*6* and their gene products could play fundamental roles in the development and progression of HF [15]. If *CERS5*/*6* KD is protective, and PMA treatment is detrimental, it is interesting to consider which hypertrophy responses are augmented due to a decline in 16:0 sphingolipid products. Here, we identified 405 genes altered in response to both *CERS5*/*6* KD and PMA treatment.

The hypertrophic response to PMA with *CERS5*/*6* KD differs from the response to PMA in controls by the observed increases in the immune response and decreases in cholesterol biosynthesis and cardiomyocyte remodeling (Figure 7B). Clusters 2 and 3 contain gene sets imperative to the cellular immune response. With both these clusters, we observed a greater change in gene expression following PMA treatment with *CERS5*/*6* KD compared to controls. Additionally, we observed a significant decline in cholesterol biosynthesis, encompassed by the gene set linked to cluster 1. Finally, we note less expression following PMA treatment with *CERS5*/*6* KD samples compared to controls in cluster 4, which is representative of ECM organization and remodeling. Overall, the hypertrophy response changes due to *CERS5*/*6* KD are compelling in that the hypertrophy treatment appeared to be less severe in samples with *CERS5*/*6* KD compared to controls.

A limitation of this study stems from the fact that immortalized human ventricular cardiomyocytes were used as opposed to primary cells, as the responses to stimuli may not be fully recapitulated in immortalized cardiomyocyte biology. It is worth mentioning that a pseudo-primary cardiomyocyte cell line was initially utilized in early phases of this study, yet the immortalized cell line was ultimately selected for our continued evaluation for a few reasons. First, we deemed these cells the most biologically relevant model due to the more physiologically relevant expression of cardiac markers: *GATA4*, *ACTB*, *SMAD2*, *VEGFB*, *NPPB*, *CAMK2D*, and *CLCN3*. However, immunofluorescence-based confirmation (e.g., PCM-1 staining) was not performed in this study. Second, the baseline expression of *NPPB* allowed us to use this clinical biomarker to indicate hypertrophic cells and perform a direct comparison between experimental groups. It is also worth mentioning that while PMA-induced hypertrophy was selected as our model for HF, it may not entirely capture the pathophysiology and complexities of human cardiovascular disease. However, this work provides important data supporting the different roles both LC and VLC ceramides may play in human heart disease.

Although the data presented here support the conclusion that *CERS5*/*6* KD leads to protection against the severity of PMA treatment, we observed an appreciable number of changes due to *CERS5*/*6* KD alone [15]. It is possible that inhibition of any single *CERS* could result in increased toxicity and cell death based on the role the ceramides play in regulated cell death, like apoptosis, as demonstrated by exposure to the mycotoxin fumonisin, a potent CERS inhibitor [33,34]. For this reason, we think a future therapeutic approach might be to increase the concentration of VLC ceramide species by developing a compound that either induces or activates *CERS2*, rather than inhibiting CERS5/6. Additionally, previous work suggests that diet has an impact on ceramide levels and species [35]. Walker et al. investigated the impact of the Mediterranean-style diet on 24:0/16:0 ceramide levels, and suggested that the 24:0/16:0 ceramide ratio was inversely associated with CVD and cancer mortality, while 24:0/16:0 ceramide ratios with cancer mortality were attenuated among participants with a Mediterranean-style diet or other higher quality diet [36]. This work suggests that it could also be possible to regulate ceramide levels through diet, as opposed to a pharmaceutical option. This work is important as it presents the first examination of the role specific ceramide synthases play in the development of cardiac hypertrophy. Understanding the physiology of the different ceramide species is crucial to developing therapeutic agents targeting modifiable pathways in the sphingolipid metabolic scheme.

## 5. Conclusions

In conclusion, contrasting changes are observed in the hypertrophic response with *CERS2* KD or *CERS5*/*6* KD. HCMs with silenced *CERS2,* resulting in reduced VLC ceramide cellular content, and then subjected to hypertrophy-inducing conditions, display increases in HCM remodeling, cholesterol biosynthesis, and lipid metabolism, and decreases in the immune response, indicative of more advanced CVD progression. While following *CERS5*/*6* KD, HCMs with reduced 16:0 ceramide and the corresponding sphingolipid products underwent pathway changes, suggesting an increase in the immune response and a decline in HCM remodeling, cholesterol biosynthesis, and lipid metabolism, reflective of a more favorable hypertrophic response following PMA treatment and potentially healthier cells. These findings support the hypothesis that *CERS2* and VLC ceramides may play a protective role against CVD progression, while *CERS5*/*6* and 16:0 ceramide could have a harmful effect and contribute to the development and progression of CVD. These findings emphasize the importance of research that will characterize the role that VLC and LC sphingolipid species play in both the development and progression of cardiovascular disease.

## Data Availability

The RNA sequencing datasets generated and analyzed during the current study meeting MINSEQE (Minimum Information About a Next-generation Sequencing Experiment) guidelines are available in the GEO repository, https://www.ncbi.nlm.nih.gov/geo/query/acc.cgi?acc=GSE285142 (accessed on 27 August 2025). Additional datasets generated and analyzed during the current study are available from the corresponding author on reasonable request.

## Supplementary Materials

The following supporting information can be downloaded at: https://www.mdpi.com/article/10.3390/metabo15090635/s1, Supplementary Table S1. List of differentially altered gene sets due to 48 h PMA treatment (FDR < 0.05). Supplementary Table S2. List of the genes identified as different due to both *CERS2* KD and PMA treatment, with their respective z-score and cluster. Supplementary Table S3. Full list of changed pathways due to *CERS2* KD and PMA treatment using the entire LRT gene list with the corresponding clusters labeled (FDR < 0.05). Supplementary Table S4. Complete gene list identified as different due to both *CERS5*/*6* KD and PMA treatment, with their respective z-score and cluster. Supplementary Table S5. Complete list of pathways changed due to *CERS5*/*6* KD and PMA treatment using the entire LRT gene list with the corresponding clusters labeled (FDR < 0.05). Supplemental raw and normalized RNA-sequencing counts can be found in the CERS KD Supplemental Counts file.

## Abbreviations

The following abbreviations are used in this manuscript:

CVD	cardiovascular disease
LDL	low density lipoprotein
CerS	ceramide synthase
HF	heart failure
SM	sphingomyelin
LC	long-chain
VLC	very long-chain
HCM	human ventricular cardiomyocytes
KD	knockdown
PMA	phorbol 12-myristate 13-acetate
GSEA	gene set enrichment analysis
FDR	false discovery rate
LRT	likelihood ratio test
BNP	B-type natriuretic peptide
NPPB	gene name for BNP
PCA	principal component analysis
DEGs	differentially expressed genes
ECM	extracellular matrix
LDLR	low density lipoprotein receptor
ACTA2	actin alpha 2
PLIN2	perilipin 2
ANKRD1	ankyrin repeat domain 1
PKC	protein kinase C
OXPHOS	oxidative phosphorylation
TCA	tricarboxylic acid cycle
