# Supplementary material for: Deciphering the Role of Different Ceramide Synthases in the Human Cardiomyocyte Hypertrophic Response
Source: Metabolites. 2025 Sep 22;15(9):635. doi: 10.3390/metabo15090635 (PMC12471689; doi:10.3390/metabo15090635)
Supplement: Supplementary file 1 [file metabolites-15-00635-s001.zip › metabolites-3774649-supplementary/supplementary/metabolites-3774649-supplementary (1).pdf]

Supplementary Table S1. List of differentially altered gene sets due to 48h PMA treatment (FDR < 0.05).

Gene Set Name	Normalized Enrichment Score (NES)	FDR
HALLMARK_HYPOXIA	4.60	0.00E+00
HALLMARK_TNFA_SIGNALING_VIA_NFKB	4.39	0.00E+00
REACTOME_TRANSPORT_OF_SMALL_MOLECULES	4.33	0.00E+00
REACTOME_SLC_MEDIATED_TRANSMEMBRANE_TRANSPORT	4.32	0.00E+00
WP_PHOTODYNAMIC_THERAPY_INDUCED_UNFOLDED_PROTEIN_RESPONSE	4.18	0.00E+00
REACTOME_UNFOLDED_PROTEIN_RESPONSE_UPR	4.16	0.00E+00
REACTOME_RESPONSE_TO_ELEVATED_PLATELET_CYTOSOLIC_CA2	3.90	0.00E+00
HALLMARK_INTERFERON_ALPHA_RESPONSE	3.87	0.00E+00
HALLMARK_MTORC1_SIGNALING	3.79	0.00E+00
KEGG_LYSOSOME	3.76	0.00E+00
HALLMARK_INTERFERON_GAMMA_RESPONSE	3.74	0.00E+00
HALLMARK_EPITHELIAL_MESENCHYMAL_TRANSITION	3.64	0.00E+00
WP_NUCLEAR_RECEPTORS_META_PATHWAY	3.60	0.00E+00
REACTOME_IRE1ALPHA_ACTIVATES_CHAPERONES	3.57	0.00E+00
REACTOME_ASPARAGINE_N_LINKED_GLYCOSYLATION	3.57	0.00E+00
REACTOME_ION_CHANNEL_TRANSPORT	3.53	0.00E+00
HALLMARK_CHOLESTEROL_HOMEOSTASIS	3.49	0.00E+00
REACTOME_AMINO_ACID_TRANSPORT_ACROSS_THE_PLASMA_MEMBRANE	3.45	0.00E+00
REACTOME_ANTIGEN_PRESENTATION_FOLDING_ASSEMBLY_AND_PEPTIDE_LOADING_OF_CLASS_I_MHC	3.44	0.00E+00
REACTOME_TRANSPORT_OF_INORGANIC_CATIONS_ANIONS_AND_AMINO_ACIDS_OLIGOPEPTIDES	3.44	0.00E+00
WP_LUNG_FIBROSIS	3.41	0.00E+00
REACTOME_INTERLEUKIN_4_AND_INTERLEUKIN_13_SIGNALING	3.39	0.00E+00
REACTOME_EXTRACELLULAR_MATRIX_ORGANIZATION	3.38	0.00E+00
WP_PROXIMAL_TUBULE_TRANSPORT	3.35	0.00E+00
HALLMARK_IL2_STAT5_SIGNALING	3.34	0.00E+00
HALLMARK_INFLAMMATORY_RESPONSE	3.34	0.00E+00
KEGG_MEDICUS_REFERENCE_ANTIGEN_PROCESSING_AND_PRESENTATION_BY_MHC_CLASS_I_MOLECULES	3.33	0.00E+00
HALLMARK_ANDROGEN_RESPONSE	3.33	0.00E+00
HALLMARK_UNFOLDED_PROTEIN_RESPONSE	3.32	0.00E+00

KEGG_CYTOKINE_CYTOKINE_RECEPTOR_INTERACTION	3.23	0.00E+00
WP_PLEURAL_MESOTHELIOMA	3.21	0.00E+00
REACTOME_NEUTROPHIL_DEGRANULATION	3.21	0.00E+00
WP_PHOTODYNAMIC_THERAPY_INDUCED_NF_KB_SURVIVAL_SIGNALING	3.11	0.00E+00
REACTOME_REGULATION_OF_INSULIN_LIKE_GROWTH_FACTOR_IGF_TRANSPORT_AND_UPTAKE_BY_INSULIN_LIKE_GROWTH_FACTOR_BINDING_PROTEINS_IGFBPS	3.10	0.00E+00
REACTOME_DISEASES_OF_GLYCOSYLATION	3.03	0.00E+00
REACTOME_ION_TRANSPORT_BY_P_TYPE_ATPASES	3.00	0.00E+00
WP_VEGFA_VEGFR2_SIGNALING	2.98	0.00E+00
WP_CHOLESTEROL_METABOLISM_WITH_BLOCH_AND_KANDUTSCH_RUSSELL_PATHWAYS	2.97	0.00E+00
WP_ADIPOGENESIS	2.95	0.00E+00
WP_COPPER_HOMEOSTASIS	2.94	0.00E+00
REACTOME_INTERFERON_ALPHA_BETA_SIGNALING	2.91	0.00E+00
HALLMARK_PROTEIN_SECRETION	2.90	0.00E+00
WP_SPHINGOLIPID_METABOLISM_INTEGRATED_PATHWAY	2.89	0.00E+00
WP_ZINC_HOMEOSTASIS	2.88	0.00E+00
WP_NETWORK_MAP_OF_SARS_COV_2_SIGNALING_PATHWAY	2.88	0.00E+00
REACTOME_METALLOTHIONEINS_BIND_METALS	2.85	0.00E+00
REACTOME_SPHINGOLIPID_METABOLISM	2.82	0.00E+00
WP_PLURIPOTENT_STEM_CELL_DIFFERENTIATION_PATHWAY	2.81	0.00E+00
WP_NEUROINFLAMMATION_AND_GLUTAMATERGIC_SIGNALING	2.80	0.00E+00
REACTOME_INTERLEUKIN_10_SIGNALING	2.80	0.00E+00
REACTOME_DISEASES_OF_METABOLISM	2.79	0.00E+00
WP_GLUCOCORTICOID_RECEPTOR_PATHWAY	2.78	0.00E+00
WP_AUTOSOMAL_RECESSIVE_OSTEOPETROSIS_PATHWAYS	2.78	0.00E+00
KEGG_STEROID_BIOSYNTHESIS	2.76	0.00E+00
KEGG_BLADDER_CANCER	2.74	0.00E+00
HALLMARK_MYOGENESIS	2.73	1.00E-03
REACTOME_PLATELET_ACTIVATION_SIGNALING_AND_AGGREGATION	2.73	1.00E-03
KEGG_MEDICUS_PATHOGEN_HTLV_1_P12_TO_ANTIGEN_PROCESSING_AND_PRESENTATION_BY_MHC_CLASS_I_MOLECULES	2.73	1.00E-03
REACTOME_HEMOSTASIS	2.66	1.00E-03
WP_VITAMIN_B12_METABOLISM	2.66	1.00E-03
KEGG_PROTEIN_EXPORT	2.64	1.00E-03
WP_OVERVIEW_OF_PROINFLAMMATORY_AND_PROFIBROTIC_MEDIATORS	2.63	1.00E-03

REACTOME_IMMUNOREGULATORY_INTERACTIONS_BETWEEN_A_LYMPHOID_AND_A_NON_LYMPHOID_CELL	2.63	1.00E-03
REACTOME_RESPONSE_OF_EIF2AK1_HRI_TO_HEME_DEFICIENCY	2.63	1.00E-03
HALLMARK_APOPTOSIS	2.62	1.00E-03
KEGG_MEDICUS_PATHOGEN_HPV_E5_TO_ANTIGEN_PROCESSING_AND_PRESENTATION_BY_MHC_CLASS_I_MOLECULES	2.62	1.00E-03
REACTOME_CALNEXIN_CALRETICULIN_CYCLE	2.62	1.00E-03
REACTOME_METABOLISM_OF_STEROIDS	2.60	1.00E-03
KEGG_MEDICUS_REFERENCE_N_GLYCAN_PRECURSOR_BIOSYNTHESIS_ALG6_TO_OST	2.59	1.00E-03
REACTOME_ENDOSOMAL_VACUOLAR_PATHWAY	2.59	1.00E-03
HALLMARK_ESTROGEN_RESPONSE_EARLY	2.58	1.00E-03
REACTOME_REGULATED_PROTEOLYSIS_OF_P75NTR	2.58	1.00E-03
KEGG_MEDICUS_REFERENCE_NOTCH_PROTEOLYTIC_ACTIVATION	2.58	2.00E-03
WP_UNFOLDED_PROTEIN_RESPONSE	2.55	2.00E-03
HALLMARK_HEME_METABOLISM	2.54	2.00E-03
WP_SPHINGOLIPID_METABOLISM_IN_SENESCENCE	2.54	2.00E-03
WP_CHOLESTEROL_METABOLISM	2.53	2.00E-03
REACTOME_CHOLESTEROL_BIOSYNTHESIS	2.52	3.00E-03
KEGG_MEDICUS_REFERENCE_CHOLESTEROL_BIOSYNTHESIS	2.51	3.00E-03
REACTOME_TRP_CHANNELS	2.50	3.00E-03
WP_CHOLESTEROL_BIOSYNTHESIS_PATHWAY_IN_HEPATOCYTES	2.47	4.00E-03
KEGG_VIBRIO_CHOLERAE_INFECTION	2.46	4.00E-03
REACTOME_METABOLISM_OF_STEROID_HORMONES	2.46	4.00E-03
HALLMARK_P53_PATHWAY	2.46	4.00E-03
WP_PROSTAGLANDIN_SIGNALING	2.45	4.00E-03
PID_ATF2_PATHWAY	2.45	4.00E-03
REACTOME_ATF6_ATF6_ALPHA_ACTIVATES_CHAPERONE_GENES	2.44	4.00E-03
REACTOME_GLYCOSPHINGOLIPID_METABOLISM	2.44	4.00E-03
WP_DEGRADATION_PATHWAY_OF_SPHINGOLIPIDS_INCLUDING_DISEASES	2.44	4.00E-03
REACTOME_TRANSFERRIN_ENDOCYTOSIS_AND_RECYCLING	2.44	4.00E-03
KEGG_MEDICUS_PATHOGEN_KSHV_MIR1_2_TO_ANTIGEN_PROCESSING_AND_PRESENTATION_BY_MHC_CLASS_I_MOLECULES	2.44	4.00E-03
REACTOME_CYTOCHROME_P450_ARRANGED_BY_SUBSTRATE_TYPE	2.42	4.00E-03
REACTOME_TRANSPORT_OF_BILE_SALTS_AND_ORGANIC_ACIDS_METAL_IONS_AND_AMINE_COMPOUNDS	2.41	5.00E-03
WP_OXIDATION_BY_CYTOCHROME_P450	2.41	5.00E-03
WP_CHOLESTEROL_SYNTHESIS_DISORDERS	2.40	5.00E-03

WP_SPHINGOLIPID_METABOLISM_OVERVIEW	2.40	5.00E-03
KEGG_GLYCOSAMINOGLYCAN_DEGRADATION	2.40	5.00E-03
REACTOME_INSULIN_RECEPTOR_RECYCLING	2.39	5.00E-03
REACTOME_N_GLYCAN_TRIMMING_IN_THE_ER_AND_CALNEXIN_CALRETICULIN_CYCLE	2.38	6.00E-03
WP_MRNA_PROTEIN_AND_METABOLITE_INDUCATION_PATHWAY_BY_CYCLOSPORIN_A	2.36	6.00E-03
KEGG_MEDICUS_REFERENCE_GF_RTK_PI3K_SIGNALING_PATHWAY	2.36	6.00E-03
WP_ENTEROCYTE_CHOLESTEROL_METABOLISM	2.36	6.00E-03
WP_MONOAMINE_GPCRS	2.36	6.00E-03
REACTOME_CARGO_CONCENTRATION_IN_THE_ER	2.35	7.00E-03
REACTOME_METABOLISM_OF_ANGIOTENSINOGEN_TO_ANGIOTENSINS	2.35	7.00E-03
WP_SYNTHESIS_OF_CERAMIDES_AND_1_DEOXYCERAMIDES	2.34	7.00E-03
KEGG_MEDICUS_REFERENCE_AHR_SIGNALING_PATHWAY	2.34	7.00E-03
WP_FERROPTOSIS	2.33	8.00E-03
REACTOME_MATURATION_OF_SARS_COV_2_SPIKE_PROTEIN	2.32	8.00E-03
WP_CLOCK_CONTROLLED_AUTOPHAGY_IN_BONE_METABOLISM	2.32	8.00E-03
REACTOME_INTERFERON_GAMMA_SIGNALING	2.32	8.00E-03
KEGG_MEDICUS_REFERENCE_COPI_VESICLE_FORMATION	2.31	8.00E-03
WP_SELENIUM_MICRONUTRIENT_NETWORK	2.31	8.00E-03
HALLMARK_ESTROGEN_RESPONSE_LATE	2.31	8.00E-03
HALLMARK_UV_RESPONSE_DN	2.30	9.00E-03
REACTOME_PLATELET_CALCIUM_HOMEOSTASIS	2.30	9.00E-03
REACTOME_RESPONSE_TO_METAL_IONS	2.29	9.00E-03
KEGG_HEMATOPOIETIC_CELL_LINEAGE	2.28	1.00E-02
WP_CHOLESTEROL_BIOSYNTHESIS_PATHWAY	2.27	1.00E-02
REACTOME_SPHINGOLIPID_DE_NOVO_BIOSYNTHESIS	2.26	1.10E-02
WP_NRF2_PATHWAY	2.25	1.20E-02
BIOCARTA_GRANULOCYTES_PATHWAY	2.25	1.20E-02
REACTOME_SIGNALING_BY_INSULIN_RECEPTOR	2.24	1.20E-02
WP_GLYCOSYLATION_AND_RELATED_CONGENITAL_DEFECTS	2.24	1.20E-02
KEGG_RENIN_ANGIOTENSIN_SYSTEM	2.23	1.30E-02
WP_N_GLYCAN_BIOSYNTHESIS	2.23	1.30E-02
REACTOME_PEPTIDE_HORMONE_METABOLISM	2.23	1.30E-02
BIOCARTA_IL17_PATHWAY	2.22	1.40E-02
WP_HEMATOPOIETIC_STEM_CELL_DIFFERENTIATION	2.21	1.50E-02
KEGG_PATHWAYS_IN_CANCER	2.21	1.50E-02
WP_PI3K_AKT_SIGNALING_PATHWAY	2.20	1.60E-02

REACTOME_ATF6_ATF6_ALPHA_ACTIVATES_CHAPERONES	2.20	1.60E-02
WP_BLADDER_CANCER	2.20	1.60E-02
WP_COVID_19_ADVERSE_OUTCOME_PATHWAY	2.19	1.70E-02
WP_FOCAL_ADHESION_PI3K_AKT_MTOR_SIGNALING_PATHWAY	2.18	1.70E-02
HALLMARK_XENOBIOTIC_METABOLISM	2.18	1.70E-02
KEGG_MEDICUS_REFERENCE_TETHERING_OF_LATE_ENDOSOMES_AND_LYSOSOMES	2.18	1.70E-02
REACTOME_GPCR_LIGAND_BINDING	2.18	1.80E-02
WP_OSTEOBLAST_SIGNALING	2.17	1.80E-02
KEGG_SPHINGOLIPID_METABOLISM	2.17	1.80E-02
PID_VEGF_VEGFR_PATHWAY	2.17	1.80E-02
WP_EBOLA_VIRUS_INFECTION_IN_HOST	2.17	1.80E-02
REACTOME_DISEASES_ASSOCIATED_WITH_GLYCOSYLATION_PRECURSOR_BIOSYNTHESIS	2.17	1.80E-02
WP_FOLATE_METABOLISM	2.16	1.80E-02
REACTOME_BIOSYNTHESIS_OF_THE_N_GLYCAN_PRECURSOR_DOLICHOL_LIPID_LINKED_OLIGOSACCHARIDE_LLO_AND_TRANSFER_TO_A_NASCENT_PROTEIN	2.16	1.90E-02
WP_IMMUNE_INFILTRATION_IN_PANCREATIC_CANCER	2.15	1.90E-02
WP_AMINO_ACID_METABOLISM_IN_TRIPLE_NEGATIVE_BREAST_CANCER_CELLS	2.15	1.90E-02
REACTOME_COPII_MEDIATED_VESICLE_TRANSPORT	2.15	2.00E-02
REACTOME_KERATAN_SULFATE_DEGRADATION	2.15	2.00E-02
WP_CELLS_AND_MOLECULES_INVOLVED_IN_LOCAL_ACUTE_INFLAMMATORY_RESPONSE	2.14	2.00E-02
WP_CELL_TYPE_DEPENDENT_SELECTIVITY_OF_CCK2R_SIGNALING	2.14	2.00E-02
KEGG_MEDICUS_REFERENCE_ITGA_B_TALIN_VINCULIN_SIGNALING_PATHWAY	2.14	2.00E-02
PID_AP1_PATHWAY	2.14	2.10E-02
KEGG_MEDICUS_PATHOGEN_HPV_E7_TO_CELL_CYCLE_G1_S	2.14	2.10E-02
REACTOME_AMINE_LIGAND_BINDING_RECEPTORS	2.13	2.10E-02
WP_TYPE_I_INTERFERON_INDUCTION_AND_SIGNALING_DURING_SARS_COV_2_INFECTION	2.13	2.10E-02
REACTOME_GLYCOSPHINGOLIPID_CATABOLISM	2.13	2.10E-02
WP_SPINAL_CORD_INJURY	2.12	2.20E-02
KEGG_MEDICUS_REFERENCE_GF_RTK_RAS_PI3K_SIGNALING_PATHWAY	2.12	2.20E-02
PID_FRA_PATHWAY	2.12	2.20E-02
REACTOME_ERYTHROCYTES_TAKE_UP_CARBON_DIOXIDE_AND_RELEASE_OXYGEN	2.12	2.30E-02
KEGG_MEDICUS_REFERENCE_GF_RTK_RAS_ERK_SIGNALING_PATHWAY	2.11	2.30E-02
WP_RAS_AND_BRADYKININ_PATHWAYS_IN_COVID_19	2.11	2.30E-02
WP_GLYCEROPHOSPHOLIPID_BIOSYNTHETIC_PATHWAY	2.11	2.30E-02

BIOCARTA_LAIR_PATHWAY	2.11	2.30E-02
REACTOME_PRE_NOTCH_PROCESSING_IN_GOLGI	2.11	2.30E-02
KEGG_MEDICUS_PATHOGEN_SARS_COV_2_S_TO_ANGII_AT1R_NOX2_SIGNALING_PATHWAY	2.11	2.30E-02
KEGG_CIRCADIAN_RHYTHM_MAMMAL	2.10	2.50E-02
HALLMARK_COMPLEMENT	2.09	2.60E-02
REACTOME_O_GLYCOSYLATION_OF_TSR_DOMAIN_CONTAINING_PROTEINS	2.09	2.60E-02
WP_PROSTAGLANDIN_AND_LEUKOTRIENE_METABOLISM_IN_SENESCENCE_WP5321	2.08	2.70E-02
KEGG_ARRHYTHMOGENIC_RIGHT_VENTRICULAR_CARDIOMYOPATHY_ARVC	2.08	2.70E-02
WP_INCLUSION_BODY_MYOSITIS	2.08	2.70E-02
REACTOME_BUTYROPHILIN_BTN_FAMILY_INTERACTIONS	2.08	2.70E-02
BIOCARTA_ERYTH_PATHWAY	2.08	2.70E-02
KEGG_MEDICUS_VARIANT_AMPLIFIED_CCND1_TO_CELL_CYCLE_G1_S	2.07	2.80E-02
KEGG_ALDOSTERONE_REGULATED_SODIUM_REABSORPTION	2.07	2.80E-02
WP_ARRHYTHMOGENIC_RIGHT_VENTRICULAR_CARDIOMYOPATHY	2.07	2.80E-02
REACTOME_RUNX3_REGULATES_P14_ARF	2.07	2.80E-02
KEGG_MEDICUS_VARIANT_SCRAPIE_CONFORMATION_PRPSC_TO_PERK_ATF4_SIGNALING_PATHWAY	2.07	2.80E-02
REACTOME_ENDOGENOUS_STEROLS	2.07	2.80E-02
REACTOME_IRON_UPTAKE_AND_TRANSPORT	2.07	2.80E-02
WP_GLYCOSAMINOGLYCAN_DEGRADATION	2.06	2.80E-02
KEGG_MEDICUS_REFERENCE_ITGA_B_RHOGEF_RHOA_SIGNALING_PATHWAY	2.06	2.80E-02
WP_EXTRAFOLLICULAR_AND_FOLLICULAR_B_CELL_ACTIVATION_BY_SARS_COV_2	2.06	2.80E-02
HALLMARK_KRAS_SIGNALING_UP	2.06	2.80E-02
WP_EPITHELIAL_TO_MESENCHYMAL_TRANSITION_IN_COLORECTAL_CANCER	2.06	2.80E-02
WP_INTERACTIONS_OF_NATURAL_KILLER_CELLS_IN_PANCREATIC_CANCER	2.06	2.90E-02
BIOCARTA_STEM_PATHWAY	2.06	2.90E-02
BIOCARTA_EICOSANOID_PATHWAY	2.05	2.90E-02
KEGG_STEROID_HORMONE_BIOSYNTHESIS	2.05	2.90E-02
HALLMARK_ALLOGRAFT_REJECTION	2.05	2.90E-02
HALLMARK_COAGULATION	2.05	2.90E-02
KEGG_DORSO_VENTRAL_AXIS_FORMATION	2.05	3.00E-02
KEGG_N_GLYCAN_BIOSYNTHESIS	2.04	3.00E-02
REACTOME_CLASS_A_1_RHODOPSIN_LIKE_RECEPTORS	2.04	3.00E-02
REACTOME_METAL_ION_SLC_TRANSPORTERS	2.04	3.00E-02
BIOCARTA_VITCB_PATHWAY	2.04	3.10E-02

KEGG_MEDICUS_REFERENCE_C9ORF72_MEDIATED_AUTOPHAGY_INITIATION	2.03	3.10E-02
KEGG_GRAFT_VERSUS_HOST_DISEASE	2.03	3.30E-02
WP_CHOLESTEROL_BIOSYNTHESIS_WITH_SKELETAL_DYSPLASIAS	2.02	3.50E-02
HALLMARK_TGF_BETA_SIGNALING	2.02	3.50E-02
WP_ARYL_HYDROCARBON_RECEPTOR_PATHWAY_WP2586	2.01	3.60E-02
REACTOME_PHOSPHOLIPID_METABOLISM	2.01	3.60E-02
WP_KENNEDY_PATHWAY_FROM_SPHINGOLIPIDS	2.01	3.60E-02
KEGG_CELL_ADHESION_MOLECULES_CAMS	2.01	3.60E-02
REACTOME_PERK_REGULATES_GENE_EXPRESSION	2.00	3.70E-02
REACTOME_CS_DS_DEGRADATION	2.00	3.70E-02
BIOCARTA_INFLAM_PATHWAY	2.00	3.70E-02
KEGG_MEDICUS_VARIANT_MUTATION_ACTIVATED_PRKACA_TO_ACTH_CORTISOL_SIGNALING_PATHWAY	2.00	3.80E-02
REACTOME_VEGF_LIGAND_RECEPTOR_INTERACTIONS	1.99	3.80E-02
WP_TGF_BETA_RECEPTOR_SIGNALING_IN_SKELETAL_DYSPLASIAS	1.99	3.80E-02
REACTOME_SERINE_BIOSYNTHESIS	1.99	3.80E-02
WP_DISTURBED_PATHWAYS_IN_DUCHENNE_MUSCULAR_DYSTROPHY	1.99	3.80E-02
WP_H19_ACTION_RB_E2F1_SIGNALING_AND_CDK_BETA_CATENIN_ACTIVITY	1.99	3.80E-02
REACTOME_DEGRADATION_OF_THE_EXTRACELLULAR_MATRIX	1.99	3.80E-02
WP_CYTOKINES_AND_INFLAMMATORY_RESPONSE	1.99	3.80E-02
PID_INTEGRIN_CS_PATHWAY	1.99	3.80E-02
KEGG_MEDICUS_REFERENCE_ITGA_B_RHOGAP_RHOA_SIGNALING_PATHWAY	1.99	3.80E-02
REACTOME_DISEASES_ASSOCIATED_WITH_O_GLYCOSYLATION_OF_PROTEINS	1.98	3.90E-02
WP_EMBRYONIC_STEM_CELL_PLURIPOTENCY_PATHWAYS	1.98	3.90E-02
REACTOME_MISCELLANEOUS_TRANSPORT_AND_BINDING_EVENTS	1.98	3.90E-02
REACTOME_ER_QUALITY_CONTROL_COMPARTMENT_ERQC	1.98	4.00E-02
REACTOME_CALCITONIN_LIKE_LIGAND_RECEPTORS	1.98	4.00E-02
REACTOME_MUCOPOLYSACCHARIDOSES	1.97	4.00E-02
WP_MAPK_SIGNALING_PATHWAY	1.97	4.00E-02
WP_FAMILIAL_HYPERLIPIDEMIA_TYPE_1	1.97	4.20E-02
KEGG_MEDICUS_REFERENCE_IL6_FAMILY_TO_JAK_STAT_SIGNALING_PATHWAY	1.96	4.20E-02
WP_HAIR_FOLLICLE_DEVELOPMENT_CYTODIFFERENTIATION_PART_3_OF_3	1.95	4.50E-02
KEGG_MEDICUS_REFERENCE_AUTOPHAGOSOME_AND_LYSOSOME_FUSION_TETHERING_FACTOR	1.95	4.50E-02
KEGG_HYPERTROPHIC_CARDIOMYOPATHY_HCM	1.95	4.50E-02

WP_GPCRS_CLASS_C_METABOTROPIC_GLUTAMATE_PHEROMONE	1.95	4.50E-02
REACTOME_POST_TRANSLATIONAL_MODIFICATION_SYNTHESIS_OF_GPI_ANCHORED_PROTEINS	1.95	4.50E-02
KEGG_MEDICUS_PATHOGEN_SALMONELLA_SIFA_TO_TETHERING_OF_LATE_ENDOSOMES_AND_LYSOSOMES	1.95	4.50E-02
KEGG_MEDICUS_REFERENCE_ITGA_B_FAK_CDC42_SIGNALING_PATHWAY	1.95	4.60E-02
KEGG_AMINO_SUGAR_AND_NUCLEOTIDE_SUGAR_METABOLISM	1.94	4.60E-02
WP_MELATONIN_METABOLISM_AND_EFFECTS	1.94	4.60E-02
WP_MIRNA_ROLE_IN_IMMUNE_RESPONSE_IN_SEPSIS	1.94	4.70E-02
WP_UDP_DERIVED_SUGARS_SYNTHESIS_IN_FIBROBLASTS	1.94	4.70E-02
REACTOME_TRANSLATION_OF_SARS_COV_2_STRUCTURAL_PROTEINS	1.94	4.70E-02
REACTOME_SYNTHESIS_OF_VERY_LONG_CHAIN_FATTY_ACYL_COAS	1.94	4.70E-02
WP_LDL_INFLUENCE_ON_CD14_AND_TLR4	1.93	4.90E-02
HALLMARK_MYC_TARGETS_V1	-9.02	0.00E+00
REACTOME_RRNA_PROCESSING	-8.57	0.00E+00
REACTOME_PROCESSING_OF_CAPPED_INTRON_CONTAINING_PRE_MRNA	-8.18	0.00E+00
REACTOME_TRANSLATION	-7.72	0.00E+00
REACTOME_MRNA_SPLICING	-7.43	0.00E+00
REACTOME_REGULATION_OF_EXPRESSION_OF_SLITS_AND_ROBOS	-7.11	0.00E+00
REACTOME_SIGNALING_BY_ROBO_RECEPTORS	-6.83	0.00E+00
KEGG_SPLICEOSOME	-6.27	0.00E+00
REACTOME_CELL_CYCLE_MITOTIC	-6.27	0.00E+00
HALLMARK_E2F_TARGETS	-6.17	0.00E+00
REACTOME_INFLUENZA_INFECTION	-6.16	0.00E+00
REACTOME_EUKARYOTIC_TRANSLATION_INITIATION	-5.96	0.00E+00
WP_CYTOPLASMIC_RIBOSOMAL_PROTEINS	-5.96	0.00E+00
KEGG_MEDICUS_REFERENCE_TRANSLATION_INITIATION	-5.90	0.00E+00
REACTOME_EUKARYOTIC_TRANSLATION_ELONGATION	-5.87	0.00E+00
KEGG_RIBOSOME	-5.81	0.00E+00
WP_MRNA_PROCESSING	-5.78	0.00E+00
REACTOME_METABOLISM_OF_AMINO_ACIDS_AND_DERIVATIVES	-5.71	0.00E+00
KEGG_HUNTINGTONS_DISEASE	-5.62	0.00E+00
REACTOME_NONSENSE_MEDIATED_DECAY_NMD	-5.62	0.00E+00
REACTOME_M_PHASE	-5.55	0.00E+00
REACTOME_NERVOUS_SYSTEM_DEVELOPMENT	-5.52	0.00E+00
REACTOME_MITOTIC_METAPHASE_AND_ANAPHASE	-5.47	0.00E+00
REACTOME_DNA_REPLICATION	-5.40	0.00E+00

REACTOME_MITOCHONDRIAL_TRANSLATION	-5.38	0.00E+00
REACTOME_SEPARATION_OF_SISTER_CHROMATIDS	-5.36	0.00E+00
HALLMARK_OXIDATIVE_PHOSPHORYLATION	-5.32	0.00E+00
REACTOME_SYNTHESIS_OF_DNA	-5.31	0.00E+00
REACTOME_SELENOAMINO_ACID_METABOLISM	-5.27	0.00E+00
REACTOME_MITOTIC_G2_G2_M_PHASES	-5.26	0.00E+00
KEGG_PARKINSONS_DISEASE	-5.15	0.00E+00
REACTOME_HIV_INFECTION	-5.13	0.00E+00
HALLMARK_G2M_CHECKPOINT	-5.11	0.00E+00
KEGG_MEDICUS_VARIANT_MUTATION_INACTIVATED_VCP_TO_26S_PROTEASOME_MEDIATED_PROTEIN_DEGRADATION	-5.10	0.00E+00
REACTOME_CELL_CYCLE_CHECKPOINTS	-5.10	0.00E+00
REACTOME_RESPONSE_OF_EIF2AK4_GCN2_TO_AMINO_ACID_DEFICIENCY	-5.07	0.00E+00
REACTOME_RRNA_MODIFICATION_IN_THE_NUCLEUS_AND_CYTOSOL	-5.07	0.00E+00
KEGG_MEDICUS_VARIANT_SCRAPIE_CONFORMATION_PRPSC_TO_26S_PROTEASOME_MEDIATED_PROTEIN_DEGRADATION	-5.04	0.00E+00
KEGG_MEDICUS_VARIANT_MUTATION_INACTIVATED_UBQLN2_TO_26S_PROTEASOME_MEDIATED_PROTEIN_DEGRADATION	-5.04	0.00E+00
KEGG_MEDICUS_REFERENCE_26S_PROTEASOME_MEDIATED_PROTEIN_DEGRADATION	-5.03	0.00E+00
KEGG_MEDICUS_VARIANT_MUTATION_CAUSED_ABERRANT_SOD1_TO_26S_PROTEASOME_MEDIATED_PROTEIN_DEGRADATION	-5.02	0.00E+00
KEGG_MEDICUS_VARIANT_MUTATION_CAUSED_ABERRANT_ABETA_TO_26S_PROTEASOME_MEDIATED_PROTEIN_DEGRADATION	-5.00	0.00E+00
REACTOME_APC_C_MEDIATED_DEGRADATION_OF_CELL_CYCLE_PROTEINS	-4.95	0.00E+00
REACTOME_SCF_BETA_TRCP_MEDIATED_DEGRADATION_OF_EMI1	-4.94	0.00E+00
REACTOME_THE_ROLE_OF_GTSE1_IN_G2_M_PROGRESSION_AFTER_G2_CHECKPOINT	-4.94	0.00E+00
REACTOME_HOST_INTERACTIONS_OF_HIV_FACTORS	-4.91	0.00E+00
REACTOME_SARS_COV_2_MODULATES_HOST_TRANSLATION_MACHINERY	-4.87	0.00E+00
REACTOME_DNA_REPLICATION_PRE_INITIATION	-4.86	0.00E+00
REACTOME_NEGATIVE_REGULATION_OF_NOTCH4_SIGNALING	-4.86	0.00E+00
KEGG_MEDICUS_VARIANT_MUTATION_CAUSED_ABERRANT_SNCA_TO_26S_PROTEASOME_MEDIATED_PROTEIN_DEGRADATION	-4.85	0.00E+00
REACTOME_S_PHASE	-4.81	0.00E+00

REACTOME_RESPIRATORY_ELECTRON_TRANSPORT_ATP_SYNTHESIS_BY_CHEMIOSMOTIC_COUPLING_AND_HEAT_PRODUCTION_BY_UNCOUPLING_PROTEINS	-4.80	0.00E+00
REACTOME_DEGRADATION_OF_GLI1_BY_THE_PROTEASOME	-4.80	0.00E+00
KEGG_MEDICUS_VARIANT_MUTATION_CAUSED_ABERRANT_HTT_TO_26S_PROTEASOME_MEDIATED_PROTEIN_DEGRADATION	-4.79	0.00E+00
REACTOME_APC_C_CDH1_MEDIATED_DEGRADATION_OF_CDC20_AND_OTHER_APC_C_CDH1_TARGETED_PROTEINS_IN_LATE_MITOSIS_EARLY_G1	-4.78	0.00E+00
REACTOME_RESPIRATORY_ELECTRON_TRANSPORT	-4.71	0.00E+00
REACTOME_METABOLISM_OF_POLYAMINES	-4.69	0.00E+00
REACTOME_THE_CITRIC_ACID_TCA_CYCLE_AND_RESPIRATORY_ELECTRON_TRANSPORT	-4.68	0.00E+00
REACTOME_AUF1_HNRNP_D0_BINDS_AND_DESTABILIZES_MRNA	-4.66	0.00E+00
REACTOME_DECTIN_1_MEDIATED_NONCANONICAL_NF_KB_SIGNALING	-4.66	0.00E+00
REACTOME_REGULATION_OF_MRNA_STABILITY_BY_PROTEINS_THAT_BIND_AU_RICH_ELEMENTS	-4.64	0.00E+00
REACTOME_DNA_REPAIR	-4.61	0.00E+00
WP_ELECTRON_TRANSPORT_CHAIN_OXPHOS_SYSTEM_IN_MITOCHONDRIA	-4.60	0.00E+00
REACTOME_TNFR2_NON_CANONICAL_NF_KB_PATHWAY	-4.58	0.00E+00
KEGG_PROTEASOME	-4.57	0.00E+00
REACTOME_SRP_DEPENDENT_COTRANSLATIONAL_PROTEIN_TARGETING_TO_MEMBRANE	-4.56	0.00E+00
REACTOME_STABILIZATION_OF_P53	-4.55	0.00E+00
REACTOME_REGULATION_OF_RUNX3_EXPRESSION_AND_ACTIVITY	-4.54	0.00E+00
REACTOME_SWITCHING_OF_ORIGINS_TO_A_POST_REPLICATIVE_STATE	-4.53	0.00E+00
REACTOME_DEGRADATION_OF_DVL	-4.51	0.00E+00
REACTOME_NUCLEAR_EVENTS_MEDIATED_BY_NFE2L2	-4.49	0.00E+00
KEGG_MEDICUS_REFERENCE_MITOCHONDRIAL_COMPLEX_UCP1_IN_THERMOGENESIS	-4.48	0.00E+00
REACTOME_DEGRADATION_OF_BETA_CATENIN_BY_THE_DESTRUCTION_COMPLEX	-4.47	0.00E+00
REACTOME_CROSS_PRESENTATION_OF_SOLUBLE_EXOGENOUS_ANTIGENS_ENDOSOMES	-4.43	0.00E+00
REACTOME_NUCLEOTIDE_EXCISION_REPAIR	-4.43	0.00E+00
KEGG_MEDICUS_PATHOGEN_SARS_COV_2_NSP1_TO_TRANSLATION_INITIATION	-4.43	0.00E+00
REACTOME_ORC1_REMOVAL_FROM_CHROMATIN	-4.40	0.00E+00

REACTOME_UCH_PROTEINASES	-4.40	0.00E+00
REACTOME_SARS_COV_1_MODULATES_HOST_TRANSLATION_MACHINERY	-4.38	0.00E+00
REACTOME_SCF_SKP2_MEDIATED_DEGRADATION_OF_P27_P21	-4.36	0.00E+00
REACTOME_DEGRADATION_OF_AXIN	-4.35	0.00E+00
REACTOME_MITOTIC_G1_PHASE_AND_G1_S_TRANSITION	-4.34	0.00E+00
REACTOME_TRNA_PROCESSING	-4.34	0.00E+00
REACTOME_MITOTIC_PROMETAPHASE	-4.34	0.00E+00
REACTOME_REGULATION_OF_RUNX2_EXPRESSION_AND_ACTIVITY	-4.32	0.00E+00
REACTOME_ACTIVATION_OF_THE_MRNA_UPON_BINDING_OF_THE_CAP_BINDING_COMPLEX_AND_EIFS_AND_SUBSEQUENT_BINDING_TO_43S	-4.29	0.00E+00
REACTOME_SOMITOGENESIS	-4.27	0.00E+00
KEGG_ALZHEIMERS_DISEASE	-4.26	0.00E+00
REACTOME_PCP_CE_PATHWAY	-4.26	0.00E+00
REACTOME_ABC_FAMILY_PROTEINS_MEDIATED_TRANSPORT	-4.24	0.00E+00
WP_NONALCOHOLIC_FATTY_LIVER_DISEASE	-4.21	0.00E+00
REACTOME_SIGNALING_BY_NOTCH4	-4.21	0.00E+00
REACTOME_RUNX1_REGULATES_TRANSCRIPTION_OF_GENES_INVOLVED_IN_DIFFERENTIATION_OF_HSCS	-4.19	0.00E+00
REACTOME_DEFECTIVE_CFTR_CAUSES_CYSTIC_FIBROSIS	-4.17	0.00E+00
REACTOME_FORMATION_OF_PARAXIAL_MESODERM	-4.17	0.00E+00
REACTOME_G1_S_DNA_DAMAGE_CHECKPOINTS	-4.16	0.00E+00
REACTOME_TRANSCRIPTION_COUPLED_NUCLEOTIDE_EXCISION_REPAIR_TC_NER	-4.13	0.00E+00
REACTOME_CELLULAR_RESPONSE_TO_CHEMICAL_STRESS	-4.12	0.00E+00
KEGG_OXIDATIVE_PHOSPHORYLATION	-4.11	0.00E+00
REACTOME_KEAP1_NFE2L2_PATHWAY	-4.11	0.00E+00
REACTOME_GLOBAL_GENOME_NUCLEOTIDE_EXCISION_REPAIR_GG_NER	-4.08	0.00E+00
REACTOME_APOPTOSIS	-4.08	0.00E+00
REACTOME_G2_M_CHECKPOINTS	-4.08	0.00E+00
REACTOME_CELLULAR_RESPONSE_TO_STARVATION	-4.07	0.00E+00
REACTOME_PROGRAMMED_CELL_DEATH	-4.06	0.00E+00
REACTOME_RNA_POLYMERASE_II_TRANSCRIPTION_TERMINATION	-4.05	0.00E+00
REACTOME_HEDGEHOG_LIGAND_BIOGENESIS	-4.03	0.00E+00
REACTOME_HEDGEHOG_ON_STATE	-4.01	0.00E+00
REACTOME_FCERI_MEDIATED_NF_KB_ACTIVATION	-4.00	0.00E+00
REACTOME_CLEC7A_DECTIN_1_SIGNALING	-3.98	0.00E+00

REACTOME_TCR_SIGNALING	-3.97	0.00E+00
REACTOME_HEDGEHOG_OFF_STATE	-3.96	0.00E+00
REACTOME_ASYMMETRIC_LOCALIZATION_OF_PCP_PROTEINS	-3.96	0.00E+00
REACTOME_REGULATION_OF_PTEN_STABILITY_AND_ACTIVITY	-3.96	0.00E+00
REACTOME_REGULATION_OF_RAS_BY_GAPS	-3.96	0.00E+00
REACTOME_DEUBIQUITINATION	-3.96	0.00E+00
REACTOME_DOWNSTREAM_SIGNALING_EVENTS_OF_B_CELL_RECEPTOR_BCR	-3.96	0.00E+00
REACTOME_TELOMERE_MAINTENANCE	-3.91	0.00E+00
REACTOME_TRANSCRIPTIONAL_REGULATION_BY_RUNX2	-3.88	0.00E+00
REACTOME_MRNA_SPLICING_MINOR_PATHWAY	-3.87	0.00E+00
KEGG_MEDICUS_VARIANT_MUTATION_CAUSED_ABERRANT_TDP43_TO_ELECTRON_TRANSFER_IN_COMPLEX_I	-3.86	0.00E+00
HALLMARK_MYC_TARGETS_V2	-3.85	0.00E+00
REACTOME_MAPK6_MAPK4_SIGNALING	-3.84	0.00E+00
REACTOME_TRANSPORT_OF_MATURE_TRANSCRIPT_TO_CYTOPLASM	-3.84	0.00E+00
REACTOME_SIGNALING_BY_THE_B_CELL_RECEPTOR_BCR	-3.78	0.00E+00
REACTOME_CELLULAR_RESPONSE_TO_HYPOXIA	-3.77	0.00E+00
REACTOME_C_TYPE_LECTIN_RECEPTORS_CLRS	-3.75	0.00E+00
REACTOME_COMPLEX_I_BIOGENESIS	-3.73	0.00E+00
KEGG_MEDICUS_REFERENCE_ELECTRON_TRANSFER_IN_COMPLEX_I	-3.72	0.00E+00
KEGG_MEDICUS_VARIANT_MUTATION_CAUSED_ABERRANT_SNCA_TO_ELECTRON_TRANSFER_IN_COMPLEX_I	-3.72	0.00E+00
REACTOME_TRANSCRIPTIONAL_REGULATION_BY_RUNX3	-3.71	0.00E+00
REACTOME_ABC_TRANSPORTER_DISORDERS	-3.69	0.00E+00
REACTOME_FC_EPSILON_RECEPTOR_FCERI_SIGNALING	-3.69	0.00E+00
WP_PARKIN_UBIQUITIN_PROTEASOMAL_SYSTEM_PATHWAY	-3.69	0.00E+00
REACTOME_CHROMOSOME_MAINTENANCE	-3.69	0.00E+00
REACTOME_TCF_DEPENDENT_SIGNALING_IN_RESPONSE_TO_WNT	-3.67	0.00E+00
REACTOME_DUAL_INCISION_IN_TC_NER	-3.65	0.00E+00
REACTOME_UB_SPECIFIC_PROCESSING_PROTEASES	-3.65	0.00E+00
REACTOME_BETA_CATENIN_INDEPENDENT_WNT_SIGNALING	-3.64	0.00E+00
REACTOME_CYCLIN_A_CDK2_ASSOCIATED_EVENTS_AT_S_PHASE_ENTRY	-3.63	0.00E+00
REACTOME_INTERLEUKIN_1_SIGNALING	-3.63	0.00E+00
REACTOME_SNRNP_ASSEMBLY	-3.63	0.00E+00
KEGG_MEDICUS_VARIANT_MUTATION_INACTIVATED_PINK1_TO_ELECTRON_TRANSFER_IN_COMPLEX_I	-3.62	0.00E+00
WP_OXIDATIVE_PHOSPHORYLATION	-3.58	0.00E+00
REACTOME_TRANSCRIPTIONAL_REGULATION_BY_RUNX1	-3.56	0.00E+00

REACTOME_GLUCOSE_METABOLISM	-3.56	0.00E+00
REACTOME_RHO_GTPASES_ACTIVATE_FORMINS	-3.54	0.00E+00
WP_MITOCHONDRIAL_COMPLEX_I_ASSEMBLY_MODEL_OXPHOS_SYSTEM	-3.52	0.00E+00
REACTOME_REGULATION_OF_PLK1_ACTIVITY_AT_G2_M_TRANSITION	-3.48	0.00E+00
REACTOME_RESOLUTION_OF_SISTER_CHROMATID_COHESION	-3.47	0.00E+00
REACTOME_MITOTIC_SPINDLE_CHECKPOINT	-3.46	0.00E+00
WP_PROTEASOME_DEGRADATION	-3.44	0.00E+00
REACTOME_SIGNALING_BY_NOTCH	-3.43	0.00E+00
REACTOME_FORMATION_OF_TC_NER_PRE_INCISION_COMPLEX	-3.42	0.00E+00
REACTOME_ORGANELLE_BIOGENESIS_AND_MAINTENANCE	-3.41	0.00E+00
REACTOME_GASTRULATION	-3.39	0.00E+00
KEGG_MEDICUS_REFERENCE_ASSEMBLY_AND_TRAFFICKING_OF_TELOMERASE	-3.39	0.00E+00
REACTOME_EXTENSION_OF_TELOMERES	-3.39	0.00E+00
REACTOME_SIGNALING_BY_HEDGEHOG	-3.38	0.00E+00
REACTOME_NEDDYLATION	-3.38	0.00E+00
REACTOME_COOPERATION_OF_PREFOLDIN_AND_TRIC_CCT_IN_ACTIN_AND_TUBULIN_FOLDING	-3.36	0.00E+00
KEGG_MEDICUS_VARIANT_MUTATION_CAUSED_ABERRANT_ABETA_TO_ELECTRON_TRANSFER_IN_COMPLEX_I	-3.35	0.00E+00
REACTOME_DNA_DOUBLE_STRAND_BREAK_REPAIR	-3.35	0.00E+00
KEGG_GLYCOLYSIS_GLUCONEOGENESIS	-3.35	0.00E+00
REACTOME_SARS_COV_1_HOST_INTERACTIONS	-3.33	0.00E+00
REACTOME_GLYCOLYSIS	-3.32	0.00E+00
HALLMARK_MITOTIC_SPINDLE	-3.30	0.00E+00
REACTOME_SIGNALING_BY_WNT	-3.30	0.00E+00
REACTOME_SARS_COV_2_HOST_INTERACTIONS	-3.29	0.00E+00
REACTOME_ANTIGEN_PROCESSING_UBIQUITINATION_PROTEASOME_DEGRADATION	-3.28	0.00E+00
BIOCARTA_SM_PATHWAY	-3.27	0.00E+00
PID_MYC_ACTIV_PATHWAY	-3.27	0.00E+00
REACTOME_SARS_COV_INFECTIONS	-3.27	0.00E+00
REACTOME_RESOLUTION_OF_AP_SITES_VIA_THE_MULTIPLE_NUCLEOTIDE_PATCH_REPLACEMENT_PATHWAY	-3.26	0.00E+00
REACTOME_RECRUITMENT_OF_MITOTIC_CENTROSOME_PROTEINS_AND_COMPLEXES	-3.25	0.00E+00
REACTOME_INTERLEUKIN_1_FAMILY_SIGNALING	-3.24	0.00E+00
REACTOME_HIV_LIFE_CYCLE	-3.22	0.00E+00
REACTOME_PCNA_DEPENDENT_LONG_PATCH_BASE_EXCISION_REPAIR	-3.21	0.00E+00
REACTOME_PTEN_REGULATION	-3.19	0.00E+00
REACTOME_ANTIGEN_PROCESSING_CROSS_PRESENTATION	-3.19	0.00E+00

REACTOME_TRANSLESION_SYNTHESIS_BY_POLH	-3.19	0.00E+00
REACTOME_RECRUITMENT_OF_NUMA_TO_MITOTIC_CENTROSOMES	-3.19	0.00E+00
WP_NUCLEOTIDE_EXCISION_REPAIR_IN_XERODERMA_PIGMENTOSUM	-3.17	0.00E+00
REACTOME_RHO_GTPASE_EFFECTORS	-3.16	0.00E+00
REACTOME_MITOCHONDRIAL_PROTEIN_IMPORT	-3.16	0.00E+00
REACTOME_POSITIVE_EPIGENETIC_REGULATION_OF_RRNA_EXPRESSION	-3.13	0.00E+00
REACTOME_AURKA_ACTIVATION_BY_TPX2	-3.12	0.00E+00
REACTOME_TRANSCRIPTIONAL_REGULATION_BY_TP53	-3.12	0.00E+00
WP_DNA_MISMATCH_REPAIR	-3.11	0.00E+00
REACTOME_DNA_DAMAGE_RECOGNITION_IN_GG_NER	-3.11	0.00E+00
REACTOME_EPIGENETIC_REGULATION_OF_GENE_EXPRESSION	-3.10	0.00E+00
REACTOME_DUAL_INCISION_IN_GG_NER	-3.08	0.00E+00
WP_RETINOBLASTOMA_GENE_IN_CANCER	-3.07	0.00E+00
WP_METABOLIC_REPROGRAMMING_IN_COLON_CANCER	-3.06	0.00E+00
REACTOME_RESOLUTION_OF_ABASIC_SITES_AP_SITES	-3.05	0.00E+00
REACTOME_RNA_POLYMERASE_I_TRANSCRIPTION	-3.03	0.00E+00
KEGG_MEDICUS_REFERENCE_MISMATCH_REPAIR	-3.03	0.00E+00
BIOCARTA_RANMS_PATHWAY	-3.03	0.00E+00
REACTOME_GAP_FILLING_DNA_REPAIR_SYNTHESIS_AND_LIGATION_IN_GG_NER	-3.02	0.00E+00
REACTOME_FOLDING_OF_ACTIN_BY_CCT_TRIC	-3.01	0.00E+00
REACTOME_SUMOYLATION_OF_DNA_REPLICATION_PROTEINS	-3.01	0.00E+00
REACTOME_ANTIVIRAL_MECHANISM_BY_IFN_STIMULATED_GENES	-3.00	0.00E+00
KEGG_MEDICUS_REFERENCE_GLYCOLYSIS	-2.99	0.00E+00
PID_AURORA_B_PATHWAY	-2.99	0.00E+00
REACTOME_RNA_POLYMERASE_I_TRANSCRIPTION_INITIATION	-2.97	0.00E+00
KEGG_PURINE_METABOLISM	-2.97	0.00E+00
REACTOME_FGFR2_ALTERNATIVE_SPLICING	-2.97	0.00E+00
REACTOME_NUCLEOTIDE_BIOSYNTHESIS	-2.97	0.00E+00
WP_MITOCHONDRIAL_COMPLEX_III_ASSEMBLY	-2.96	0.00E+00
REACTOME_TRNA_MODIFICATION_IN_THE_NUCLEUS_AND_CYTOSOL	-2.96	0.00E+00
KEGG_MEDICUS_REFERENCE_CONDENSIN_LOADING	-2.95	0.00E+00
REACTOME_PROCESSING_OF_CAPPED_INTRONLESS_PRE_MRNA	-2.94	0.00E+00
REACTOME_BASE_EXCISION_REPAIR	-2.93	0.00E+00
REACTOME_SARS_COV_2_INFECTION	-2.93	0.00E+00
WP_5Q35_COPY_NUMBER_VARIATION	-2.91	0.00E+00
HALLMARK_DNA_REPAIR	-2.91	0.00E+00

REACTOME_CHROMATIN_MODIFYING_ENZYMES	-2.90	0.00E+00
REACTOME_HOMOLOGY_DIRECTED_REPAIR	-2.90	0.00E+00
REACTOME_SIGNALING_BY_INTERLEUKINS	-2.87	0.00E+00
REACTOME_RNA_POLYMERASE_II_PRE_TRANSCRIPTION_EVENTS	-2.85	0.00E+00
REACTOME_TRANSPORT_OF_MATURE_MRNAS_DERIVED_FROM_INTRONLESS_TRANSCRIPTS	-2.84	0.00E+00
KEGG_NUCLEOTIDE_EXCISION_REPAIR	-2.84	0.00E+00
KEGG_PYRIMIDINE_METABOLISM	-2.84	0.00E+00
REACTOME_TELOMERE_C_STRAND_LAGGING_STRAND_SYNTHESIS	-2.83	0.00E+00
KEGG_MISMATCH_REPAIR	-2.83	0.00E+00
WP_AMYOTROPHIC_LATERAL_SCLEROSIS_ALS	-2.82	0.00E+00
REACTOME_SARS_COV_1_INFECTION	-2.82	0.00E+00
KEGG_PROPANOATE_METABOLISM	-2.82	0.00E+00
REACTOME_TRANSCRIPTIONAL_REGULATION_BY_SMALL_RNAS	-2.81	0.00E+00
REACTOME_TRANSLESION_SYNTHESIS_BY_POLK	-2.81	0.00E+00
REACTOME_TRANSLESION_SYNTHESIS_BY_Y_FAMILY_DNA_POLYMERASES_BYPASSES_LESIONS_ON_DNA_TEMPLATE	-2.80	0.00E+00
REACTOME_DNA_STRAND_ELONGATION	-2.79	0.00E+00
REACTOME_RHOBTB2_GTPASE_CYCLE	-2.78	0.00E+00
KEGG_OOCYTE_MEIOSIS	-2.76	0.00E+00
REACTOME_FORMATION_OF_THE_EARLY_ELONGATION_COMPLEX	-2.75	0.00E+00
REACTOME_VIRAL_MESSENGER_RNA_SYNTHESIS	-2.75	0.00E+00
REACTOME_TRNA_PROCESSING_IN_THE_NUCLEUS	-2.75	0.00E+00
WP_NUCLEOTIDE_EXCISION_REPAIR	-2.74	0.00E+00
REACTOME_NUCLEAR_ENVELOPE_NE_REASSEMBLY	-2.74	0.00E+00
REACTOME_DISEASES_OF_SIGNAL_TRANSDUCTION_BY_GROWTH_FACTOR_RECEPTORS_AND_SECOND_MESSENGERS	-2.74	0.00E+00
REACTOME_LAGGING_STRAND_SYNTHESIS	-2.73	0.00E+00
REACTOME_PROTEIN_UBIQUITINATION	-2.73	0.00E+00
BIOCARTA_FAS_PATHWAY	-2.73	0.00E+00
REACTOME_ABORTIVE_ELONGATION_OF_HIV_1_TRANSCRIPT_IN_THE_ABSENCE_OF_TAT	-2.73	0.00E+00
KEGG_MEDICUS_REFERENCE_DNA_REPLICATION_TERMINATION	-2.72	0.00E+00
REACTOME_FORMATION_OF_RNA_POL_II_ELONGATION_COMPLEX	-2.72	0.00E+00
KEGG_MEDICUS_REFERENCE_ORIGIN_UNWINDING_AND_ELONGATION	-2.72	0.00E+00
REACTOME_MICRORNA_MIRNA_BIOGENESIS	-2.72	0.00E+00
WP_DNA_REPLICATION	-2.72	0.00E+00
REACTOME_HIV_TRANSCRIPTION_ELONGATION	-2.71	0.00E+00
REACTOME_METABOLISM_OF_CARBOHYDRATES	-2.70	0.00E+00

KEGG_MEDICUS_REFERENCE_ELECTRON_TRANSFER_IN_COMPLEX_IV	-2.69	0.00E+00
WP_ALZHEIMER_39_S_DISEASE	-2.69	0.00E+00
KEGG_CITRATE_CYCLE_TCA_CYCLE	-2.69	0.00E+00
BIOCARTA_PROTEASOME_PATHWAY	-2.69	0.00E+00
WP_DNA_REPAIR_PATHWAYS_FULL_NETWORK	-2.68	0.00E+00
REACTOME_HCMV_EARLY_EVENTS	-2.68	0.00E+00
WP_ALZHEIMER_39_S_DISEASE_AND_MIRNA_EFFECTS	-2.67	0.00E+00
KEGG_DNA_REPLICATION	-2.67	0.00E+00
WP_BASE_EXCISION_REPAIR	-2.67	0.00E+00
KEGG_MEDICUS_REFERENCE_LONG_PATCH_BER	-2.66	0.00E+00
REACTOME_INTRACELLULAR_SIGNALING_BY_SECOND_MESSENGERS	-2.66	0.00E+00
KEGG_VALINE_LEUCINE_AND_ISOLEUCINE_DEGRADATION	-2.66	0.00E+00
KEGG_MEDICUS_REFERENCE_CENPE_INTERACTION_WITH_NDC80_COMPLEX	-2.65	0.00E+00
REACTOME_CILIUM_ASSEMBLY	-2.65	0.00E+00
REACTOME_ANCHORING_OF_THE_BASAL_BODY_TO_THE_PLASMA_MEMBRANE	-2.65	0.00E+00
KEGG_MEDICUS_REFERENCE_ELECTRON_TRANSFER_IN_COMPLEX_III	-2.64	0.00E+00
REACTOME_FORMATION_OF_INCISION_COMPLEX_IN_GG_NER	-2.63	0.00E+00
REACTOME_PROTEIN_FOLDING	-2.63	0.00E+00
KEGG_LYSINE_DEGRADATION	-2.62	0.00E+00
REACTOME_PURINE_RIBONUCLEOSIDE_MONOPHOSPHATE_BIOSYNTHESIS	-2.62	0.00E+00
KEGG_MEDICUS_REFERENCE_CITRATE_CYCLE_SECOND_CARBON_OXIDATION_2	-2.61	0.00E+00
REACTOME_DISORDERS_OF_TRANSMEMBRANE_TRANSPORTERS	-2.60	0.00E+00
REACTOME_TRNA_AMINOACYLATION	-2.60	0.00E+00
KEGG_BETA_ALANINE_METABOLISM	-2.59	1.00E-03
REACTOME_RNA_POLYMERASE_I_PROMOTER_ESCAPE	-2.59	1.00E-03
REACTOME_DNA_DAMAGE_BYPASS	-2.58	1.00E-03
WP_CILIARY_LANDSCAPE	-2.58	1.00E-03
REACTOME_GENE_SILENCING_BY_RNA	-2.58	1.00E-03
REACTOME_CLATHRIN_MEDIATED_ENDOCYTOSIS	-2.57	1.00E-03
REACTOME_TERMINATION_OF_TRANSLESION_DNA_SYNTHESIS	-2.57	1.00E-03
KEGG_RNA_DEGRADATION	-2.55	1.00E-03
WP_TCA_CYCLE_AKA_KREBS_OR_CITRIC_ACID_CYCLE	-2.55	1.00E-03
REACTOME_CRISTAE_FORMATION	-2.55	1.00E-03
KEGG_RNA_POLYMERASE	-2.55	1.00E-03
REACTOME_RNA_POLYMERASE_I_TRANSCRIPTION_TERMINATION	-2.55	1.00E-03

REACTOME_MET_ACTIVATES_PTK2_SIGNALING	-2.54	1.00E-03
REACTOME_REGULATION_OF_TP53_ACTIVITY	-2.54	1.00E-03
REACTOME_INTERACTIONS_OF_REV_WITH_HOST_CELLULAR_PROTEINS	-2.53	1.00E-03
KEGG_PYRUVATE_METABOLISM	-2.52	1.00E-03
WP_GLYCOLYSIS_AND_GLUCONEOGENESIS	-2.52	1.00E-03
REACTOME_DEADENYLATION_DEPENDENT_MRNA_DECAY	-2.51	1.00E-03
REACTOME_POLYMERASE_SWITCHING_ON_THE_C_STRAND_OF_THE_TELOMERE	-2.51	1.00E-03
REACTOME_FORMATION_OF_TUBULIN_FOLDING_INTERMEDIATES_BY_CCT_TRIC	-2.50	1.00E-03
REACTOME_HIV_ELONGATION_ARREST_AND_RECOVERY	-2.50	1.00E-03
KEGG_MEDICUS_REFERENCE_DISASSEMBLY_OF_MCC	-2.50	1.00E-03
REACTOME_RECOGNITION_OF_DNA_DAMAGE_BY_PCNA_CONTAINING_REPLICATION_COMPLEX	-2.49	1.00E-03
REACTOME_MHC_CLASS_II_ANTIGEN_PRESENTATION	-2.49	1.00E-03
REACTOME_TELOMERE_EXTENSION_BY_TELOMERASE	-2.49	1.00E-03
REACTOME_DNA_DOUBLE_STRAND_BREAK_RESPONSE	-2.48	1.00E-03
REACTOME_TRANSCRIPTION_OF_THE_HIV_GENOME	-2.48	1.00E-03
REACTOME_SLBP_DEPENDENT_PROCESSING_OF_REPLICATION_DEPENDENT_HISTONE_PRE_MRNAS	-2.48	1.00E-03
REACTOME_CITRIC_ACID_CYCLE_TCA_CYCLE	-2.48	1.00E-03
KEGG_MEDICUS_VARIANT_MUTATION_CAUSED_ABERRANT_HTT_TO_ELECTRON_TRANSFER_IN_COMPLEX_III	-2.47	1.00E-03
REACTOME_B_WICH_COMPLEX_POSITIVELY_REGULATES_RRNA_EXPRESSION	-2.47	1.00E-03
BIOCARTA_CPSF_PATHWAY	-2.47	1.00E-03
WP_CELLULAR_PROTEOSTASIS	-2.47	1.00E-03
REACTOME_EPHB_MEDIATED_FORWARD_SIGNALING	-2.46	1.00E-03
KEGG_MEDICUS_REFERENCE_HOMOLOGOUS_RECOMBINATION	-2.46	1.00E-03
KEGG_MEDICUS_ENV_FACTOR_ARSENIC_TO_ELECTRON_TRANSFER_IN_COMPLEX_IV	-2.45	1.00E-03
WP_TNF_ALPHA_SIGNALING_PATHWAY	-2.45	1.00E-03
REACTOME_SIGNALING_BY_FGFR2_IIIA_TM	-2.45	1.00E-03
REACTOME_RMTS_METHYLATE_HISTONE_ARGININES	-2.44	1.00E-03
REACTOME_TRNA_MODIFICATION_IN_THE_MITOCHONDRION	-2.44	1.00E-03
REACTOME_MRNA_CAPPING	-2.44	1.00E-03
REACTOME_APC_C_CDC20_MEDIATED_DEGRADATION_OF_CYCLIN_B	-2.44	2.00E-03
REACTOME_ISG15_ANTIVIRAL_MECHANISM	-2.44	2.00E-03
REACTOME_HSP90_CHAPERONE_CYCLE_FOR_STEROID_HORMONE_RECEPTORS_SHR_IN_THE_PRESENCE_OF_LIGAND	-2.43	2.00E-03
KEGG_MEDICUS_VARIANT_MUTATION_CAUSED_ABERRANT_ABETA_TO_ELECTRON_TRANSFER_IN_COMPLEX_IV	-2.42	2.00E-03
KEGG_TRYPTOPHAN_METABOLISM	-2.42	2.00E-03

KEGG_MEDICUS_REFERENCE_NUCLEAR_EXPORT_OF_MRNA	-2.42	2.00E-03
REACTOME_MAPK_FAMILY_SIGNALING_CASCADES	-2.41	2.00E-03
REACTOME_CLASS_I_MHC_MEDIATED_ANTIGEN_PROCESSING_PRESENTATION	-2.41	2.00E-03
WP_REGULATION_OF_SISTER_CHROMATID_SEPARATION_AT_THE_METAPHASE_ANAPHASE_TRANSITION	-2.41	2.00E-03
KEGG_MEDICUS_REFERENCE_CORE_NER_REACTION	-2.41	2.00E-03
REACTOME_SUMOYLATION	-2.41	2.00E-03
KEGG_CELL_CYCLE	-2.41	2.00E-03
REACTOME_PROTEIN_LOCALIZATION	-2.41	2.00E-03
REACTOME_INHIBITION_OF_DNA_RECOMBINATION_AT_TELOMERE	-2.40	2.00E-03
REACTOME_APC_CDC20_MEDIATED_DEGRADATION_OF_NEK2A	-2.40	2.00E-03
PID_LKB1_PATHWAY	-2.40	2.00E-03
KEGG_PROGESTERONE_MEDIATED_OOCYTE_MATURATION	-2.40	2.00E-03
REACTOME_MITOCHONDRIAL_TRNA_AMINOACYLATION	-2.40	2.00E-03
KEGG_MEDICUS_REFERENCE_TRANSCRIPTION_COUPLED_NER	-2.40	2.00E-03
KEGG_BASE_EXCISION_REPAIR	-2.39	2.00E-03
REACTOME_SIGNALING_BY_FGFR	-2.39	2.00E-03
BIOCARTA_MALATEX_PATHWAY	-2.38	2.00E-03
REACTOME_TRANSPORT_OF_THE_SLBP_DEPENDANT_MATURE_MRNA	-2.38	2.00E-03
REACTOME_ASSEMBLY_OF_COLLAGEN_FIBRILS_AND_OTHER_MULTIMERIC_STRUCTURES	-2.37	2.00E-03
KEGG_MEDICUS_REFERENCE_COHESIN_DISSOCIATION_IN_ANAPHASE	-2.37	2.00E-03
REACTOME_ATTACHMENT_AND_ENTRY	-2.37	2.00E-03
KEGG_AMYOTROPHIC_LATERAL_SCLEROSIS_ALS	-2.36	3.00E-03
REACTOME_GLUCONEOGENESIS	-2.35	3.00E-03
KEGG_MEDICUS_REFERENCE_RAB7_REGULATED_MICROTUBULE_MINUS_END_DIRECTED_TRANSPORT	-2.35	3.00E-03
REACTOME_CARGO_RECOGNITION_FOR_CLATHRIN_MEDIATED_ENDOCYTOSIS	-2.35	3.00E-03
WP_IL_24_SIGNALING_PATHWAY	-2.35	3.00E-03
KEGG_MEDICUS_REFERENCE_TELOMERE_ELONGATION	-2.34	3.00E-03
REACTOME_HCMV_INFECTION	-2.34	3.00E-03
WP_FAS_LIGAND_PATHWAY_AND_STRESS_INDUCTION_OF_HEAT_SHOCK_PROTEINS	-2.33	3.00E-03
WP_TCA_CYCLE_AND_DEFICIENCY_OF_PYRUVATE_DEHYDROGENASE_COMPLEX_PDHC	-2.33	3.00E-03
REACTOME_COPI_INDEPENDENT_GOLGI_TO_ER_RETROGRADE_TRAFFIC	-2.33	3.00E-03
KEGG_MEDICUS_REFERENCE_MODIFYING_OF_CONDENSIN_II_SUBUNITS	-2.33	3.00E-03
WP_METABOLIC_EPILEPTIC_DISORDERS	-2.33	3.00E-03
REACTOME_FGFR2_MUTANT_RECEPTOR_ACTIVATION	-2.33	3.00E-03

REACTOME_PHOSPHORYLATION_OF_THE_APC_C	-2.32	3.00E-03
WP_AEROBIC_GLYCOLYSIS	-2.32	3.00E-03
PID_AURORA_A_PATHWAY	-2.31	3.00E-03
WP_CELL_CYCLE	-2.31	3.00E-03
REACTOME_METABOLISM_OF_NUCLEOTIDES	-2.31	4.00E-03
REACTOME_HIV_TRANSCRIPTION_INITIATION	-2.30	4.00E-03
BIOCARTA_SUMO_PATHWAY	-2.30	4.00E-03
KEGG_MEDICUS_PATHOGEN_HTLV_1_TAX_TO_SPINDLE_ASSEMBLY_CHECKPOINT_SIGNALING	-2.30	4.00E-03
WP_MITOCHONDRIAL_COMPLEX_IV_ASSEMBLY	-2.30	4.00E-03
REACTOME_SUMOYLATION_OF_RNA_BINDING_PROTEINS	-2.30	4.00E-03
KEGG_AMINOACYL_TRNA_BIOSYNTHESIS	-2.29	4.00E-03
KEGG_MEDICUS_REFERENCE_MICROTUBULE_DEPOLYMERIZATION	-2.29	4.00E-03
REACTOME_ESTROGEN_DEPENDENT_GENE_EXPRESSION	-2.29	4.00E-03
KEGG_MEDICUS_REFERENCE_DEPHOSPHORYLATION_OF_KINETOCHORE	-2.29	4.00E-03
REACTOME_RHO_GTPASE_CYCLE	-2.27	4.00E-03
WP_TRANSLATION_INHIBITORS_IN_CHRONICALLY_ACTIVATED_PDGFRA_CELLS	-2.27	4.00E-03
REACTOME_RECYCLING_PATHWAY_OF_L1	-2.27	4.00E-03
REACTOME_COLLAGEN_FORMATION	-2.27	4.00E-03
REACTOME_MRNA_DECAY_BY_3_TO_5_EXORIBONUCLEASE	-2.27	4.00E-03
PID_HEDGEHOG_GLI_PATHWAY	-2.27	4.00E-03
REACTOME_SUMO_IS_TRANSFERRED_FROM_E1_TO_E2_UBE2I_UBC9	-2.27	4.00E-03
REACTOME_PIWI_INTERACTING_RNA_PIRNA_BIOGENESIS	-2.26	5.00E-03
REACTOME_TRANSCRIPTIONAL_REGULATION_OF_TESTIS_DIFFERENTIATION	-2.26	5.00E-03
WP_CORI_CYCLE	-2.26	5.00E-03
REACTOME_MITOCHONDRIAL_BIOGENESIS	-2.26	5.00E-03
REACTOME_PROCESSIVE_SYNTHESIS_ON_THE_LAGGING_STRAND	-2.26	5.00E-03
REACTOME_NUCLEAR_IMPORT_OF_REV_PROTEIN	-2.26	5.00E-03
REACTOME_SIGNALING_BY_FGFR2	-2.25	5.00E-03
REACTOME_POSTMITOTIC_NUCLEAR_PORE_COMPLEX_NPC_REFORMATION	-2.25	5.00E-03
KEGG_MEDICUS_REFERENCE_ORGANIZATION_OF_THE_OUTER_KINETOCHORE	-2.25	5.00E-03
HALLMARK_PI3K_AKT_MTOR_SIGNALING	-2.24	5.00E-03
REACTOME_ACTIVATION_OF_ATR_IN_RESPONSE_TO_REPLICATION_STRESS	-2.24	5.00E-03
REACTOME_SUMOYLATION_OF_CHROMATIN_ORGANIZATION_PROTEINS	-2.24	5.00E-03
KEGG_MEDICUS_REFERENCE_SPINDLE_ASSEMBLY_CHECKPOINT_SIGNALING	-2.24	5.00E-03

REACTOME_SUMO_IS_CONJUGATED_TO_E1_UBA2_SAE1	-2.23	5.00E-03
REACTOME_SELECTIVE_AUTOPHAGY	-2.23	5.00E-03
REACTOME_COLLAGEN_BIOSYNTHESIS_AND_MODIFYING_ENZYMES	-2.23	5.00E-03
REACTOME_GENE_AND_PROTEIN_EXPRESSION_BY_JAK_STAT_SIGNALING_AFTER_INTERLEUKIN_12_STIMULATION	-2.23	6.00E-03
KEGG_VALINE_LEUCINE_AND_ISOLEUCINE_BIOSYNTHESIS	-2.22	6.00E-03
REACTOME_KSRP_KHSRP_BINDS_AND_DESTABILIZES_MRNA	-2.22	6.00E-03
KEGG_BUTANOATE_METABOLISM	-2.22	6.00E-03
REACTOME_CONDENSATION_OF_PROMETAPHASE_CHROMOSOMES	-2.22	6.00E-03
REACTOME_RNA_POLYMERASE_II_TRANSCRIBES_SNRNA_GENES	-2.21	6.00E-03
WP_GASTRIC_CANCER_NETWORK_1	-2.21	6.00E-03
BIOCARTA_IL12_PATHWAY	-2.21	6.00E-03
BIOCARTA_KREB_PATHWAY	-2.21	6.00E-03
KEGG_MEDICUS_REFERENCE_MICROTUBULE_NUCLEATION	-2.20	7.00E-03
REACTOME_FORMATION_OF_ATP_BY_CHEMIOSMOTIC_COUPLING	-2.20	7.00E-03
REACTOME_TFAP2A_ACTS_AS_A_TRANSCRIPTIONAL_REPRESSOR_DURING_RETINOIC_ACID_INDUCED_CELL_DIFFERENTIATION	-2.20	7.00E-03
PID_PLK1_PATHWAY	-2.19	7.00E-03
REACTOME_NS1_MEDIATED_EFFECTS_ON_HOST_PATHWAYS	-2.19	7.00E-03
WP_GABA_RECEPTOR_SIGNALING	-2.19	7.00E-03
REACTOME_NEGATIVE_EPIGENETIC_REGULATION_OF_RRNA_EXPRESSION	-2.18	8.00E-03
WP_REGULATION_OF_APOPTOSIS_BY_PARATHYROID_HORMONE_RELATED_PROTEIN	-2.18	8.00E-03
REACTOME_ACTIVATION_OF_THE_PRE_REPLICATIVE_COMPLEX	-2.18	8.00E-03
REACTOME_RHOBTB_GTPASE_CYCLE	-2.18	8.00E-03
REACTOME_JNK_C_JUN_KINASES_PHOSPHORYLATION_AND_ACTIVATION_MEDIATED_BY_ACTIVATED_HUMAN_TAK1	-2.18	8.00E-03
REACTOME_INTERFERON_SIGNALING	-2.18	8.00E-03
REACTOME_INTERACTIONS_OF_VPR_WITH_HOST_CELLULAR_PROTEINS	-2.18	8.00E-03
REACTOME_REGULATION_OF_TP53_EXPRESSION_AND_DEGRADATION	-2.18	8.00E-03
WP_ALPHA_6_BETA_4_SIGNALING_PATHWAY	-2.18	8.00E-03
REACTOME_AGGREPHAGY	-2.18	8.00E-03
PID_P53_REGULATION_PATHWAY	-2.17	8.00E-03
REACTOME_EXPORT_OF_VIRAL_RIBONUCLEOPROTEINS_FROM_NUCLEUS	-2.16	9.00E-03
WP_AMINO_ACID_METABOLISM	-2.16	9.00E-03
KEGG_COLORECTAL_CANCER	-2.16	9.00E-03
REACTOME_ACTIVATION_OF_ANTERIOR_HOX_GENES_IN_HINDBRAIN_DEVELOPMENT_DURING_EARLY_EMBRYOGENESIS	-2.16	9.00E-03
WP_THIAMINE_METABOLIC_PATHWAYS	-2.15	9.00E-03

REACTOME_MET_PROMOTES_CELL_MOTILITY	-2.15	9.00E-03
PID_HIF1A_PATHWAY	-2.14	1.00E-02
KEGG_MEDICUS_REFERENCE_BRANCHING_MICROTUBULE_NUCLEATION	-2.14	1.00E-02
REACTOME_CYCLIN_A_B1_B2_ASSOCIATED_EVENTS_DURING_G2_M_TRANSITION	-2.14	1.00E-02
HALLMARK_REACTIVE_OXYGEN_SPECIES_PATHWAY	-2.14	1.00E-02
PID_AR_NONGENOMIC_PATHWAY	-2.14	1.00E-02
BIOCARTA_RAN_PATHWAY	-2.13	1.00E-02
BIOCARTA_PYK2_PATHWAY	-2.13	1.00E-02
KEGG_CARDIAC_MUSCLE_CONTRACTION	-2.13	1.00E-02
KEGG_MEDICUS_VARIANT_MUTATION_INACTIVATED_DJ1_TO_FAS_JNK_SIGNALING_PATWHAY	-2.13	1.00E-02
PID_INTEGRIN4_PATHWAY	-2.13	1.00E-02
KEGG_MEDICUS_REFERENCE_TRAIP_DEPENDENT_REPLISOME_DISASSEMBLY	-2.12	1.10E-02
REACTOME_REGULATION_OF_GLUCOKINASE_BY_GLUCOKINASE_REGULATORY_PROTEIN	-2.12	1.10E-02
REACTOME_POLYMERASE_SWITCHING	-2.12	1.10E-02
WP_MITOCHONDRIAL_COMPLEX_II_ASSEMBLY	-2.12	1.10E-02
KEGG_MEDICUS_PATHOGEN_HIV_TAT_NEF_TO_CROSSTALK_BETWEEN_EXTRINSIC_AND_INTRINSIC_APOPTOTIC_PATHWAYS	-2.11	1.10E-02
REACTOME_INHIBITION_OF_THE_PROTEOLYTIC_ACTIVITY_OF_APC_C_REQUIRED_FOR_THE_ONSET_OF_ANAPHASE_BY_MITOTIC_SPINDLE_CHECKPOINT_COMPONENTS	-2.11	1.10E-02
REACTOME_DETOXIFICATION_OF_REACTIVE_OXYGEN_SPECIES	-2.11	1.20E-02
REACTOME_HATS_ACETYLATE_HISTONES	-2.11	1.20E-02
KEGG_MEDICUS_REFERENCE_CROSSTALK_BETWEEN_EXTRINSIC_AND_INTRINSIC_APOPTOTIC_PATHWAYS	-2.11	1.20E-02
REACTOME_COOPERATION_OF_PDCL_PHLP1_AND_TRIC_CCT_IN_G_PROTEIN_BETA_FOLDING	-2.11	1.20E-02
WP_EUKARYOTIC_TRANSCRIPTION_INITIATION	-2.11	1.20E-02
REACTOME_E3_UBIQUITIN_LIGASES_UBIQUITINATE_TARGET_PROTEINS	-2.10	1.20E-02
PID_PDGFRB_PATHWAY	-2.10	1.20E-02
REACTOME_PROCESSING_OF_INTRONLESS_PRE_MRNAS	-2.10	1.20E-02
REACTOME_PYRUVATE_METABOLISM_AND_CITRIC_ACID_TCA_CYCLE	-2.10	1.20E-02
WP_SOMATIC_SEX_DETERMINATION	-2.10	1.20E-02
REACTOME_MISMATCH_REPAIR	-2.10	1.30E-02
REACTOME_REGULATION_OF_TP53_ACTIVITY_THROUGH_ACETYLATION	-2.09	1.30E-02
REACTOME_SIGNALING_BY_NUCLEAR_RECEPTORS	-2.09	1.30E-02
REACTOME_SUMOYLATION_OF_SUMOYLATION_PROTEINS	-2.09	1.30E-02
REACTOME_EPH_EPHRIN_SIGNALING	-2.09	1.30E-02
BIOCARTA_AKAP95_PATHWAY	-2.09	1.30E-02

KEGG_LIMONENE_AND_PINENE_DEGRADATION	-2.08	1.30E-02
REACTOME_ESR_MEDIATED_SIGNALING	-2.08	1.40E-02
REACTOME_NUCLEAR_PORE_COMPLEX_NPC_DISASSEMBLY	-2.07	1.40E-02
PID_EPHA2_FWD_PATHWAY	-2.07	1.40E-02
PID_IGF1_PATHWAY	-2.07	1.40E-02
REACTOME_TOLL_LIKE_RECEPTOR_TLR1_TLR2_CASCADE	-2.06	1.50E-02
WP_AMP_ACTIVATED_PROTEIN_KINASE_SIGNALING	-2.06	1.50E-02
REACTOME_SUMOYLATION_OF_DNA_DAMAGE_RESPONSE_AND_REPAIR_PROTEINS	-2.06	1.50E-02
REACTOME_EPH_EPHRIN_MEDIATED_REPULSION_OF_CELLS	-2.06	1.60E-02
WP_LEUCINE_ISOLEUCINE_AND_VALINE_METABOLISM	-2.05	1.60E-02
REACTOME_INTERLEUKIN_12_SIGNALING	-2.05	1.70E-02
PID_RAC1_PATHWAY	-2.04	1.70E-02
REACTOME_L1CAM_INTERACTIONS	-2.04	1.70E-02
BIOCARTA_GLEEVEC_PATHWAY	-2.04	1.70E-02
REACTOME_SIGNALING_BY_FGFR2_IN_DISEASE	-2.04	1.70E-02
BIOCARTA_TNFR1_PATHWAY	-2.04	1.70E-02
REACTOME_DARPP_32_EVENTS	-2.04	1.70E-02
REACTOME_FORMATION_OF_WDR5_CONTAINING_HISTONE_MODIFYING_COMPLEXES	-2.03	1.80E-02
WP_EFFECT_OF_PROGERIN_ON_GENES_INVOLVED_IN_HUTCHINSON_GILFORD_PROGERIA_SYNDROME	-2.03	1.80E-02
KEGG_MEDICUS_VARIANT_MUTATION_CAUSED_ABERRANT_ABETA_TO_CROSSTALK_BETWEEN_EXTRINSIC_AND_INTRINSIC_APOPTOTIC_PATHWAYS	-2.03	1.90E-02
KEGG_MEDICUS_REFERENCE_COHESIN_DISSOCIATION_IN_PROPHASE	-2.02	1.90E-02
REACTOME_AZATHIOPRINE_ADME	-2.02	1.90E-02
WP_COHESIN_COMPLEX_CORNELIA_DE_LANGE_SYNDROME	-2.02	2.00E-02
BIOCARTA_D4GDI_PATHWAY	-2.01	2.00E-02
KEGG_MEDICUS_REFERENCE_OKAZAKI_FRAGMENT_MATURATION	-2.01	2.00E-02
REACTOME_ABERRANT_REGULATION_OF_MITOTIC_EXIT_IN_CANCER_DUE_TO_RB1_DEFECTS	-2.01	2.00E-02
REACTOME_COSTIMULATION_BY_THE_CD28_FAMILY	-2.01	2.00E-02
REACTOME_INTEGRATION_OF_PROVIRUS	-2.01	2.00E-02
KEGG_MEDICUS_REFERENCE_BASE_EXCISION_AND_STRAND_CLEAVAGE_BY_BIFUNCTIONAL_GLYCOSYLASE	-2.01	2.00E-02
REACTOME_HDACS_DEACETYLATE_HISTONES	-2.01	2.00E-02
PID_MET_PATHWAY	-2.01	2.00E-02
BIOCARTA_DNAFRAGMENT_PATHWAY	-2.01	2.10E-02
REACTOME_APOPTOSIS_INDUCED_DNA_FRAGMENTATION	-2.01	2.10E-02
REACTOME_HDR_THROUGH_HOMOLOGOUS_RECOMBINATION_HRR	-2.01	2.10E-02

REACTOME_CONDENSATION_OF_PROPHASE_CHROMOSOMES	-2.00	2.10E-02
KEGG_MEDICUS_ENV_FACTOR_METALS_TO_JNK_SIGNALING_PATHWAY	-2.00	2.10E-02
REACTOME_DEACTIVATION_OF_THE_BETA_CATENIN_TRANSACTIVATING_COMPLEX	-2.00	2.10E-02
WP_APOPTOSIS_MODULATION_AND_SIGNALING	-2.00	2.10E-02
REACTOME_PKR_MEDIATED_SIGNALING	-2.00	2.10E-02
WP_KLEEFSTRA_SYNDROME	-1.99	2.20E-02
REACTOME_MITOTIC_PROPHASE	-1.99	2.20E-02
HALLMARK_GLYCOLYSIS	-1.99	2.20E-02
REACTOME_TYPE_I_HEMIDESMOSOME_ASSEMBLY	-1.98	2.30E-02
REACTOME_CONVERSION_FROM_APC_C_CDC20_TO_APC_C_CDH1_IN_LATE_ANAPHASE	-1.98	2.30E-02
REACTOME_INTRINSIC_PATHWAY_FOR_APOPTOSIS	-1.98	2.30E-02
WP_NANOMATERIAL_INDUCED_APOPTOSIS	-1.98	2.40E-02
PID_SYNDECAN_1_PATHWAY	-1.98	2.40E-02
REACTOME_COLLAGEN_CHAIN_TRIMERIZATION	-1.97	2.40E-02
REACTOME_ERCC6_CSB_AND_EHMT2_G9A_POSITIVELY_REGULATE_RRNA_EXPRESSION	-1.97	2.50E-02
BIOCARTA_CASPASE_PATHWAY	-1.97	2.50E-02
WP_HYPOTHETICAL_CRANIOFACIAL_DEVELOPMENT_PATHWAY	-1.96	2.50E-02
BIOCARTA_ARF_PATHWAY	-1.96	2.60E-02
WP_GLYCOLYSIS_IN_SENESCENCE	-1.96	2.60E-02
REACTOME_REGULATION_OF_GLYCOLYSIS_BY_FRUCTOSE_2_6_BISPHOSPHATE_METABOLISM	-1.96	2.70E-02
PID_ATR_PATHWAY	-1.96	2.70E-02
WP_HISTONE_MODIFICATIONS	-1.95	2.70E-02
PID_EPHRINB_REV_PATHWAY	-1.95	2.70E-02
REACTOME_TP53_REGULATES_METABOLIC_GENES	-1.95	2.70E-02
REACTOME_NUCLEAR_ENVELOPE_BREAKDOWN	-1.95	2.70E-02
WP_MIR_509_3P_ALTERATION_OF_YAP1_ECM_AXIS	-1.95	2.70E-02
KEGG_MEDICUS_REFERENCE_DYNEIN_RECRUITMENT_TO_THE_KINETOCHORE	-1.94	2.80E-02
KEGG_AXON_GUIDANCE	-1.94	2.80E-02
PID_LIS1_PATHWAY	-1.94	2.80E-02
REACTOME_SYNTHESIS_OF_ACTIVE_UBIQUITIN_ROLES_OF_E1_AND_E2_ENZYMES	-1.94	2.80E-02
WP_NAD_METABOLISM_IN_ONCOGENE_INDUCED_SENESCENCE_AND_MITOCHONDRIAL_DYSFUNCTION_ASSOCIATED_SENESCENCE	-1.94	2.80E-02
REACTOME_DEFECTIVE_EXT2_CAUSES_EXOSTOSES_2	-1.94	2.80E-02
REACTOME_PROCESSING_OF_DNA_DOUBLE_STRAND_BREAK_ENDS	-1.94	2.80E-02
REACTOME_REGULATION_OF_TP53_ACTIVITY_THROUGH_PHOSPHORYLATION	-1.94	2.80E-02

REACTOME_RAF_ACTIVATION	-1.94	2.80E-02
BIOCARTA_VDR_PATHWAY	-1.94	2.90E-02
HALLMARK_FATTY_ACID_METABOLISM	-1.94	2.90E-02
WP_HIJACK_OF_UBIQUITINATION_BY_SARS_COV_2	-1.93	2.90E-02
KEGG_PENTOSE_PHOSPHATE_PATHWAY	-1.93	2.90E-02
KEGG_FOCAL_ADHESION	-1.93	2.90E-02
KEGG_APOPTOSIS	-1.93	3.00E-02
WP_PURINE_METABOLISM_AND_RELATED_DISORDERS	-1.93	3.00E-02
REACTOME_ASSEMBLY_OF_THE_ORC_COMPLEX_AT_THE_ORIGIN_OF_REPLICATION	-1.92	3.10E-02
WP_APOPTOSIS_MODULATION_BY_HSP70	-1.92	3.10E-02
PID_A6B1_A6B4_INTEGRIN_PATHWAY	-1.91	3.30E-02
PID_FAS_PATHWAY	-1.91	3.30E-02
REACTOME_POLB_DEPENDENT_LONG_PATCH_BASE_EXCISION_REPAIR	-1.91	3.30E-02
PID_RET_PATHWAY	-1.91	3.30E-02
PID_AVB3_INTEGRIN_PATHWAY	-1.90	3.40E-02
REACTOME_TP53_REGULATES_TRANSCRIPTION_OF_DNA_REPAIR_GENES	-1.89	3.60E-02
KEGG_MEDICUS_REFERENCE_KINETOCHORE_FIBER_ORGANIZATION	-1.89	3.60E-02
REACTOME_CELLULAR_SENESCENCE	-1.89	3.70E-02
KEGG_MEDICUS_PATHOGEN_SALMONELLA_SOPB_TO_ANXA2_S100A10_REGULATED_ACTIN_CYTOSKELETON	-1.89	3.70E-02
REACTOME_E2F_ENABLED_INHIBITION_OF_PRE_REPLICATION_COMPLEX_FORMATION	-1.88	3.80E-02
REACTOME_BETA_CATENIN_PHOSPHORYLATION_CASCADE	-1.88	3.80E-02
REACTOME_FACTORS_INVOLVED_IN_MEGAKARYOCYTE_DEVELOPMENT_AND_PLATELET_PRODUCTION	-1.88	3.90E-02
PID_INTEGRIN1_PATHWAY	-1.87	3.90E-02
KEGG_UBIQUITIN_MEDIATED_PROTEOLYSIS	-1.87	3.90E-02
REACTOME_E2F_MEDIATED_REGULATION_OF_DNA_REPLICATION	-1.87	4.00E-02
REACTOME_PROCESSING_AND_ACTIVATION_OF_SUMO	-1.87	4.00E-02
WP_CLEAR_CELL_RENAL_CELL_CARCINOMA_PATHWAYS	-1.87	4.10E-02
REACTOME_INTRAFLAGELLAR_TRANSPORT	-1.86	4.20E-02
REACTOME_CTLA4_INHIBITORY_SIGNALING	-1.86	4.20E-02
KEGG_MEDICUS_REFERENCE_ICOSLG_ICOS_PI3K_SIGNALING_PATHWAY	-1.86	4.20E-02
WP_MET_IN_TYPE_1_PAPILLARY_RENAL_CELL_CARCINOMA	-1.86	4.20E-02
BIOCARTA_IRES_PATHWAY	-1.86	4.20E-02
BIOCARTA_RNAPOL3_PATHWAY	-1.86	4.30E-02
REACTOME_GOLGI_TO_ER_RETROGRADE_TRANSPORT	-1.85	4.30E-02
WP_NITRIC_OXIDE_METABOLISM_IN_CYSTIC_FIBROSIS	-1.85	4.30E-02

REACTOME_OPIOID_SIGNALLING	-1.85	4.40E-02
BIOCARTA_SARS_PATHWAY	-1.85	4.40E-02
KEGG_MEDICUS_REFERENCE_CXCR4_GNB_G_PLCB_PKC_SIGNALING_PATHWAY	-1.85	4.40E-02
WP_NEPHROGENESIS	-1.85	4.50E-02
REACTOME_CREB1_PHOSPHORYLATION_THROUGH_THE_ACTIVATION_OF_CAMKII_CAMKK_CAMKIV_CASCASDE	-1.85	4.50E-02
REACTOME_KINESINS	-1.85	4.50E-02
WP_METABOLIC_REPROGRAMMING_IN_PANCREATIC_CANCER	-1.84	4.50E-02
WP_CELL_MIGRATION_AND_INVASION_THROUGH_P75NTR	-1.84	4.60E-02
BIOCARTA_AHSP_PATHWAY	-1.84	4.60E-02
BIOCARTA_NO1_PATHWAY	-1.84	4.60E-02
PID_ALPHA_SYNUCLEIN_PATHWAY	-1.84	4.60E-02
REACTOME_HCMV_LATE_EVENTS	-1.84	4.70E-02
REACTOME_INITIATION_OF_NUCLEAR_ENVELOPE_NE_REFORMATION	-1.83	4.90E-02
REACTOME_SIGNALING_BY_RECEPTOR_TYROSINE_KINASES	-1.83	4.90E-02
KEGG_MEDICUS_PATHOGEN_SHIGELLA_OSPG_TO_TNF_NFKB_SIGNALING_PATHWAY	-1.83	4.90E-02
KEGG_MEDICUS_REFERENCE_GLOBAL_GENOME_NER	-1.82	4.90E-02

Supplementary Table S2. List of the genes identified as different due to both *CERS2* KD and PMA treatment with their respective z-score and cluster.

Gene	Scramble - Vehicle	Scramble - PMA	CERS2 KD - Vehicle	CERS2 KD - PMA	Cluster
ABCB8	0.93	0.52	-1.36	-0.08	1
AC092807.3	0.03	1.11	-1.32	0.18	1
ACSL3	0.36	0.63	-1.49	0.49	1
AMFR	0.03	0.44	-1.39	0.93	1
ANKRD1	0.53	1.05	-1.23	-0.34	1
ARHGEF28	1.11	0.46	-1.19	-0.37	1
BEX1	0.94	0.76	-1.07	-0.63	1
CBLB	0.07	0.92	-1.41	0.43	1
CLASRP	1.18	-0.32	-1.18	0.32	1
COL12A1	0.89	0.77	-1.18	-0.47	1
COMT	0.43	0.96	-1.37	-0.02	1
CYTH3	1.17	0.43	-1.10	-0.50	1
DHCR7	0.25	0.04	-1.35	1.06	1
DVL2	1.23	0.20	-1.18	-0.24	1
EIF3F	0.81	0.63	-1.40	-0.03	1
EIF3FP3	0.81	0.26	-1.46	0.39	1
ENAH	0.68	0.84	-1.34	-0.19	1
EPG5	0.13	0.31	-1.40	0.96	1
ERG28	-0.08	0.43	-1.35	1.00	1
FADS1	0.49	0.94	-1.37	-0.06	1
FADS2	0.98	0.36	-1.38	0.04	1
FDPS	0.65	0.26	-1.48	0.57	1
FLNA	0.85	-0.10	-1.37	0.62	1
FOXO3	0.79	0.77	-1.32	-0.24	1
FPGS	0.87	-0.39	-1.23	0.75	1
FTH1	0.68	0.84	-1.34	-0.17	1
GJA1	-0.20	0.91	-1.32	0.61	1
GTF2IRD1	1.32	-0.13	-1.10	-0.09	1
H1FX	1.08	0.39	-1.29	-0.17	1
HERC2P2	1.13	-0.51	-1.11	0.48	1
HINFP	1.00	-0.24	-1.29	0.54	1
HSD17B7	0.17	0.48	-1.45	0.80	1
IDI1	0.99	0.71	-1.05	-0.64	1
ITGA3	0.30	0.42	-1.47	0.75	1
LACTB	0.55	0.39	-1.50	0.56	1

MICAL2	0.03	0.99	-1.37	0.36	1
MSMO1	0.52	0.24	-1.47	0.71	1
MTDH	-0.21	0.85	-1.32	0.69	1
MYL9	0.45	0.54	-1.50	0.51	1
NOP53	1.06	0.27	-1.34	0.02	1
NOTCH2	0.31	1.09	-1.30	-0.11	1
NR3C2	0.73	0.87	-1.26	-0.35	1
NUPR1	-0.40	0.93	-1.22	0.70	1
PARD3B	1.00	0.44	-1.34	-0.10	1
PEA15	0.33	1.06	-1.33	-0.07	1
PKN1	0.07	0.36	-1.39	0.96	1
PREB	0.99	0.54	-1.29	-0.24	1
PTPRF	0.88	0.81	-1.11	-0.58	1
RAB32	1.22	-0.03	-1.23	0.04	1
RBM3	1.12	0.12	-1.32	0.08	1
RND3	0.77	-0.32	-1.28	0.82	1
RNF185	0.32	0.45	-1.48	0.71	1
SCD	1.14	0.49	-1.06	-0.57	1
SEC23B	0.67	0.86	-1.33	-0.21	1
SHKBP1	1.20	0.18	-1.23	-0.15	1
SHMT2	0.46	1.06	-1.27	-0.25	1
SLC25A6	1.16	0.44	-1.10	-0.50	1
SMIM29	1.15	-0.30	-1.21	0.36	1
SQLE	0.09	0.26	-1.37	1.02	1
SREBF2	0.68	0.10	-1.45	0.66	1
TBC1D16	0.93	0.29	-1.42	0.20	1
TMEM161A	0.68	-0.32	-1.27	0.91	1
WBP2	0.89	-0.03	-1.39	0.53	1
ZNF579	0.69	-0.25	-1.31	0.87	1
ZNF703	1.07	0.43	-1.27	-0.23	1
ABLIM1	0.54	1.12	-0.63	-1.03	2
ADAR	-0.12	1.42	-0.91	-0.40	2
ADD3	0.12	1.32	-0.40	-1.04	2
ATAD2	-0.09	1.41	-0.39	-0.92	2
BST2	-0.70	1.31	-0.86	0.25	2
DCLRE1C	-0.89	1.42	-0.08	-0.45	2
DDX60L	-0.11	1.44	-0.75	-0.59	2
DIXDC1	-0.77	1.45	-0.15	-0.53	2
DTWD1	-0.55	1.50	-0.50	-0.45	2

DTX3L	-0.59	1.45	-0.73	-0.12	2
EIF2AK2	-0.44	1.45	-0.80	-0.21	2
ENDOD1	-1.10	1.28	-0.34	0.16	2
EZH2	-1.28	0.97	0.59	-0.28	2
FAM111B	-0.63	1.16	0.48	-1.01	2
FBXO5	-0.65	1.14	0.52	-1.00	2
FGF7	-0.75	1.47	-0.42	-0.30	2
GGH	-1.30	1.14	0.14	0.02	2
GLIPR1	-1.19	1.21	-0.27	0.25	2
HELZ2	-1.00	1.23	-0.60	0.37	2
HERC6	-0.15	1.46	-0.63	-0.68	2
IFI44L	-0.53	1.46	-0.74	-0.19	2
IFI6	-0.73	1.35	-0.79	0.17	2
IFIT1	0.38	1.23	-0.61	-0.99	2
IFIT5	-0.42	1.48	-0.73	-0.33	2
IFITM1	-0.80	1.38	-0.68	0.10	2
IFITM3	0.08	1.33	-0.37	-1.04	2
IL11	-0.60	1.47	-0.65	-0.22	2
IL6	-0.77	1.25	-0.84	0.36	2
IRF7	-0.93	1.11	-0.76	0.57	2
ISG15	0.02	1.40	-0.65	-0.78	2
JUP	-0.46	1.50	-0.44	-0.60	2
LY6E	-1.08	1.17	-0.52	0.43	2
MANEA	0.02	1.32	-0.25	-1.09	2
MSC	-0.84	1.12	-0.85	0.57	2
MT-ND3	-1.04	1.36	-0.14	-0.18	2
MT-ND5	-1.04	1.14	0.50	-0.61	2
MT-ND6	-1.09	1.18	0.41	-0.51	2
MT-RNR2	-1.18	0.82	0.83	-0.47	2
MT-TC	-1.40	0.91	0.45	0.04	2
MTAP	-0.06	1.32	-0.15	-1.11	2
MX1	-0.74	1.39	-0.72	0.06	2
MX2	-0.59	1.46	-0.72	-0.15	2
NETO2	-0.39	1.20	0.33	-1.14	2
NR4A1	-0.90	1.17	-0.76	0.50	2
NREP	-0.97	1.38	-0.40	-0.02	2
NUDT21	-0.25	1.01	0.52	-1.28	2
OAS1	-0.50	1.47	-0.72	-0.24	2
OAS2	-0.69	1.44	-0.67	-0.08	2

OAS3	-0.49	1.44	-0.82	-0.13	2
PARP12	-0.47	1.42	-0.86	-0.09	2
PARP14	-0.67	1.47	-0.57	-0.23	2
PARP9	-0.45	1.47	-0.74	-0.29	2
PDGFRA	-0.80	1.46	-0.38	-0.28	2
PKIA	-0.06	1.01	0.40	-1.35	2
PLSCR1	-0.45	1.44	-0.83	-0.16	2
POSTN	0.11	1.36	-0.87	-0.61	2
PSME2	-0.78	1.43	-0.57	-0.08	2
RBMXL1	0.03	1.32	-0.26	-1.09	2
RMI1	0.44	1.02	-0.15	-1.32	2
SAMD9	-0.15	1.44	-0.42	-0.86	2
SAMD9L	-0.35	1.49	-0.68	-0.46	2
SAMHD1	-0.69	1.47	-0.58	-0.20	2
SLFN5	-0.79	1.35	-0.72	0.16	2
SNX18	0.26	1.29	-0.60	-0.95	2
SP100	-0.75	1.34	-0.78	0.19	2
SP110	-1.03	1.26	-0.52	0.28	2
SPRY1	-1.00	1.35	0.07	-0.42	2
STAT1	-0.76	1.38	-0.70	0.09	2
TENT5A	-0.74	1.27	0.32	-0.85	2
TIPARP	-1.23	1.11	-0.30	0.42	2
TM4SF1	-0.69	1.49	-0.37	-0.43	2
TNFRSF11B	-0.18	1.46	-0.55	-0.73	2
TNFSF4	0.12	1.37	-0.77	-0.72	2
TRIM14	-0.17	1.43	-0.87	-0.39	2
TRIM22	-0.68	1.41	-0.74	0.00	2
TRPC4	-0.40	1.28	-1.08	0.19	2
UGCG	-0.16	1.46	-0.66	-0.63	2
USP18	-0.74	1.45	-0.58	-0.13	2
XAF1	-0.76	1.32	-0.79	0.23	2
ZNFX1	-0.83	0.86	0.87	-0.91	2
AGRN	-0.88	-0.69	1.29	0.29	3
AK4	-0.97	-0.70	1.13	0.54	3
ANTXR2	-1.36	-0.13	0.60	0.89	3
ATP2B4	-1.12	-0.56	0.91	0.77	3
BAG5	-1.39	0.07	0.98	0.34	3
BNIP3	-1.32	-0.06	1.08	0.30	3
C1QTNF6	-0.78	-0.68	1.39	0.07	3

C21orf58	-1.46	0.23	0.43	0.80	3
C3orf58	-1.18	-0.40	1.10	0.48	3
C4orf3	-1.30	-0.10	1.09	0.31	3
CCNE2	-1.44	0.79	0.54	0.11	3
CD99	-0.93	-0.70	1.21	0.42	3
CDH11	-1.07	-0.42	1.28	0.21	3
CHPF	-1.24	-0.30	0.47	1.07	3
CKB	-1.41	0.17	0.28	0.95	3
CSRNP1	-1.38	-0.01	0.43	0.96	3
CTHRC1	-1.11	-0.45	0.35	1.20	3
DEPP1	-1.38	0.10	0.28	1.00	3
DNAJC3	-1.40	-0.04	0.68	0.76	3
DPY19L2P2	-1.14	-0.20	1.29	0.05	3
EGR1	-1.27	-0.31	0.91	0.67	3
EMILIN1	-1.19	-0.36	0.43	1.13	3
ERRFI1	-0.84	-0.73	1.29	0.28	3
EXOC3L2	-1.27	-0.11	0.24	1.14	3
FAM107B	-1.29	-0.27	0.94	0.62	3
FLT1	-1.22	-0.41	0.88	0.76	3
FOSL1	-0.91	-0.65	1.29	0.27	3
G3BP2	-0.99	-0.54	1.30	0.23	3
GPR180	-1.22	-0.30	1.14	0.37	3
HIC1	-1.19	-0.42	0.54	1.06	3
HMMR	-1.24	-0.29	1.08	0.46	3
IKBIP	-1.49	0.62	0.36	0.52	3
IPMK	-0.94	-0.65	1.27	0.31	3
ITGA11	-0.97	-0.75	1.00	0.72	3
JPT2	-1.13	-0.16	1.30	-0.01	3
KDELC2	-0.72	-0.71	1.41	0.02	3
LOXL2	-0.90	-0.76	0.47	1.18	3
MICALL1	-0.44	-1.20	0.94	0.70	3
MINDY2	-1.09	-0.57	1.05	0.61	3
MSX2	-1.25	-0.07	1.19	0.13	3
NDRG1	-0.88	-0.79	1.14	0.53	3
NID2	-1.44	0.09	0.75	0.61	3
NLRP3	-1.10	0.01	1.32	-0.23	3
NRGN	-1.34	-0.14	0.54	0.94	3
NUMB	-0.91	-0.61	1.32	0.20	3
P4HA1	-1.35	-0.12	0.56	0.92	3

PAM	-1.46	0.31	0.36	0.79	3
PAPPA	-1.28	-0.31	0.76	0.83	3
PCDHGC3	-1.33	-0.02	0.29	1.07	3
PDK1	-1.01	-0.57	1.24	0.33	3
PECAM1	-0.81	-0.54	1.43	-0.08	3
PFKFB4	-1.42	0.01	0.74	0.67	3
PGF	-1.28	-0.30	0.70	0.89	3
PGK1	-1.39	0.34	0.97	0.08	3
PLAU	-0.79	-0.83	1.23	0.40	3
PLOD1	-1.11	-0.58	0.83	0.86	3
PNRC2	-0.95	-0.74	1.11	0.58	3
PPFIA4	-1.13	-0.54	0.68	0.99	3
PTCH1	-1.50	0.57	0.40	0.53	3
RECK	-1.40	-0.02	0.57	0.85	3
RIN2	-1.39	-0.02	0.54	0.87	3
RP2	-0.88	-0.62	1.35	0.14	3
RRM2	-1.01	-0.14	1.38	-0.23	3
SCARA3	-0.88	-0.75	1.22	0.41	3
SCARF1	-0.74	-0.71	1.39	0.07	3
SEMA4B	-0.56	-0.90	1.36	0.11	3
SEMA7A	-0.90	-0.79	1.09	0.61	3
SERPINF1	-1.26	-0.16	1.14	0.28	3
SH3TC1	-1.29	-0.09	1.13	0.25	3
SIPA1L1	-1.36	-0.15	0.76	0.74	3
SMAD5	-1.05	-0.64	0.99	0.70	3
SMIM3	-1.20	-0.42	0.99	0.63	3
SPOCD1	-0.63	-1.05	0.62	1.06	3
SPRED1	-1.24	-0.22	1.14	0.32	3
SPRY4	-1.37	0.12	0.21	1.04	3
SRSF10	-1.44	0.68	0.68	0.08	3
STC1	-1.25	0.02	1.20	0.02	3
TFPI2	-1.11	-0.59	0.80	0.89	3
TGFB1	-1.28	-0.26	0.55	0.99	3
THY1	-0.69	-0.81	1.36	0.15	3
TMEM167A	-1.33	-0.20	0.81	0.72	3
TMX3	-1.24	-0.32	1.04	0.52	3
TNS2	-1.24	-0.29	1.08	0.45	3
TPBG	-1.11	-0.57	0.97	0.71	3
VLDLR	-1.11	-0.44	0.34	1.21	3

ZNF367	-1.49	0.63	0.39	0.47	3
ABCA10	0.66	-0.50	1.00	-1.15	4
ABCA9	0.64	-0.59	1.03	-1.08	4
ABHD17C	-0.16	-0.81	1.45	-0.47	4
AC240274.1	0.82	-0.32	0.77	-1.28	4
ACER3	-0.53	-0.36	1.49	-0.60	4
ADGRL2	-0.14	-0.86	1.43	-0.43	4
AKAP11	0.32	0.60	0.56	-1.49	4
B4GALT5	0.46	-0.49	1.14	-1.11	4
CALM1	-0.51	0.18	1.32	-0.99	4
CAV1	0.58	-0.83	1.11	-0.86	4
CCL5	0.59	-0.37	1.01	-1.23	4
CD248	-0.61	-0.75	1.44	-0.08	4
CD44	-0.30	-0.75	1.47	-0.42	4
CD59	-0.28	-0.57	1.48	-0.63	4
CHST2	0.20	-1.30	1.13	-0.03	4
CLEC2B	-0.74	-0.55	1.46	-0.17	4
CNOT6	0.28	0.22	0.92	-1.42	4
COL4A5	0.82	-0.29	0.76	-1.29	4
CXCL5	0.54	-0.64	1.12	-1.02	4
DGAT2	-0.65	-0.57	1.48	-0.25	4
DUSP6	-0.26	-0.58	1.48	-0.64	4
ECE1	-0.07	-0.85	1.42	-0.50	4
EGLN3	-0.66	-0.58	1.47	-0.23	4
EIF5B	0.13	-0.44	1.33	-1.01	4
FER1L6	-0.89	-0.08	1.42	-0.44	4
FGFR1OP	-0.58	-0.02	1.41	-0.82	4
FSTL1	-0.38	-0.93	1.41	-0.10	4
GABBR2	-0.48	-0.77	1.46	-0.21	4
GALNT1	0.28	-0.29	1.19	-1.19	4
GGCX	0.40	0.02	0.96	-1.38	4
GNG11	-0.39	-0.21	1.44	-0.85	4
GOLM1	-0.62	-0.48	1.49	-0.40	4
GPX3	0.22	-0.22	1.21	-1.20	4
GRB10	0.95	-1.27	0.62	-0.30	4
GSN	-0.47	-0.67	1.49	-0.35	4
GXYLT1	-0.55	-0.73	1.46	-0.18	4
HBEGF	-0.46	-0.77	1.46	-0.23	4
HGF	0.39	-0.79	1.24	-0.84	4

ID3	-0.65	-0.69	1.44	-0.10	4
IFI16	-0.70	0.34	1.26	-0.90	4
IGFBP2	-0.37	-0.75	1.47	-0.36	4
IGFBP5	-0.55	-0.78	1.44	-0.10	4
IL1R1	0.29	-0.89	1.29	-0.68	4
IL33	0.84	-0.64	0.87	-1.06	4
ITGB8	0.17	-0.65	1.34	-0.86	4
LAMP3	0.34	-0.53	1.23	-1.04	4
LDHA	-0.77	-0.08	1.44	-0.58	4
LTBP1	-0.47	-0.92	1.39	0.00	4
LUCAT1	-0.28	0.39	1.12	-1.23	4
MAML3	0.02	-0.65	1.41	-0.77	4
MGAT5	-0.13	-0.59	1.45	-0.73	4
MSH6	-0.36	0.27	1.23	-1.14	4
MT-CO3	0.71	-0.44	0.93	-1.20	4
MTUS1	0.84	-0.64	0.87	-1.06	4
MYH15	-0.11	-0.29	1.39	-0.99	4
MYO1B	-0.07	-0.79	1.43	-0.57	4
NT5E	-0.82	-0.42	1.45	-0.21	4
OSBP2	-0.07	0.22	1.14	-1.28	4
PIK3R1	-0.37	-0.76	1.47	-0.35	4
PITPNB	-0.92	-0.22	1.42	-0.28	4
PPP1R3C	-0.22	0.06	1.29	-1.13	4
PTGFRN	0.05	-0.87	1.38	-0.56	4
PTX3	0.87	-0.98	0.86	-0.74	4
RAP1A	-0.18	0.32	1.13	-1.27	4
RBFOX2	-0.39	-0.35	1.48	-0.73	4
RIC8B	-0.29	0.52	1.03	-1.26	4
RNF145	0.37	0.25	0.83	-1.45	4
S100A4	0.08	-0.75	1.39	-0.71	4
SERPINB4	0.09	-0.33	1.32	-1.07	4
SERPINB8	-0.26	0.26	1.20	-1.20	4
SKP2	0.12	-0.48	1.34	-0.98	4
SMOC1	0.31	-0.48	1.24	-1.07	4
SNRK	-0.78	-0.02	1.42	-0.61	4
ST3GAL4	-0.88	-0.30	1.44	-0.25	4
TFDP1	0.38	0.26	0.81	-1.46	4
TMEM131L	-0.47	0.34	1.22	-1.09	4
TNC	0.03	-0.89	1.39	-0.52	4

TNFRSF19	-0.48	-0.59	1.50	-0.43	4
TNXB	-0.36	-0.89	1.43	-0.19	4
UGP2	0.36	0.62	0.51	-1.49	4
VEPH1	-0.01	-0.44	1.39	-0.94	4
ZNF185	0.10	-0.72	1.38	-0.75	4
ZNF436	-0.83	-0.43	1.45	-0.19	4
ACADVL	-0.87	-0.34	-0.23	1.44	5
ADM	-0.73	-0.34	-0.41	1.48	5
AP000695.1	-1.19	0.46	-0.38	1.11	5
AP5Z1	-0.48	-0.14	-0.83	1.44	5
ARFGAP1	-0.66	-0.70	-0.08	1.44	5
ATP6AP1	-0.85	-0.42	-0.18	1.44	5
BRI3	-0.60	-0.23	-0.65	1.47	5
C1orf43	-0.48	0.22	-1.02	1.29	5
CARS2	-0.16	-0.47	-0.81	1.45	5
CCND1	-1.21	0.51	-0.36	1.07	5
CD164	-1.39	0.41	0.03	0.95	5
CD81	-0.87	-0.02	-0.51	1.41	5
CEBPB	-0.78	0.05	-0.66	1.40	5
CEMIP	-1.26	0.23	-0.13	1.16	5
CLCN7	-0.80	-0.33	-0.34	1.46	5
COPG1	-0.25	0.20	-1.18	1.23	5
CSGALNACT2	-0.84	-0.67	0.16	1.35	5
CTNS	-0.72	-0.26	-0.50	1.47	5
CTSD	-0.89	-0.37	-0.17	1.43	5
CYBA	-1.36	0.24	0.08	1.04	5
CYP51A1	-0.33	0.47	-1.22	1.08	5
DAPK3	-0.82	-0.48	-0.15	1.44	5
DDIT4	-1.27	0.59	-0.29	0.98	5
DKK3	-0.92	-0.41	-0.08	1.41	5
DPP4	-1.18	0.81	-0.48	0.85	5
DPP7	-0.78	-0.35	-0.34	1.47	5
DUSP3	-0.86	-0.51	-0.04	1.41	5
EBP	-0.54	0.49	-1.08	1.14	5
F2R	-0.66	-0.01	-0.75	1.42	5
F2RL1	-0.84	0.08	-0.62	1.38	5
FBXL6	-0.69	-0.11	-0.65	1.45	5
FBXO32	-0.69	0.64	-1.00	1.06	5
GDF15	-0.75	-0.10	-0.59	1.44	5

GIGYF1	-0.08	-0.26	-1.03	1.36	5
GMPPA	-0.35	0.36	-1.18	1.17	5
HM13	-0.87	0.28	-0.70	1.29	5
HMGCR	-0.15	-0.13	-1.07	1.35	5
HMOX1	-0.43	-0.38	-0.67	1.49	5
HS1BP3	-0.65	-0.73	-0.05	1.43	5
IL24	-1.08	0.99	-0.61	0.70	5
INHBA	-1.16	0.91	-0.50	0.75	5
ITGAV	-1.23	0.61	-0.37	0.99	5
ITGB5	-1.15	0.39	-0.41	1.17	5
JUND	-0.02	0.17	-1.29	1.14	5
KLHL24	-0.36	0.67	-1.24	0.93	5
LAMP2	-0.43	0.49	-1.16	1.10	5
LCP1	-0.31	0.64	-1.27	0.94	5
LEMD2	-0.25	0.35	-1.24	1.14	5
LGALS3BP	-1.20	0.85	-0.45	0.79	5
MAFG	-0.82	-0.15	-0.48	1.44	5
MCOLN1	-0.78	-0.25	-0.43	1.46	5
MMP1	-1.18	0.62	-0.45	1.01	5
MMP2	-0.84	0.43	-0.80	1.21	5
MT1E	-1.20	0.75	-0.45	0.90	5
NAV2	-0.57	-0.76	-0.11	1.44	5
NEU1	-0.51	-0.42	-0.56	1.50	5
NFATC2	-1.28	0.20	-0.07	1.15	5
NPC1	-0.84	-0.45	-0.15	1.44	5
NPPB	-0.69	0.94	-1.02	0.77	5
PEPD	-0.39	-0.73	-0.36	1.48	5
PIP4P1	-0.69	-0.20	-0.58	1.47	5
PLA2G16	-0.46	0.01	-0.93	1.39	5
PLIN2	-0.73	-0.56	-0.18	1.46	5
PNPLA6	-0.70	-0.82	0.17	1.35	5
POR	-0.52	-0.60	-0.38	1.49	5
PPFIA1	0.16	-0.18	-1.20	1.23	5
PPP1R12C	-0.23	-0.61	-0.64	1.47	5
PSG4	-0.86	-0.16	-0.42	1.44	5
PTGS2	-0.90	-0.21	-0.33	1.43	5
PTPRK	-0.12	0.40	-1.32	1.04	5
PVR	-0.72	0.47	-0.93	1.18	5
RETREG2	-0.54	-0.24	-0.70	1.47	5

RHOQ	-0.82	-0.22	-0.41	1.45	5
SEC61A1	-0.86	0.30	-0.73	1.28	5
SEL1L	-0.75	0.31	-0.84	1.28	5
SERINC2	-0.43	0.66	-1.20	0.97	5
SIPA1L3	-0.14	-0.22	-1.02	1.38	5
SIRPA	-1.23	0.16	-0.13	1.20	5
SLC22A15	-0.04	0.00	-1.21	1.24	5
SLC2A1	-1.26	0.77	-0.35	0.84	5
SLC38A7	-0.30	-0.03	-1.03	1.36	5
SLC39A13	-0.66	-0.77	0.03	1.40	5
SLC3A2	-0.76	0.28	-0.82	1.30	5
SND1	-0.44	-0.14	-0.86	1.43	5
SNX8	-0.71	-0.79	0.14	1.36	5
SQSTM1	-0.34	0.61	-1.25	0.98	5
SRPRA	-0.80	-0.43	-0.23	1.46	5
TAGLN	-0.60	-0.10	-0.74	1.44	5
TBC1D7	-0.60	-0.93	0.21	1.32	5
TBX3	-0.02	-0.47	-0.90	1.40	5
TIMP1	-1.19	-0.05	-0.01	1.26	5
TMEM187	-0.08	-0.28	-1.02	1.37	5
TMEM97	-0.16	0.29	-1.26	1.13	5
TNFSF15	-1.39	0.51	-0.02	0.90	5
TOM1	-0.78	-0.22	-0.47	1.46	5
TRADD	0.15	-0.36	-1.08	1.29	5
UAP1L1	-0.63	-0.84	0.09	1.37	5
UBALD2	-0.80	-0.14	-0.50	1.45	5
UCN2	-1.42	0.64	0.02	0.76	5
VAC14	-0.50	-0.42	-0.58	1.50	5
VAT1	-0.43	-0.86	-0.14	1.43	5
VEGFA	-1.17	-0.21	0.13	1.25	5
VMP1	-0.42	0.53	-1.18	1.07	5
WBP1L	-0.47	-0.07	-0.88	1.42	5
YIF1B	-0.52	0.11	-0.94	1.35	5
ABCC1	1.23	0.39	-0.95	-0.66	6
ABLIM3	1.47	-0.49	-0.24	-0.74	6
ACLY	1.48	-0.65	-0.57	-0.26	6
ACO1	1.48	-0.34	-0.39	-0.75	6
ACSS2	1.39	-0.91	-0.49	0.02	6
ACTG1	1.38	0.10	-0.71	-0.77	6

ADAMTS1	0.66	0.50	0.33	-1.49	6
AHNAK2	1.12	-1.20	0.43	-0.35	6
AIFM2	1.40	-0.04	-0.92	-0.45	6
ANK3	1.02	-0.38	0.58	-1.22	6
ANXA10	1.34	-0.01	-0.25	-1.07	6
AXL	1.49	-0.36	-0.52	-0.61	6
BDKRB2	1.03	-1.12	0.63	-0.54	6
CD200	1.30	0.01	-0.16	-1.14	6
CLDN11	1.39	0.06	-0.63	-0.82	6
CMPK2	0.72	0.78	-0.12	-1.37	6
COL4A6	1.18	0.14	-0.05	-1.26	6
CRTC3	1.41	-0.96	-0.26	-0.19	6
CTU2	1.46	-0.52	-0.73	-0.21	6
DCBLD2	1.17	0.49	-0.86	-0.80	6
DCTN1	1.35	-0.87	0.14	-0.62	6
DDX54	1.45	-0.30	-0.84	-0.31	6
DDX58	1.09	0.46	-0.35	-1.21	6
DDX60	1.00	0.60	-0.38	-1.22	6
ECSIT	1.45	-0.85	-0.29	-0.30	6
EEF2	1.40	-0.02	-0.90	-0.47	6
ENC1	1.44	-0.09	-0.59	-0.76	6
ERCC2	1.43	-0.20	-0.91	-0.32	6
FASN	1.44	-0.11	-0.58	-0.75	6
FDFT1	1.39	-0.27	-0.99	-0.12	6
FKBP5	0.80	0.66	-0.07	-1.39	6
FLNB	1.24	-1.12	0.27	-0.39	6
FLOT1	1.43	-0.06	-0.69	-0.69	6
FST	0.95	0.58	-0.21	-1.31	6
GAB2	1.47	-0.23	-0.59	-0.65	6
GPRC5B	1.09	0.61	-0.92	-0.78	6
GTPBP6	1.47	-0.63	-0.23	-0.61	6
HEATR5B	1.48	-0.41	-0.74	-0.32	6
HERC5	0.58	1.00	-0.34	-1.24	6
HMGCS1	1.37	0.00	-0.99	-0.37	6
IFI44	0.93	0.62	-0.25	-1.30	6
IFIT3	1.22	-0.09	0.08	-1.22	6
INSIG1	1.38	-0.38	-0.97	-0.03	6
KIF22	1.45	-0.16	-0.81	-0.48	6
KLHL29	1.27	-1.11	0.18	-0.34	6

KLHL4	1.03	-0.13	0.43	-1.32	6
LPIN1	1.39	-0.41	-0.04	-0.94	6
LRRC75A-AS1	1.13	0.56	-0.89	-0.80	6
MT-CO1	1.01	0.27	0.09	-1.38	6
MVK	1.42	-0.93	-0.29	-0.20	6
MYCBP	0.59	0.52	0.38	-1.49	6
MYOF	1.42	-0.02	-0.62	-0.78	6
NCAPG	0.65	0.83	-0.12	-1.37	6
NEDD4L	1.26	0.29	-1.04	-0.50	6
OGDH	1.29	-1.00	0.23	-0.53	6
PEG10	0.90	-0.31	0.68	-1.28	6
PGD	1.27	-1.07	0.24	-0.44	6
PHB2	1.42	-0.02	-0.64	-0.76	6
PLEKHM1	1.41	-0.49	-0.88	-0.04	6
PPDPF	1.43	-0.18	-0.88	-0.37	6
PRKAG1	1.34	0.19	-0.84	-0.69	6
PRUNE2	1.44	-0.09	-0.75	-0.60	6
PTGES2	1.30	0.22	-0.99	-0.53	6
RASSF3	1.42	-0.02	-0.73	-0.67	6
RDH10	0.78	0.15	0.52	-1.45	6
RHOT1	1.34	0.17	-0.87	-0.64	6
RIPOR3	1.14	-0.93	0.53	-0.74	6
RN7SL2	1.40	-0.69	-0.75	0.04	6
RSAD1	1.32	0.22	-0.88	-0.66	6
SEMA3A	0.80	0.32	0.35	-1.46	6
SLC25A1	1.31	-1.12	-0.02	-0.17	6
SMARCA5	0.60	0.67	0.20	-1.47	6
SMG5	1.40	-0.08	-0.95	-0.37	6
SNHG4	0.71	0.79	-0.14	-1.36	6
ST8SIA4	1.10	-0.45	0.51	-1.16	6
TAB1	1.40	-0.94	-0.07	-0.39	6
TANC1	1.45	-0.67	-0.12	-0.65	6
TKT	1.39	0.03	-0.51	-0.91	6
TNIK	1.20	-1.21	0.24	-0.23	6
TRAF7	1.47	-0.20	-0.59	-0.67	6
VARS	1.41	0.01	-0.72	-0.71	6
VPS11	1.38	-0.25	-1.02	-0.11	6
WWP2	1.47	-0.20	-0.66	-0.60	6
WWTR1	0.57	1.00	-0.32	-1.25	6

ACAT2	1.03	-0.85	-0.87	0.68	7
ACSL4	0.44	-0.94	-0.69	1.20	7
AGAP3	1.20	-0.78	-0.86	0.45	7
ARHGDIA	0.58	-1.46	0.18	0.70	7
C6orf132	0.27	-0.32	-1.16	1.21	7
CBFA2T2	0.22	-0.95	-0.58	1.31	7
CLCN6	1.03	-0.71	-0.99	0.67	7
CLIP3	-0.24	-1.29	0.54	0.99	7
CPSF1	0.84	-1.45	0.36	0.25	7
CTBP1	0.99	-1.39	0.26	0.14	7
CXCL2	0.88	-1.42	0.46	0.09	7
DENND5A	-0.19	-0.96	-0.26	1.41	7
DMKN	-0.17	-1.00	-0.22	1.39	7
DVL1	1.23	-1.18	-0.26	0.21	7
ETFA	0.78	-1.19	-0.47	0.87	7
EVI5L	-0.02	-1.21	0.00	1.23	7
FKBP8	1.17	-0.98	-0.67	0.48	7
FLII	-0.12	-0.89	-0.41	1.42	7
FZR1	0.06	-1.32	0.16	1.10	7
GEM	-0.01	-1.05	-0.29	1.35	7
GNPDA1	0.08	-0.77	-0.69	1.38	7
HMCN1	1.05	-0.96	-0.74	0.66	7
HPS1	-0.01	-0.71	-0.70	1.42	7
IRAK1	0.35	-1.31	-0.12	1.08	7
IRF2BP2	0.60	-0.49	-1.15	1.04	7
KIF13A	0.79	-1.00	-0.72	0.93	7
LHFPL2	-0.07	-1.38	0.59	0.86	7
LSS	1.32	-0.76	-0.79	0.22	7
LTBR	-0.32	-1.06	0.05	1.33	7
MAP2K2	0.14	-1.37	0.20	1.03	7
MAST2	0.50	-1.49	0.63	0.36	7
MCRIP1	1.07	-0.98	-0.72	0.62	7
MICU1	0.81	-1.36	-0.15	0.69	7
MLPH	0.21	-0.42	-1.07	1.28	7
MVB12A	0.91	-1.08	-0.62	0.79	7
MVD	0.91	-0.57	-1.11	0.77	7
NSMAF	1.30	-0.90	-0.66	0.26	7
PARP6	0.00	-0.80	-0.61	1.41	7
PCSK9	0.52	-1.13	-0.48	1.10	7

PCYT2	0.98	-1.20	-0.43	0.65	7
PDE4DIP	0.30	-1.16	-0.34	1.20	7
PLEKHM2	0.26	-0.98	-0.56	1.28	7
RITA1	1.19	-0.81	-0.84	0.46	7
RNF24	0.65	-1.43	0.73	0.05	7
SH2D5	0.25	-0.93	-0.62	1.30	7
SORBS3	-0.17	-1.28	0.36	1.09	7
SPEG	0.02	-1.38	0.40	0.96	7
TECPR1	1.03	-0.93	-0.78	0.68	7
TGFB1I1	0.83	-0.79	-0.94	0.90	7
TSR3	0.84	-0.98	-0.74	0.88	7
TYK2	0.66	-0.83	-0.88	1.05	7
WASHC2C	0.47	-1.13	-0.47	1.13	7
ZFP28	1.00	-0.91	-0.81	0.72	7
ZNF316	-0.24	-1.07	-0.03	1.34	7
ZNF358	0.26	-0.78	-0.78	1.31	7
ZNF558	0.61	-0.76	-0.94	1.09	7
ZYX	1.32	-0.72	-0.83	0.23	7

Supplementary Table S3. Full list of changed pathways due to *CERS2* KD and PMA treatment using the entire LRT gene list with the corresponding clusters labeled (FDR < 0.05).

Pathway	Log_2 [Enrichment Ratio]	$-Log_{10}$ [FDR]	Cluster
Cholesterol biosynthesis	4.12	12.32	1
Interferon alpha/beta signaling	3.07	10.03	2
Activation of gene expression by SREBF (SREBP)	3.37	8.56	6
Cytokine Signaling in Immune system	1.34	7.99	2
Regulation of cholesterol biosynthesis by SREBP (SREBF)	3.08	7.95	6
Interferon Signaling	2.09	7.94	2
Cholesterol biosynthesis	4.27	6.60	1
Focal adhesion	1.81	4.62	4
Steroid biosynthesis	3.61	4.61	1
Metabolism of steroids	1.95	4.35	1
Antiviral mechanism by IFN-stimulated genes	2.38	4.14	2
Immune System	0.65	4.10	2
Influenza A	1.76	3.54	2
OAS antiviral response	4.18	3.42	2
Terpenoid backbone biosynthesis	3.21	3.06	
Measles	1.81	2.79	2
Integrin signalling pathway	1.65	2.68	
Interleukin-4 and Interleukin-13 signaling	1.91	2.63	5
ECM-receptor interaction	2.09	2.59	1
HIF-1 signaling pathway	1.91	2.39	
Extracellular matrix organization	1.27	2.34	3
Proteoglycans in cancer	1.48	2.27	
MicroRNAs in cancer	1.63	2.25	
Metabolism of lipids	0.86	2.25	1
Human papillomavirus infection	1.15	1.99	
Osteoclast differentiation	1.67	1.96	
Pathways in cancer	0.95	1.96	
ISG15 antiviral mechanism	2.03	1.94	2
Integrin cell surface interactions	1.91	1.94	
Epstein-Barr virus infection	1.38	1.83	2
Fatty acid metabolism	2.27	1.83	1
PI3K-Akt signaling pathway	1.09	1.80	

Regulation of Insulin-like Growth Factor (IGF) transport and uptake by Insulin-like Growth Factor Binding Proteins (IGFBPs)	1.59	1.61	4
Bladder cancer	2.31	1.54	5
Hepatitis C	1.52	1.44	2
Herpes simplex infection	1.33	1.40	2
Kaposi sarcoma-associated herpesvirus infection	1.32	1.38	
Elastic fibre formation	2.17	1.34	

Supplementary Table S4. Complete gene list identified as different due to both *CERS5/6* KD and PMA treatment with their respective z-score and cluster.

Gene	Scramble - Vehicle	Scramble - PMA	CERS5/6 KD - Vehicle	CERS5/6 KD - PMA	Cluster
ACAT2	0.01	-0.38	1.37	-1.00	1
COL5A3	0.45	-0.80	1.20	-0.85	1
EPAS1	-0.45	0.89	0.75	-1.20	1
ESYT1	-0.12	0.61	0.87	-1.36	1
FOXRED2	0.05	0.56	0.81	-1.42	1
GAS7	0.07	-0.51	1.37	-0.93	1
GPC1	0.03	0.80	0.59	-1.42	1
HMGCS1	-0.31	0.88	0.71	-1.28	1
L3MBTL2	0.23	0.27	0.92	-1.42	1
LDLR	0.77	-0.48	0.89	-1.18	1
LITAF	0.07	0.92	0.41	-1.41	1
LSS	0.51	0.59	0.39	-1.49	1
MCAM	0.29	0.06	1.02	-1.37	1
MCM5	0.39	0.41	0.69	-1.49	1
MT-ATP6	0.32	0.15	0.94	-1.41	1
MT-CO1	0.62	-0.26	0.93	-1.29	1
MT-ND1	0.04	0.96	0.39	-1.39	1
MVD	0.02	0.84	0.55	-1.41	1
MYRF	0.66	0.64	0.16	-1.46	1
NNMT	1.02	0.08	0.27	-1.37	1
PCYT2	0.11	1.04	0.21	-1.36	1
PRUNE2	-0.30	0.21	1.24	-1.15	1
SCARB1	0.44	0.27	0.76	-1.47	1
SLC25A1	0.25	-0.43	1.26	-1.08	1
SPARC	1.08	0.07	0.20	-1.34	1
STK40	0.36	-0.60	1.24	-0.99	1
TNFSF10	-0.05	0.92	0.50	-1.38	1
ALDH3B1	-1.45	0.13	0.70	0.62	2
ANKRD1	-1.22	-0.28	0.37	1.13	2
ATP10A	-1.20	-0.30	0.32	1.17	2
ATP8B1	-1.25	0.27	-0.17	1.15	2
CEP170	0.11	-1.34	0.15	1.08	2
CITED2	-1.24	0.38	-0.26	1.12	2
CXCL3	-0.66	-0.99	0.49	1.16	2

DTX3L	-1.22	-0.24	0.29	1.17	2
EIF2AK2	-1.29	-0.20	0.44	1.05	2
EIF3F	-1.46	0.21	0.51	0.74	2
EPB41L3	-0.81	-0.89	0.66	1.05	2
EPHA5	-1.32	-0.16	0.45	1.02	2
EPSTI1	-1.12	-0.56	0.78	0.90	2
ESM1	-0.56	-0.71	-0.19	1.46	2
FGF5	-0.83	-0.43	-0.18	1.44	2
HAS2	-0.64	-1.03	0.57	1.10	2
HELZ2	-1.20	-0.43	0.65	0.97	2
HHEX	0.20	-1.39	0.20	0.99	2
IFI27	-1.07	-0.56	0.50	1.14	2
IFI35	-1.17	-0.44	0.56	1.05	2
IFI44L	-1.01	-0.58	0.36	1.23	2
IFI6	-0.95	-0.60	0.25	1.29	2
IFIH1	-1.07	-0.60	0.63	1.04	2
IFIT5	-1.21	-0.39	0.58	1.03	2
IFITM1	-1.05	-0.48	0.26	1.27	2
IL33	-0.50	-1.15	0.65	1.00	2
INSYN2B	-0.38	-0.74	-0.35	1.48	2
IRF7	-1.13	-0.54	0.71	0.96	2
LAP3	-1.12	-0.56	0.78	0.90	2
MX1	-1.08	-0.57	0.55	1.10	2
MX2	-1.07	-0.62	0.65	1.03	2
NPPB	-1.14	-0.13	-0.03	1.30	2
NT5E	-0.76	-0.64	-0.02	1.42	2
OAS1	-1.07	-0.59	0.57	1.09	2
OAS2	-1.11	-0.58	0.72	0.96	2
OAS3	-1.13	-0.52	0.61	1.04	2
PARP12	-1.20	-0.29	0.34	1.16	2
PARP14	-1.27	-0.27	0.49	1.04	2
PARP9	-1.15	-0.46	0.51	1.10	2
PATL1	-1.44	0.10	0.65	0.70	2
PHLDA1	-0.82	-0.59	0.00	1.41	2
PLAT	-0.80	-0.84	0.41	1.22	2
PLSCR1	-1.13	-0.46	0.46	1.14	2
SAMHD1	-1.26	-0.34	0.69	0.90	2
SELENOT	-0.34	-0.86	-0.24	1.44	2
SERPINB2	-0.88	-0.43	-0.11	1.42	2

SGK1	-0.76	-0.64	-0.03	1.42	2
SLC15A3	-1.31	-0.05	0.26	1.10	2
SLC7A2	-0.74	-0.49	-0.23	1.47	2
SP100	-0.94	-0.16	-0.32	1.41	2
STARD4	-1.35	-0.08	0.43	1.00	2
STAT1	-1.22	-0.29	0.38	1.13	2
STAT2	-1.35	0.40	-0.07	1.01	2
SVIP	-0.09	-1.27	0.20	1.16	2
TFPI2	-0.74	-0.47	-0.27	1.47	2
TM4SF1	-0.87	-0.31	-0.27	1.44	2
TNFAIP3	-0.81	-0.84	0.45	1.20	2
TRIM22	-1.09	-0.53	0.49	1.14	2
TRIM25	-1.35	-0.15	0.60	0.90	2
UAP1L1	-1.32	0.48	-0.16	1.00	2
UNC93B1	-1.32	0.05	0.16	1.11	2
XAF1	-1.30	-0.11	0.32	1.09	2
ABTB1	-0.54	0.15	-0.95	1.34	3
AC025419.1	-0.08	0.62	-1.38	0.84	3
ACAA2	-0.01	-0.19	-1.11	1.31	3
ADAMTS6	-0.57	0.14	-0.92	1.35	3
AKAP12	-0.80	-0.08	-0.55	1.43	3
ANGPTL4	-0.51	-0.02	-0.88	1.40	3
ANPEP	-0.28	-0.02	-1.05	1.35	3
ANTXR2	-0.47	0.37	-1.10	1.20	3
AP000695.2	0.05	0.68	-1.43	0.70	3
APBB2	-0.49	0.26	-1.04	1.27	3
APLP2	-0.81	-0.16	-0.48	1.45	3
ARHGAP22	0.26	0.19	-1.41	0.96	3
ATP13A3	-0.75	0.50	-0.91	1.16	3
ATP2A3	-0.68	0.02	-0.74	1.41	3
BMP2	-0.78	0.48	-0.88	1.18	3
CBLB	-0.81	0.36	-0.80	1.25	3
CDCP1	-0.54	0.61	-1.11	1.05	3
CDH2	-0.24	0.13	-1.15	1.27	3
CSF2	-0.54	-0.32	-0.62	1.49	3
CSF3	-0.46	-0.33	-0.69	1.48	3
CTSS	-0.70	0.39	-0.92	1.23	3
CXCL8	-0.83	-0.07	-0.53	1.43	3
DDIT4L	-0.71	-0.27	-0.50	1.48	3

ERRFI1	0.20	0.47	-1.46	0.78	3
FOXP1	-0.63	0.23	-0.91	1.31	3
GDF15	-1.16	0.33	-0.37	1.19	3
GJA1	-0.37	-0.54	-0.58	1.49	3
IER3	-0.37	0.87	-1.24	0.75	3
IL11	-0.50	0.09	-0.95	1.36	3
IL1A	-0.75	-0.01	-0.65	1.42	3
IL1B	-0.72	0.18	-0.81	1.34	3
INHBA	-0.73	0.55	-0.95	1.13	3
JAK1	-0.38	0.81	-1.24	0.81	3
MEDAG	-0.18	0.58	-1.33	0.94	3
MMP1	-0.78	0.13	-0.71	1.37	3
MMP3	-0.67	0.07	-0.79	1.39	3
MSN	0.71	-0.54	-1.13	0.96	3
MT1E	-0.69	0.41	-0.93	1.22	3
MYCT1	-0.65	-0.27	-0.55	1.48	3
NDRG1	-0.69	0.43	-0.94	1.21	3
NOX4	-0.34	0.59	-1.24	1.00	3
NRP2	-0.73	0.59	-0.96	1.10	3
PLA2G4A	-0.72	-0.34	-0.42	1.48	3
PLAUR	-0.45	-0.27	-0.75	1.47	3
PLIN2	-0.76	0.55	-0.92	1.13	3
PODXL	-0.65	0.56	-1.02	1.11	3
PTGS2	-0.71	-0.35	-0.41	1.48	3
PTPRK	-0.47	-0.35	-0.66	1.49	3
SASH1	0.01	-0.71	-0.71	1.41	3
SERPIND1	0.53	-0.66	-1.00	1.13	3
SERPINE1	-0.73	0.79	-0.99	0.93	3
SERPINE2	-0.76	0.33	-0.84	1.27	3
SPRED1	0.29	0.15	-1.40	0.96	3
STARD13	0.10	-0.36	-1.06	1.32	3
STC1	-0.80	0.44	-0.84	1.21	3
STXBP5-AS1	-0.31	0.23	-1.15	1.24	3
TCF4	0.09	0.26	-1.37	1.02	3
TSPAN13	-0.08	0.34	-1.32	1.07	3
TXNIP	-1.01	0.61	-0.67	1.08	3
UTP25	-0.78	0.27	-0.79	1.30	3
ZBTB38	-0.43	0.73	-1.21	0.91	3
AC013451.2	1.14	0.43	-1.17	-0.40	4

ADAM19	0.82	0.19	-1.45	0.44	4
ADGRL4	0.74	0.61	-1.44	0.09	4
AK4	0.94	0.70	-1.20	-0.44	4
AMIGO2	0.03	1.08	-1.33	0.22	4
APCDD1L	1.20	0.44	-0.90	-0.74	4
APCDD1L-DT	1.19	0.44	-1.00	-0.62	4
BHLHE40	0.34	0.90	-1.43	0.19	4
BNIP3	0.44	0.81	-1.45	0.20	4
BNIP3L	0.51	1.06	-1.22	-0.35	4
CA12	1.17	0.50	-0.90	-0.77	4
CAPRIN2	1.35	-0.11	-1.06	-0.18	4
CD44	1.39	-0.06	-0.98	-0.35	4
CHST11	0.39	0.94	-1.40	0.08	4
CNIH3	0.08	0.88	-1.42	0.45	4
COL13A1	1.02	0.45	-1.32	-0.14	4
COL6A2	1.22	0.41	-0.83	-0.81	4
COL7A1	1.13	0.37	-1.22	-0.28	4
CXCL5	0.54	1.02	-1.26	-0.30	4
DAAM1	0.14	0.86	-1.43	0.43	4
DCBLD2	0.51	0.66	-1.48	0.31	4
DRP2	0.72	0.39	-1.48	0.37	4
DSE	0.05	0.83	-1.42	0.55	4
ELL2	0.53	0.79	-1.44	0.11	4
EPHB2	1.31	0.23	-0.93	-0.61	4
F2R	0.87	0.82	-1.09	-0.60	4
F2RL1	1.02	0.64	-1.14	-0.51	4
FHOD3	0.89	0.63	-1.33	-0.19	4
HHIP	0.75	0.89	-1.21	-0.42	4
HHIP-AS1	0.77	0.72	-1.37	-0.12	4
HIC1	1.08	0.47	-1.23	-0.32	4
ITGA2	0.51	0.44	-1.50	0.54	4
KDM3A	0.50	0.68	-1.48	0.30	4
KDR	0.58	0.92	-1.34	-0.16	4
LAMA4	0.03	0.77	-1.42	0.62	4
LDHA	1.15	0.53	-0.88	-0.80	4
LIPG	1.35	0.16	-0.79	-0.73	4
LPCAT2	0.27	1.12	-1.29	-0.10	4
LRRC15	0.87	0.81	-1.13	-0.55	4

LTBP2	0.77	0.94	-1.06	-0.65	4
MMP14	0.50	0.99	-1.32	-0.17	4
MPP4	0.63	0.00	-1.41	0.78	4
NRP1	0.75	0.85	-1.27	-0.33	4
P4HA1	0.69	0.96	-1.18	-0.47	4
PACS1	0.40	0.40	-1.49	0.69	4
PAPPA	0.30	0.95	-1.41	0.16	4
PDCD4	0.17	0.78	-1.45	0.50	4
PDGFRA	0.00	1.00	-1.37	0.37	4
PKIA	1.02	0.52	-1.27	-0.27	4
PLA2R1	0.95	0.74	-1.09	-0.61	4
PLOD2	1.20	0.22	-1.20	-0.23	4
PPFIA4	0.69	0.88	-1.29	-0.29	4
PPFIBP1	0.28	0.95	-1.41	0.19	4
PPP1R3B	0.14	1.12	-1.32	0.06	4
PPP3CA	1.03	0.68	-0.96	-0.75	4
PRKAA1	0.72	0.84	-1.31	-0.25	4
PTGS1	0.86	0.45	-1.43	0.13	4
RALA	1.03	0.18	-1.37	0.16	4
RIN2	0.07	0.97	-1.39	0.35	4
SEMA7A	1.11	0.46	-1.19	-0.38	4
SIPA1L1	0.65	0.18	-1.46	0.63	4
SKIL	0.52	1.10	-1.13	-0.49	4
SREK1IP1	0.63	0.60	-1.48	0.25	4
SRPX	0.71	0.45	-1.48	0.33	4
TFPI	0.96	0.68	-1.20	-0.44	4
TGFBI	1.19	0.47	-0.80	-0.85	4
TLR4	0.90	0.60	-1.34	-0.16	4
TNFSF15	1.26	0.35	-0.88	-0.73	4
TPBG	0.15	0.64	-1.46	0.67	4
TPM1	0.66	0.99	-1.16	-0.50	4
VMP1	0.48	0.85	-1.43	0.10	4
ADM	-1.48	0.72	0.38	0.38	5
AP000695.1	-0.16	1.02	-1.31	0.45	5
ARHGEF17	-0.40	1.43	-0.88	-0.15	5
ARNT2	-0.62	1.38	-0.84	0.08	5
C1orf198	-1.19	0.93	-0.46	0.71	5
CALR	-0.69	1.37	-0.80	0.13	5
CANX	-0.68	1.31	-0.88	0.25	5

CCND1	-0.44	1.08	-1.16	0.52	5
CDK2AP2	-0.77	1.35	-0.74	0.16	5
CLPTM1L	-1.39	0.96	0.05	0.39	5
CNN2	-1.05	1.36	-0.15	-0.15	5
CRELD2	-0.63	1.43	-0.74	-0.06	5
CTSD	-0.97	1.06	-0.73	0.64	5
FADS3	-1.36	0.99	-0.05	0.42	5
FAM129A	-1.32	0.91	-0.20	0.61	5
FAP	-0.37	1.21	-1.14	0.30	5
HERPUD1	-0.51	1.39	-0.89	0.01	5
HM13	-0.92	1.22	-0.71	0.40	5
HSP90B1	-0.56	1.36	-0.91	0.12	5
HSPA5	-0.60	1.38	-0.85	0.06	5
HYOU1	-0.72	1.39	-0.73	0.06	5
KIT	-1.14	1.22	-0.37	0.28	5
KLF11	-0.98	1.17	-0.67	0.47	5
LIMS1	-0.27	1.11	-1.24	0.40	5
MAGT1	-0.65	1.42	-0.75	-0.03	5
MANF	-0.78	1.34	-0.75	0.20	5
MFGE8	-1.14	0.95	-0.54	0.72	5
MMP2	-0.14	1.20	-1.23	0.16	5
MSC	-0.54	1.19	-1.06	0.41	5
MYLK	-0.50	1.46	-0.76	-0.20	5
NTM	-0.31	1.01	-1.25	0.55	5
OGFRL1	-1.29	1.11	-0.11	0.29	5
PCDH1	-0.57	1.08	-1.09	0.57	5
PCK2	-1.12	1.28	-0.30	0.13	5
PDGFB	-0.49	1.19	-1.09	0.39	5
PDIA4	-0.69	1.37	-0.79	0.11	5
PER1	-1.38	1.00	0.31	0.06	5
PLXNB2	-1.18	1.26	0.03	-0.12	5
PTPRB	-0.75	0.92	-0.97	0.80	5
PTPRR	-0.55	1.14	-1.08	0.48	5
SCG5	-0.81	0.85	-0.92	0.88	5
SDF2L1	-0.93	1.30	-0.62	0.25	5
SEC61A1	-0.64	1.34	-0.88	0.18	5
SLC1A4	-0.90	1.32	-0.63	0.21	5
SLC1A5	-0.80	1.38	-0.66	0.08	5
SLC4A2	-1.07	1.28	-0.41	0.21	5

SLFN11	-0.72	1.34	-0.81	0.19	5
SQLE	-0.73	1.40	-0.71	0.03	5
STC2	-0.44	0.98	-1.19	0.66	5
TMED2	-0.60	1.40	-0.83	0.03	5
TMEM97	-1.26	1.12	-0.21	0.35	5
VSIR	-1.27	1.17	0.01	0.10	5
VWA8	-0.38	0.92	-1.24	0.70	5
WARS	-1.06	1.35	-0.19	-0.10	5
WFS1	-0.83	1.41	-0.56	-0.02	5
ACTA2	-0.90	-0.62	1.33	0.19	6
ACTN1	-1.34	0.10	1.08	0.16	6
ADAM15	-1.23	-0.18	1.19	0.21	6
APOL6	-0.90	-0.71	1.24	0.38	6
ARHGAP23	-1.32	0.34	1.06	-0.08	6
AXL	-0.72	-0.61	1.45	-0.12	6
BAMBI	-1.11	-0.32	1.28	0.16	6
CCBE1	-0.54	-0.67	1.48	-0.28	6
CCL5	-0.77	-0.77	1.33	0.20	6
COL4A4	-1.30	-0.21	1.02	0.49	6
COL5A2	0.21	-0.88	1.32	-0.66	6
COL8A1	-0.95	-0.46	1.37	0.03	6
DAG1	-1.25	-0.12	1.18	0.19	6
DRAXIN	-1.18	0.03	1.27	-0.12	6
EEF2	-1.11	-0.55	1.02	0.65	6
EHD1	-0.13	-0.88	1.43	-0.41	6
EPS8L2	-1.17	-0.40	1.14	0.42	6
FBN2	-0.17	-0.48	1.45	-0.79	6
GPRC5A	-0.89	-0.70	1.27	0.33	6
GPRC5B	-1.44	0.11	0.79	0.54	6
HERC5	-0.81	-0.84	1.20	0.44	6
HGF	-0.18	-1.17	1.27	0.08	6
IDH2	-1.38	0.01	0.96	0.40	6
IFIT2	-0.56	-0.87	1.39	0.05	6
IGFBP5	-1.05	-0.64	1.00	0.69	6
ITPK1	-0.76	-0.71	1.38	0.09	6
LIMD1	-1.21	0.84	0.80	-0.42	6
MAPK11	-1.18	-0.15	1.26	0.07	6
MARCHF4	-1.01	-0.68	1.06	0.62	6
MATN2	-0.67	-0.80	1.38	0.08	6

MTSS1L	-0.55	-0.24	1.47	-0.68	6
MYBL2	-0.18	-0.83	1.45	-0.44	6
MYL9	-0.83	-0.38	1.45	-0.24	6
NOV	-0.77	-0.69	1.39	0.08	6
NR2F1	-0.36	-0.76	1.47	-0.35	6
PHLDA3	-1.09	-0.57	1.06	0.60	6
PLEKHA4	-0.97	-0.53	1.32	0.19	6
PPM1H	-1.17	0.42	1.14	-0.38	6
PTPRF	-1.01	-0.60	1.21	0.40	6
QSOX1	-0.53	-0.69	1.47	-0.25	6
RAB36	-1.40	0.79	0.64	-0.02	6
RMND5B	-0.65	-0.34	1.49	-0.50	6
RRM2	-0.83	-0.74	1.30	0.27	6
RSAD2	-0.69	-0.81	1.36	0.14	6
S1PR1	-0.68	-0.98	1.16	0.50	6
SAMD9L	-1.05	-0.58	1.15	0.48	6
SCG2	-0.14	-0.59	1.45	-0.72	6
SEMA3C	0.08	-1.44	0.71	0.65	6
SLC2A13	-1.44	0.65	0.69	0.10	6
SOX13	-0.60	-0.48	1.50	-0.41	6
TAGLN	-0.91	-0.63	1.31	0.22	6
THEM6	-1.15	-0.29	1.25	0.20	6
TNFSF4	-1.41	0.22	0.96	0.24	6
ARRDC3	0.38	-1.49	0.67	0.44	7
B3GNT5	0.94	-0.87	-0.86	0.79	7
CDK17	1.14	-1.29	0.00	0.15	7
CHST2	0.27	-1.48	0.71	0.50	7
COL6A1	1.34	0.15	-0.54	-0.95	7
CXCL1	1.49	-0.60	-0.36	-0.53	7
CXCL6	1.21	-0.95	0.41	-0.68	7
DIO2	1.48	-0.51	-0.29	-0.69	7
DOCK4	0.48	-1.50	0.56	0.46	7
FGGY	1.29	-0.47	-1.04	0.21	7
FOXC2	0.73	-1.43	0.04	0.65	7
FZD8	0.98	-1.08	-0.61	0.71	7
GALNT6	1.48	-0.71	-0.34	-0.43	7
GFPT2	1.39	-0.01	-0.47	-0.92	7
HLTF	1.43	-0.06	-0.80	-0.56	7
JUN	1.46	-0.58	-0.18	-0.70	7

LAMC2	0.80	-1.18	-0.48	0.86	7
LRRC17	1.29	-0.83	0.28	-0.74	7
MEST	1.39	-0.13	-0.27	-0.99	7
PFKFB3	1.33	-1.06	0.07	-0.34	7
RGS4	0.63	-1.07	1.05	-0.61	7
SDCCAG8	0.41	-1.22	-0.32	1.12	7
SMURF2	1.43	-0.44	-0.86	-0.14	7
SSH2	0.94	-1.35	0.51	-0.10	7
TNFRSF10D	1.44	-0.10	-0.58	-0.76	7
VCAN	1.41	0.02	-0.73	-0.70	7
AARS	0.02	1.38	-0.95	-0.44	8
AJUBA	-0.23	1.38	-0.13	-1.02	8
CCDC80	-0.21	1.47	-0.65	-0.61	8
CCL11	0.18	1.26	-1.15	-0.29	8
CLDN11	0.11	1.27	-0.22	-1.16	8
CTHRC1	0.18	1.26	-1.14	-0.30	8
CXXC5	0.46	1.18	-0.67	-0.98	8
DAB2	-0.20	1.46	-0.77	-0.49	8
DHCR24	-0.62	1.42	-0.02	-0.77	8
DHCR7	-0.22	1.43	-0.29	-0.91	8
DNAJB11	-0.34	1.47	-0.76	-0.37	8
DNAJC3	0.38	1.21	-0.56	-1.03	8
EDIL3	0.22	1.32	-0.64	-0.90	8
EFEMP1	0.57	1.01	-0.34	-1.24	8
EMILIN1	0.05	1.39	-0.83	-0.61	8
ENAH	0.20	1.33	-0.82	-0.72	8
EPHX1	-0.40	1.50	-0.60	-0.49	8
FADS1	-0.40	1.49	-0.61	-0.48	8
FADS2	0.50	1.14	-0.56	-1.07	8
HDLBP	-0.51	1.49	-0.64	-0.34	8
HSPB1	-0.23	1.47	-0.71	-0.53	8
INSIG1	0.72	0.99	-0.70	-1.01	8
ITGA3	0.40	1.12	-0.29	-1.23	8
KRT80	0.01	1.41	-0.79	-0.62	8
MEGF10	0.42	1.18	-1.08	-0.53	8
MLEC	0.19	1.32	-0.94	-0.58	8
NANOS1	0.04	1.39	-0.60	-0.84	8
NUPR1	-0.09	1.43	-0.85	-0.49	8
PAPSS2	-0.34	1.21	0.29	-1.15	8

PARVA	-0.16	1.46	-0.58	-0.72	8
PDP2	0.39	1.23	-0.73	-0.89	8
PER3	0.42	1.22	-0.77	-0.86	8
PLEKHA6	0.48	1.12	-0.47	-1.13	8
PPL	0.45	1.20	-0.80	-0.85	8
PSAT1	-0.10	1.44	-0.77	-0.58	8
PYCR1	-0.56	1.26	0.31	-1.00	8
RBM24	-0.42	1.48	-0.69	-0.37	8
SCD	0.70	1.01	-0.74	-0.97	8
SEL1L3	0.53	1.14	-0.73	-0.94	8
SERPINH1	-0.10	1.42	-0.88	-0.44	8
SHC1	0.07	1.15	0.06	-1.29	8
SHMT2	-0.47	1.50	-0.59	-0.44	8
SLC7A11	-0.23	1.47	-0.52	-0.72	8
SREBF1	1.01	0.60	-0.40	-1.21	8
TMEM42	-0.63	1.48	-0.27	-0.59	8
TNS2	-0.59	1.38	0.07	-0.86	8
TRIB3	-0.27	1.44	-0.89	-0.28	8
TXNRD1	-0.14	1.44	-0.81	-0.49	8
VASN	-0.44	1.49	-0.68	-0.36	8
ZBED3	-0.58	1.35	0.13	-0.91	8

Supplementary Table S5. Complete list of pathways changed due to *CERS5/6* KD and PMA treatment using the entire LRT gene list with the corresponding clusters labeled (FDR < 0.05).

Pathway	Log_2 [Enrichment Ratio]	$-Log_{10}$ [FDR]	Cluster
Extracellular matrix organization	2.21	10.61	4
Interferon alpha/beta signaling	3.25	8.89	2
Cytokine Signaling in Immune system	1.55	8.89	3
Influenza A	2.31	6.56	2
Interferon Signaling	2.17	6.21	2
IL-17 signaling pathway	2.73	6.20	3
Collagen degradation	2.97	5.67	
Measles	2.40	5.67	2
Degradation of the extracellular matrix	2.31	5.31	
Collagen formation	2.59	4.82	4
NOD-like receptor signaling pathway	2.05	4.17	2
Rheumatoid arthritis	2.48	4.05	3
Interleukin-4 and Interleukin-13 signaling	2.32	3.93	3
Regulation of Insulin-like Growth Factor (IGF) transport and uptake by Insulin-like Growth Factor Binding Proteins (IGFBPs)	2.21	3.92	5
Collagen biosynthesis and modifying enzymes	2.67	3.83	4
Plasminogen activating cascade	3.95	3.80	3
Integrin cell surface interactions	2.45	3.65	4
Cholesterol biosynthesis	3.44	3.55	1
Interleukin-10 signaling	2.89	3.52	3
Assembly of collagen fibrils and other multimeric structures	2.66	3.47	
Signaling by Interleukins	1.33	3.47	3
ECM proteoglycans	2.48	3.43	
Focal adhesion	1.81	3.42	
MET activates PTK2 signaling	3.17	3.10	
Activation of gene expression by SREBF (SREBP)	2.88	3.06	1
Regulation of cholesterol biosynthesis by SREBP (SREBF)	2.66	3.06	1
Dissolution of Fibrin Clot	3.89	3.01	3
Collagen chain trimerization	2.81	2.95	
Signaling by PDGF	2.58	2.92	
Non-integrin membrane-ECM interactions	2.56	2.87	6
Post-translational protein phosphorylation	2.10	2.81	

Integrin signalling pathway	1.80	2.76	
TNF signaling pathway	2.08	2.76	
Antiviral mechanism by IFN-stimulated genes	2.31	2.71	2
Hepatitis C	1.94	2.68	2
Unfolded Protein Response (UPR)	2.13	2.62	5
Immune System	0.61	2.62	2
AGE-RAGE signaling pathway in diabetic complications	2.10	2.56	3
Signaling by Receptor Tyrosine Kinases	1.20	2.55	
MET promotes cell motility	2.72	2.38	
Cytokine-cytokine receptor interaction	1.39	2.38	3
Herpes simplex infection	1.65	2.32	2
Kaposi sarcoma-associated herpesvirus infection	1.64	2.31	
Laminin interactions	2.95	2.31	
Interleukin-12 family signaling	2.41	2.26	
Interferon gamma signaling	2.07	2.22	2
PI3K-Akt signaling pathway	1.26	2.21	
CCKR signaling map	1.65	2.17	3
Amoebiasis	2.01	2.10	
Cholesterol biosynthesis	3.69	2.10	1
Hematopoietic cell lineage	1.99	2.07	3
Pathways in cancer	1.05	2.03	
ECM-receptor interaction	2.08	1.98	
Protein processing in endoplasmic reticulum	1.61	1.85	5
Salmonella infection	2.02	1.84	3
Blood coagulation	2.61	1.82	
Metabolism of steroids	1.63	1.70	1
TGF-beta signaling pathway	1.93	1.68	
Leishmaniasis	2.06	1.64	3
Epstein-Barr virus infection	1.43	1.57	2
Pertussis	2.02	1.57	
IRE1alpha activates chaperones	2.20	1.56	5
Angiogenesis	1.55	1.55	
OAS antiviral response	3.86	1.54	2
Regulation of cytoskeletal remodeling and cell spreading by IPP complex components	3.86	1.54	
PPARA activates gene expression	1.72	1.53	
Fluid shear stress and atherosclerosis	1.62	1.53	
Signaling by MET	1.97	1.51	

Regulation of lipid metabolism by Peroxisome proliferator-activated receptor alpha (PPARalpha)	1.70	1.49	
Nicotinate metabolism	2.64	1.47	2
Steroid biosynthesis	3.02	1.42	
Nicotinamide salvaging	3.02	1.42	
Chemokine receptors bind chemokines	2.27	1.39	
Interleukin-12 signaling	2.27	1.39	
Hemostasis	0.85	1.37	
